# Interred mechanisms of resistance and host immune evasion revealed through network-connectivity analysis of *M. tuberculosis* complex graph pangenome

**DOI:** 10.1128/msystems.00499-24

**Published:** 2025-03-06

**Authors:** Monica E. Espinoza, Ashley M. Swing, Afif Elghraoui, Samuel J. Modlin, Faramarz Valafar

**Affiliations:** 1 Laboratory for Pathogenesis of Clinical Drug Resistance and Persistence, San Diego State University, San Diego, California, USA; 2 San Diego State University/University of California, San Diego | Joint Doctoral Program in Public Health (Global Health), San Diego, California, USA; 3 Department of Electrical and Computer Engineering, San Diego State University, San Diego, California, USA; 4 Department of Electrical and Computer Engineering, University of California San Diego, San Diego, California, USA; University of Illinois at Chicago, Chicago, Illinois, USA

**Keywords:** pangenome, tuberculosis, drug resistance, immune evasion, genetic diversity, network analysis, Shannon’s entropy

## Abstract

*Mycobacterium tuberculosis* complex successfully adapts to environmental pressures through mechanisms of rapid adaptation which remain poorly understood despite knowledge gained through decades of research. In this study, we used 110 reference-quality, complete *de novo* assembled, long-read sequenced clinical genomes to study patterns of structural adaptation through a graph-based pangenome analysis, elucidating rarely studied mechanisms that enable enhanced clinical phenotypes offering a novel perspective to the species' adaptation. Across isolates, we identified a pangenome of 4,325 genes (3,767 core and 558 accessory), revealing 290 novel genes, and a substantially more complete account of difficult-to-sequence *esx/pe/pgrs/ppe* genes. Seventy-four percent of core genes were deemed non-essential *in vitro*, 38% of which support the pathogen’s survival *in vivo*, suggesting a need to broaden current perspectives on essentiality. Through information-theoretic analysis, we reveal the *ppe* genes that contribute most to the species’ diversity—several with known consequences for antigenic variation and immune evasion. Construction of a graph pangenome revealed topological variations that implicate genes known to modulate host immunity (*Rv0071-73, Rv2817c, cas2*), defense against phages/viruses (*cas2, csm6,* and *Rv2817c-2821c*), and others associated with host tissue colonization. Here, the prominent trehalose transport pathway stands out for its involvement in caseous granuloma catabolism and the development of post-primary disease. We show paralogous duplications of genes implicated in bedaquiline (*mmpL5* in all L1 isolates) and ethambutol (*embC-A*) resistance, with a paralogous duplication of its regulator (*embR*) in 96 isolates. We provide hypotheses for novel mechanisms of immune evasion and antibiotic resistance through gene dosing that can escape detection by molecular diagnostics.

**IMPORTANCE:**

*M. tuberculosis* complex (MTBC) has killed over a billion people in the past 200 years alone and continues to kill nearly 1.5 million annually. The pathogen has a versatile ability to diversify under immune and drug pressure and survive, even becoming antibiotic persistent or resistant in the face of harsh chemotherapy. For proper diagnosis and design of an appropriate treatment regimen, a full understanding of this diversification and its clinical consequences is desperately needed. A mechanism of diversification that is rarely studied systematically is MTBC’s ability to structurally change its genome. In this article, we have *de novo* assembled 110 clinical genomes (the largest *de novo* assembled set to date) and performed a pangenomic analysis. Our pangenome provides structural variation-based hypotheses for novel mechanisms of immune evasion and antibiotic resistance through gene dosing that can compromise molecular diagnostics and lead to further emergence of antibiotic resistance.

## INTRODUCTION

*Mycobacterium tuberculosis* complex (MTBC) pathogens remain among the deadliest infectious agents in the world, with *Mycobacterium tuberculosis*, the causative agent of tuberculosis (TB) disease (1), alone killing an estimated 1.5 million people annually (2). With no effective vaccine (2–4), TB has cost the lives of over one billion people in the last 200 years (5).

MTBC strains exceptionally evade host immunity and resist antibiotic treatment. The range of phenotypic presentations (including immune evasion) by this pathogen cannot be explained by known single nucleotide polymorphisms (SNPs) alone, warranting more investigation of the full spectrum of genetic determinants of its diversity (6–8). While most projects seek SNPs to explain this phenotypic diversity, previous reports show that gene loss/gain can cause phenotypic change (9, 10). Gene gain mechanisms in prokaryotes generally rely on acquiring external genetic content through horizontal gene transfer, duplication (11), and gene recombination (12). While MTBC lacks horizontal acquisition of genetic material (12–14), it is hypothesized to have more genetic diversity than expected (15). Gene duplication and recombination have been observed in MTBC clinical strains (16, 17). Duplication events are known drivers of diversity in gene families and are subject to recombination with local homologous genes (18). These gain events inevitably result in several fates: maintenance of exact copies, partitioned function (subfunctionalization), new function (neofunctionalization), or loss of function (nonfunctionalization) (19).

Gene loss is also a known mechanism of diversity in MTBC (20) and encompasses frameshift mutations and structural variants (SVs) (both large and small) including deletions. Many studies have identified specific deletions, termed RvD1-6 and TbD1, in the most studied *M. tuberculosis* reference strain, H37Rv, with respect to *Mycobacterium* human-adapted lineages (21, 22). These large deletions and others (23) have been found in many clinical isolates. The reported differences between genomes are posited to be ancestrally acquired and have since been lost in some strains, as was the case with H37Rv (24, 25).

While a few studies have employed long-read sequencing (26–30), many have used short-read sequencing to assess the genomic diversity of *M. tuberculosis* through the construction of its pangenome. Short-read sequencing imposes limitations that might not be overcome post-sequencing (20, 26, 30–33). Short-read sequencing has a well-documented systematic bias that can introduce indels in some genomic regions (34) and does not capture sequence information in other genomic regions (35). As a result, it might be difficult to capture repetitive (35–37) or GC-rich (35, 38) regions (well-known genomic signatures of MTBC including the canonical and difficult-to-characterize *pe/ppe* families (35, 39)) and preclude complete *de novo* assembly, instead resulting in dozens to hundreds of contigs. This necessitates assumptions about the structure of the genome, using a reference, that may be unwarranted. Similarly, important events such as duplications can go unnoticed as short reads cannot always resolve the locus of homologous sequences (e.g., duplications) using their flanking regions. Alternative methods for processing these data, such as reference-based alignment, are similarly hindered (40), and previous work from our group has corrected reference-based calls in the past (35). SV detection using reference-aligned short reads has substantially lower sensitivity than long reads (41, 42). Overall, there is an opportunity for long-read sequenced, *de novo* assembled genomes to reveal distinct areas of genetic diversity yet unexplored in the MTBC pangenome.

Long-read sequencing enables the complete assembly of genomes *de novo* (without assumption of genomic structure) which can provide a complete assessment of genomic diversity both in terms of short and long SVs. *De novo* assembled MTBC genomes improve the detection of larger SVs, resolve the exact location of homologous regions including exact duplications, and, when the sequencing is properly performed (43), comprehensively identify insertions and deletions with minimal error. Studies of a limited number of *de novo* assembled MTBC genomes revealed such genomic changes (44–47). A few pangenome studies employing long-read sequencing of different constituents of MTBC, specific lineages of *M. tuberculosis,* and causative agents for cutaneous tuberculosis have demonstrated the benefits of long-read sequencing for characterizing genetic diversity (26–30). Application of long-read sequencing to *M. tuberculosis* for distinct purposes separate from pangenome analyses has additionally been found, showing the promise of such technology to many fields of tuberculosis research (48–51). It should be considered that long-read sequencing can incur a high cost per base, misidentify single-base variants at a considerable rate, and reach low read depth if one is not careful. However, our study contributes to past genome comparison studies (26, 29, 52–57), which have employed some *de novo* assembled genomes, to accurately describe the diversity of MTBC in these difficult-to-characterize regions.

The foci of recent pangenome studies are diverse, spanning the discovery of vaccine candidates, drug targets, and antibiotic resistance targets (56–62) (Data Set S1). Many studies focus on the pangenomes of specific lineages, comparative genomics across infection sites (63, 64), or include non-tubercule or animal-adapted constituents of MTBC in pangenome calculations (27, 29, 65–77). Other pangenome studies focus solely on specific mechanisms of diversification (20, 26, 30, 32, 33). Pangenome-centered studies previously focused on characterizing pangenomes for specific lineages or assessed genetic diversity across *Mycobacteria* by including isolates from animal-associated *Mycobacteria,* all MTBC-forming bacteria*,* or non*-Mycobacteria* samples in pangenome calculations (29, 54, 66). These studies have also reported whether the shared genetic pool among *Mycobacteria* is open and expanding or closed. While pangenome studies on the shared genetic pool between human-associated and animal-associated pangenomes are important, the inclusion of non-tuberculous *Mycobacteria* strains has reported a smaller and more highly conserved core genome (since conservation is defined as genomic features shared among these varied-host strains). Focusing on these core genomes stands to wash out the clinically relevant genetic diversity and variation of MTBC strains in human infection. This variability could explain differences in virulence, viability in human tissues, and immune evasion, thus limiting efforts to reduce MTBC’s global impact. Also, work reporting the pangenome of specific lineages can help identify new sub-lineages of MTBC and attenuate overestimations of transmission clusters. However, expanded emphasis on genetic variation across lineages of human-adapted MTBC strains could elucidate more global mechanisms of genetic diversity, subsequent evolution, and its contribution to factors such as conferral of drug resistance and persistence.

Our contribution to MTBC pangenome studies is our 109 long-read sequenced, *de novo* assembled *M. tuberculosis* complex genomes, the most reference-quality genomes of *M. tuberculosis* complex published with long-read sequencing. We conducted a pangenome analysis on these highly drug-resistant clinical isolates of *M. tuberculosis sensu stricto*, *M. tuberculosis* var. africanum plus H37Rv reference strain using the tool Panaroo. Panaroo shares information between input genomes using a graph-based method (78). It reports gene adjacency based on gene coordinates existing on the same contig, mending gene mis-annotations with this merged assembly information across genomes, thus improving the accuracy of the estimated core and accessory genomes in comparison to other pangenome construction methods (78). This study is one of the first pangenome network analyses applied to MTBC isolates. These findings not only characterize the different genomic structures and mechanisms of gene gain and loss of clinical MTBC isolates but also shed light on important regions of genomic diversity among genes engaged in host evasion and drug resistance and highlight gaps in our understanding of this pathogen and directions for future study.

## RESULTS

**Fig 1:**
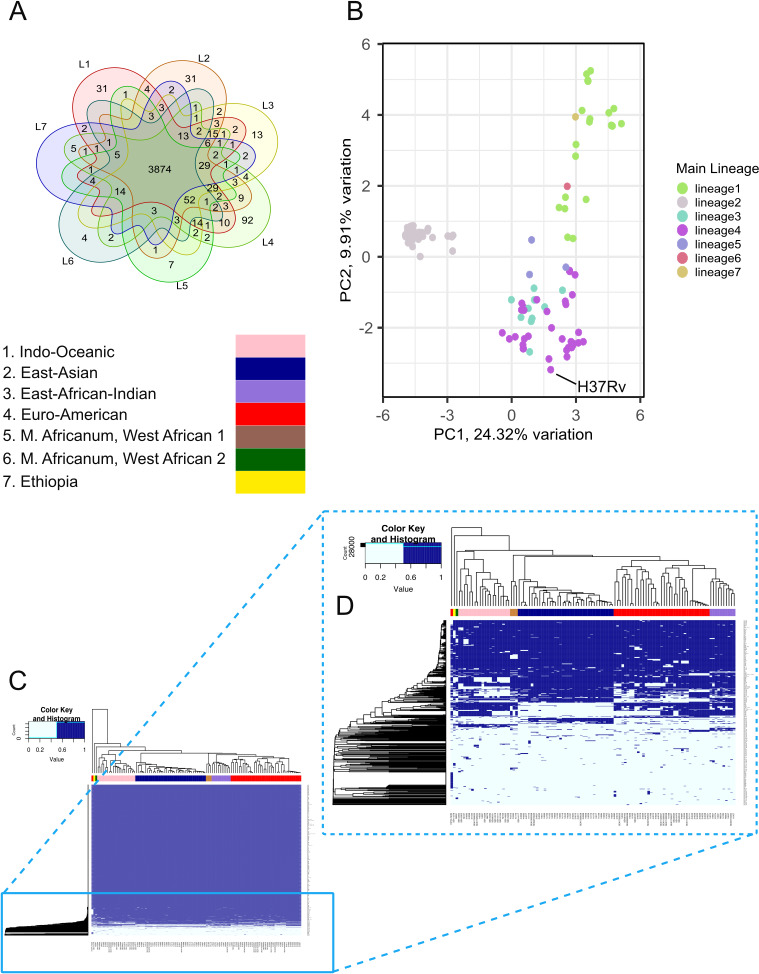
Distribution of genes shared by lineage in the pangenome. (**A**) Diagram showing both the number of genes shared and unique to the lineages in the study sample (*N =* 110 isolates). Lineage-specific pangenome gene lists represent genes that are found in at least one isolate in the lineage. (**B**) Bi-plot of first two principal components, based on the presence/absence of genes in the pangenome, shown by lineage. The location of H37Rv on the bi-plot is indicated by the black line. (**C**) Heat map of full pangenome annotated by lineage. As most of the genes are conserved by all isolates in the pangenome, the distribution of genes in the accessory genome (**D**) is displayed in the outlet.

The pangenome of 109 MTBC clinical isolates and H37Rv contained a total of 4,325 genes, reflecting the total number of genes collectively present in any of the genomes. A vast majority (87%) of the genes were conserved across nearly all isolates, with 3,767 genes observed in at least 99% of the isolates (i.e., core genome). Conversely, 558 genes (13%) were in the accessory genome, representing the genes that are shared by less than 99% of the genomes and reflect the structural genomic diversity of the *M. tuberculosis* complex (Data Set S2). This data set included lineage 1 (L1) isolates (*N* = 20), lineage 2 (L2) isolates (*N* = 37), lineage 3 (L3) isolates (*N* = 10), lineage 4 (L4) isolates (*N* = 38), lineage 5 (L5) isolates (*N* = 5), a lineage 6 (L6) isolate (*N* = 1), and a lineage 7 (L7) isolate (*N* = 1). There were 3 multi-drug-resistant isolates (MDR), 22 mono-resistant isolates, 3 pan-susceptible isolates, 10 pre-extensively (XDR) drug-resistant isolates, and 8 isolates with other drug resistance patterns. There were 11 isolates for which drug resistance information was not available (Table 1; Fig. 1). To understand the functional distribution of genes across core and accessory genomes, a GO enrichment analysis was performed powered by the PANTHER database. The core genome reports biological processes of DNA replication, transcription, and translation, but additionally, processes regarding lipid transport/localization, closely associated fatty-acid metabolism processes entangled with ATP synthesis and oxidative respiration, enoyl-coA hydratase activity, and glutamine family amino acid biosynthetic processes (Fig. S1) (79).

**TABLE 1:** Global characteristics of the pangenome of mostly drug-resistant clinical *M. tuberculosis* complex strains by five most prevalent lineages

Characteristic	All isolates	Indo-Oceanic (L1)	East Asian (L2)	East African-Indian (L3)	Euro-American (L4)	African 1 (L5)
Number of isolates in analysis, *N* (%)	110 (100%)	20 (18%)	37 (33%)	10 (9%)	38 (34%)	3 (3%)
Total pangenome/mean genome size	4,325/4,022	4,133/4,017^*b*^	4,105/4,027^*b*^	4,099/4,041^*b*^	4,209/4,016^*b*^	4,051/4,023^*b*^
Genes in core genome	3,767 (87%)	3,767 (91%)	3,767 (92%)	3,767 (92%)	3,767 (90%)	3,767 (93%)
Genes in accessory genome	558 (13%)	366 (9%)	338 (8%)	332 (8%)	442 (10%)	284 (7%)
Genes in lineage core genome	NA^*d*^	3,886 (94%)	3,915 (95%)	3,954 (97%)	3,678 (87%)	3,992 (98%)
Genes in lineage accessory genome	NA	247 (6%)	190 (5%)	145 (3%)	531 (13%)	59 (2%)
Unnamed *ab initio* predicted genes	290 (7%)	222 (5%)	205 (5%)	200 (5%)	219 (5%)	191 (5%)
Essential genes,^*a*^ *N*/total *N* (%)	715/3,766 (20.3%)	737/3,578 (20.6%)	742/3,567 (20.8%)	731/3,531 (20.7%)	730/3,529 (20.7%)	725/3,510 (20.7%)
Number of PPE genes,^*c*^ median (min-max)	68 (61–73)	65 (61–68)	71 (65–73)	67.5 (62–69)	68 (63–73)	68 (68–68)
Number of PE genes,^*c*^ median (min-max)	33 (30–33)	33 (30–33)	33 (31–33)	32 (30–32)	32 (30–33)	33 (33–33)
Number of PE_PGRS genes,^*c*^ median (min-max)	58 (54–63)	55.5 (52–56)	57 (54–61)	58 (57–60)	60 (55–63)	59 (58–60)
Number of ESX genes,^*c*^ median (min-max)	23 (20–31)	22 (20–24)	23 (21–25)	23 (21–23)	24 (21–31)	22 (22–22)
MDR	3	1	0	1	1	0
Mono-resistant	22	11	2	1	7	0
Other	8	0	2	1	5	0
Pan-susceptible	3	0	0	0	3	0
Pre-XDR	10	3	3	0	4	0
XDR	53	2	30	4	17	0
Missing	11	3	0	3	1	3

^*a*^ Essential defined as “ES,” “ED,” or “GA.” Not essential defined as “GD,” “NE,” or “Uncertain” from DeJesus et al. (80). Denominators for essential genes represent lineage-specific pangenomes with genes removed for missing essentiality information.

^*b*^ *P*-value < 0.001 using one-way Welch’s ANOVA and Games-Howell test for multiple pairwise comparisons. Drug susceptibility testing was produced using previously described methods (81, 82).

^*c*^ The number of genes is the sum of open reading frames for all locus tags identified by Panaroo as being a part of that gene family, including duplicate gene copies and merged annotations.

^*d*^ NA, not applicable.

When stratifying by lineage-specific pangenomes, the total number of *ppe*, *pe_pgrs*, and *esx* family genes did not differ greatly and were similar in count and composition compared to other studies, though slightly attenuated for PGRS family genes (Table 1; Table S1). L4 core genome contained only 3,678 (87%) genes, representing the smallest core genome among the first five lineages (other lineages: 94%–98%). However, L4 also had the most lineage-specific genes (92 genes) not shared with other lineages (Fig. 1A; Data Set S2). Although there were more L4 isolates than other lineages in our sample, the diversity seen in our L4 pangenome is consistent with what has previously been noted elsewhere (83, 84). It is important to note though that after removing H37Rv from the L4 lineage-specific pangenome analysis, the number of genes shared across all L4 isolates increased from 3,678 to 3,826 genes (91% shared).

Panaroo also identified 290 genes that were predicted *ab initio* as an open reading frame but not assigned a name by Prokka and labeled by Panaroo with a numeric “group” name (e.g., group_123). Approximately 96% (279/290) of these unnamed, *ab initio* predicted genes (hereby referred to as putative novel genes) were classified as hypothetical proteins by Prokka (Table S2). These putative novel genes consistently comprised about 5% of the lineage-specific pangenomes and nearly half of these genes were conserved in the overall core genome of all isolates (*N =* 130, 44.8%; Table 1).

### Clustering and principal component analysis of gene presence profiles reveal putative novel genes to be significant distinguishing factors between lineages

Principal component analysis (PCA) and cluster analyses were collectively used to evaluate variation in genomic composition between isolates and assess whether the genomic structure of presence/absence clustered by lineage. We also sought to identify genes whose presence or absence may be driving gene-level differences between isolates. Results from the PCA bi-plot of the first two components illustrated a distinct clustering of L2 and L1 isolates, where L2 isolates more strongly correlated with the second principal component (Fig. 1B). By contrast, L3, L4, and L5 were less distinctly clustered.

The combination of genes accounting for the most variance in the first axis (principal component 1) was *Rv0071-73* and *Rv2817c-Rv2816c* (Pearson *r*^2^ = 0.87–0.89, *P*-value < 0.0001; Fig. S2A). This is likely related to the fact that L2 isolates are known to have a deletion of the region of difference (RD) 105 that results in a gene fusion of *Rv0071* and *Rv0074*, and thus are a distinguishing factor in the PCA (85). Genes most significantly correlated with the second principal component were *tuf* (*Rv0685*)*, ppe34* (*Rv1917c*), and putative novel genes group_73, group_326, and group_233 (Pearson *r*^2^ = 0.45–0.49, *P*-value < 0.0001, Fig. S2A).

The genes that constituted the top 5% of the PCA loadings (*N* = 67 genes) for the first five principal components were largely duplicated and putative novel genes (*N =* 31/67 genes, 46%, Fig. S2B). Given their small numbers in the pangenome, we hypothesized that duplicated and putative novel genes were enriched in the top PCA loadings, and thus important for explaining the most variance between the components. To test for over-enrichment, we conducted a randomized permutation test without replacement to assess the baseline proportion of putative novel and duplicated genes we would expect to see if randomly sampling the same number of genes across the pangenome. In all the permutations (*N =* 10,000), the expected proportion of putative novel genes randomly selected ranged from 0% to 19% and 0% to 14% for duplicates; there was not one randomized sample that matched or exceeded the proportion of putative novel or duplicated genes observed in our top 5% loadings (observed proportion = 23.88% for putative novel genes, 22.39% for duplicated genes) (Fig. S3). These findings highlight the presence of these putative novel and duplicated genes as influential in differentiating the principal components, and thus their presence may be important to distinguishing lineages (Fig. S3A and B).

An unsupervised hierarchical cluster analysis based on binary presence/absence of genes supported the findings of the principal component analysis and revealed most of the pangenome to be conserved among our isolates (Fig. 1C and D). Gene presence patterns largely clustered isolates by lineage, though H37Rv singularly did not cluster with any other lineage or isolate. This clustering pattern could reflect diverging evolution between H37Rv and the more recent clinical MTBC strains from the last common ancestor or could be influenced by the fact that H37Rv alone did not have any putative novel genes reported by Panaroo in its genome.

### Genes important to *in vivo* survival are conserved in the core, though many are regarded as “non-essential”

As we would expect genes with essential function to be conserved in all isolates, we assessed the distribution of essential genes across the core and accessory genomes using definitions derived from DeJesus et al. (80) and Zhang et al. (86) *in vitro* transposon mutagenesis (TnSeq) studies, as well as Bosch et al.’s CRISPR interfering (CRISPRi) study (87). Essential genes as defined by these studies reflected very similar distributions across the overall core and accessory genomes. Among the genes with no essentiality data missing from TnSeq studies, essential genes have over four times increased odds of being in the core compared to non-essential genes (DeJesus et al.: 4.41, 95% CI: 2.6–8.4, FDR corrected *P*-value = 2.79 × 10^−6^; Zhang et al.: 4.26, 95% CI: 2.4–8.3, FDR-corrected *P*-value = 1.06 × 10^−5^, Table 2). The percent of essential genes present in the lineage-specific pangenomes did not significantly differ by lineage (Table 1). Compared to CRISPRi experiments, odds ratios were slightly attenuated, with the odds of essential genes in the core being similar between H37Rv and a lineage 2 hypervirulent strain HN878 (H37Rv: 2.29, 95% CI: 1.61–3.37; HN878: 2.19, 95% CI: 1.54–3.23).

**TABLE 2:** Univariate and bivariate analyses of essential genes and gene function categories by core vs. accessory genome

	No. genes	% pan-genome	Core *N* (%)	Access. *N* (%)	Crude OR (95% CI)	*P*-value	FDR-adjust. *P*-value
Essentiality studies							
DeJesus et al.^*a*^							
Essential gene	450	12%	439 (97.6%)	11 (2.4%)	4.41 (2.57–8.38)^*e*^	6.85e−07^*g*^	2.79e−06^*g*^
Essential domain	26	1%	25 (96.2%)	1 (3.8%)	NA^*i*^	NA	NA
Growth advantage	287	8%	251 (87.5%)	36 (12.5%)	0.80 (0.56–1.17)	0.226	0.329
Growth defect	130	3%	124 (95.4%)	6 (4.6%)	2.36 (1.12–6.06)	0.042^*f*^	0.521
Not essential	2,802	74%	2,515 (89.8%)	286 (10.2%)	Ref	NA	NA
Uncertain	71	2%	54 (76.1%)	17 (23.9%)	0.36 (0.21–0.65)	3.71e−04^*g*^	9.89e−04^*g*^
Zhang et al.^*b*^							
Essential domain	415	11%	404 (97.3%)	11 (2.7%)	4.26 (2.43–8.34)	3.32e−06^*g*^	1.06e−05^*g*^
Not essential domain	2,887	77%	2,587 (89.6%)	300 (10.4%)	Ref	NA	NA
Both regions	294	8%	269 (91.5%)	25 (8.5%)	1.25 (0.83–1.96)	0.309	0.412
Unable to determine	143	4%	123 (86.0%)	20 (14%)	0.71 (0.45–1.19)	0.174	0.278
Bosch et al.— H37Rv^*c*^							
Essential	677	18%	644 (95.1%)	33 (4.9%)	2.29 (1.61–3.37)	1.00e−05^*g*^	9.03e−05^*g*^
Not essential	3,062	82%	2,740 (89.5%)	322 (10.5%)	Ref	NA	NA
Bosch et al.—HN878^*d*^							
Essential	654	18%	621 (95.0%)	33 (5.0%)	2.19 (1.54–3.23)	2.95e−05^*g*^	2.66e−04^*g*^
Not essential	3,084	83%	2,762 (89.6%)	322 (10.4%)	Ref	NA	NA
Gene set categories^*h*^							
*Mycobacterium* spp. orthologs	1,006	26%	985 (97.9%)	21 (2.1%)	6.64 (4.25–10.94)	7.40e−27^*g*^	1.18e−25^*g*^
In DosR regulon	47	1%	43 (91.5%)	4 (8.5%)	1.15 (0.42–4.5)	1	1
In enduring hypoxic response regulon	208	5%	191 (91.8%)	17 (8.2%)	1.22 (0.73–2.16)	0.5463	0.583
Genes whose disruption alters drug efficacy (mice)	146	4%	142 (97.3%)	4 (2.7%)	3.93 (1.49–14.72)	0.002^*g*^	0.004^*g*^
Genes with methylated promoters	186	5%	176 (94.6%)	10 (5.4%)	1.94 (1.02–4.15)	0.0415^*f*^	0.083
Deletion mutants underrepresented in mouse model	527	14%	505 (95.8%)	22 (4.2%)	2.71 (1.74–4.43)	6.97e−07^*g*^	2.79e−06^*g*^
Toxins/antitoxins	147	4%	136 (92.5%)	11 (7.5%)	1.34 (0.72–2.78)	0.4758	0.544
PE/PPE genes	149	4%	113 (75.8%)	36 (24.2%)	0.31 (0.21–0.48)	1.07e-07^*g*^	8.59e-07^*g*^
Transcription factors	199	5%	188 (94.5%)	11 (5.5%)	1.88 (1.02–3.87)	0.04773^*f*^	0.085

^*a*^ *N =* 3,766 (genes with missing essentiality data that belong to the core = 57 [80%]; belong to the accessory *=* 14 [20%]).

^*b*^ *N =* 3,739 (genes with missing essentiality data that belong to the core = 82 [84%]; belong to the accessory *=* 16 [16%]).

^*c*^ *N* = 3,837 (genes with missing essentiality data that belong to the core = 81 [83%]; belong to the accessory *=* 17 [17%]).

^*d*^ *N =* 3,837 (genes with missing essentiality data that belong to the core = 82 [83%]; belong to the accessory *=* 17 [17%]).

^*e*^ “Essential domain” included with the essential gene category due to low cell count.

^*f*^ *P*-value < 0.05.

^*g*^ *P*-value < 0.001.

^*h*^ Genes from functional sets in core and accessory are not mutually exclusive (i.e., some genes may have duplicate copies in the core and accessory).

^*i*^ NA, not applicable.

While the core was significantly enriched with essential genes, there were many conserved genes that were regarded as “not essential” by both transposon mutagenesis (*N =* 2,515*,* 74% core genome) and CRISPRi studies (*N =* 2,740*,* 82% core genome). These genes may not be essential *in vitro* but may provide important functions to bacterial survival *in vivo* under certain conditions. To this end, we assessed whether the core genome was enriched with known functions important to the survival, evolution, and pathogenicity of MTBC*,* many of which have been established as significant in *in vivo* animal models. Orthologous genes shared across *Mycobacterium leprae*, *Mycobacterium smegmatis*, *Mycobacterium avium,* and *Mycobacterium abscessus* have 6.64 times the odds of being conserved in the core, highlighting genes that are likely broadly essential to *Mycobacteriu*m species (FDR corrected *P*-value = 1.18 × 10^−25^; Table 2). Of the 1,006 orthologous genes shared across these species that are present in our pangenome, 985 (97.9%) genes were recapitulated in our MTBC core genome. Other gene sets were also significantly enriched in the core genome, including genes known to limit drug efficacy, genes with MamA, MamB, or MamC DNA methylation motif sites in their promoters (88), genes whose transposon-inserted TA dinucleotide sites are underrepresented post-infection in mouse models (89), and transcription factors (TF). However, the statistical significance of core enrichment of genes with methylated promoters and TFs was lost after adjusting for the false discovery rate (FDR; Table 2). Conversely, *pe/ppe* genes had significantly reduced odds of being in the core compared to the accessory genome, highlighting their variability in number across our sample (Table 2). Nine hundred sixty-one genes from the gene sets significantly enriched in the core were labeled as “not essential” according to the *in vitro* DeJesus et al.’s study, accounting for approximately 38% (961/2515) of all the “not essential” genes in our core genome. These findings highlight genes determined as essential may be grossly underestimated from *in vitro* studies alone, as many genes’ essentiality may come from functions that do not maximize growth rate or may prove essential in a certain particular environmental context.

### ESX, PE, PE_PGRS, and PPE gene families

MTBC genomes harbor four gene families that carry clinically important roles such as virulence regulation (90–92), host-pathogen interaction (93–96), and immune evasion (47, 93, 97, 98). Unfortunately, mainly due to sequence similarity, they present significant challenges in most Whole-Genome Sequencing projects and therefore are understudied. Our use of long-read sequencing allows us to overcome these challenges. The diversity in the presence and count of these genes can attenuate or enhance the aforementioned phenotypic characteristics of an isolate, making the correct identification and characterization of these gene families’ constituents vastly important (99, 100).

One of the main contributions of this project is a complete account of all *esx*, *pe, pe_pgrs*, and *ppe* genes, including their homologs and paralogs. Here, we report 12 *esx*, 26 *pe*, 45 *pe_pgrs*, and 45 *ppe* unique genes in the MTBC core genome, excluding duplicate copies (Table S1). We report an additional seven *esx*, six *pe*, eight *pe_pgrs*, and 16 *ppe* unique genes in the MTBC accessory genome (Table S1). To avoid reporting duplicates, these counts do not include genes that are merged by Panaroo (e.g., ppe12 ~~~ppe13). If one considers counts such cases, then the core counts increase to 17 *esx*, 28 *pe*, 50 *pe_pgrs*, and 52 *ppe* total genes, while the accessory counts increase to 14 *esx*, six *pe*, 11 *pe_pgrs*, and 29 total *ppe* genes. In our genomes, we observe the greatest variability in *esx* genes, in terms of presence and absence. However, this variability was mostly driven by L4-specific diversity (Table 1). *pe* genes were conserved across our genomes. We observed a total range (including duplicate gene copies) of 20–31 *esx* genes (median 23), 61–73 *ppe* genes (median 68), 30–33 *pe* genes (median 33), and 54–63 *pe_pgrs* genes (median 68) within each genome (Table 1). For *esx* genes, L4 had marginally highest median but distinctly higher diversity than other lineages. While L2 had the highest median number of *ppe* genes among the first five lineages, L4 had a distinctly higher diversity in *ppe* gene content. The remaining lineages had a similar relatively lower *ppe* gene diversity (Table 1). In terms of the *pe_pgrs* gene content, L1 had the lowest median and L4 had the highest. In terms of diversity, perhaps unsurprisingly, the two most successful lineages L2 and L4 had a distinctly higher variability in terms of *pe_pgrs* gene counts (Table 1). Finally, the overall diversity of *esx*, *ppe*, and *pe_pgrs* genes was higher than the within-lineage diversity of each of these families, indicating the prevalence of lineage-specific genes in each of these families.

*Shannon’s entropy* index is a measure of uncertainty (101, 102) used typically to discover information content (i.e., distinguish between noise and information). This index is shown in Data Set S4 with representative sequences in Data Set S5. The minimum index is zero indicating no change in the data. It elevates above zero when change is detected; however, it remains low if the change is random. It is elevated higher when the change follows a pattern (repeated observation). In this context, we used the measure to answer the question of which gene family’s diversity (in terms of gene presence/absence) follows a conserved pattern (convergent evolution) and therefore carries the highest information content and hence is most impactful on the strains’ phenotypic diversity. As can be seen (Data Set S4), Shannon uncertainty index of *ppe* genes is the highest at 4.377, followed distantly by the *esx* family at 2.494, *pe_pgrs* family at 2.073, and *pe* family at 0.588. The *pe* family was quite stable except for *pe27A* (*Rv3018A*, deleted in 33 isolates) and *pe25* (*Rv1787*, duplicated in 90 isolates). *pe_pgrs4* (*Rv0279c*, deleted in 54 isolates), *pe_pgrs30* (*Rv1651c*, present only in 17 isolates), *pe_pgrs49-50* (*Rv3345c-Rv3344c*, deleted together in 25 isolates), *pgrs54* (*Rv3508*, deleted in 23 isolates), *pgrs57* (*Rv3508*, deleted in 82 isolates, the remaining 27 had two copies of this gene), and minimally *pgrs28* (present in all isolates but four had a duplicate copy) had the highest contribution to *pgrs* Shannon index. Among *esx* genes, *esxJ, esxL, esxN, esxO* contributed the most to the convergent diversity (each change was observed in multiple lineages) in this family, with the latter three often duplicated together. Among *ppe* genes, *ppe34* (*Rv1917c*, greatest source of diversity through frequent duplication, often multiple times) and *ppe16* (*Rv1452c*, duplicated in 28 isolates) had the largest contribution. Even though *esx* genes show greater relative range of diversity, Shannon’s entropy indicates that presence/absence of *ppe* genes carry more information and therefore are most impactful on the diversity of MTBC. None of the events reported in this section were lineage-specific and were observed convergently across multiple lineages (85).

### Putative novel genes are more prevalent in clinical isolates than in the H37Rv reference genome

Since putative novel genes were not found by Panaroo in H37Rv using the curated H37Rv GFF from NCBI as input, and thus clustered separately from other isolates, we hypothesized that identification of these putative novel genes was dependent on Prokka identifying the open reading frames. As such, we evaluated whether Prokka runs on the NCBI H37Rv-NC_000962.3 FASTA file would be able to identify the same putative novel genes present in our clinical isolates. Prokka identified 327 unnamed open reading frames in H37Rv, of which 77 open reading frames did not overlap with named gene coordinates in the curated NCBI GFF file. Alignment to our Panaroo-derived pangenome multi-FASTA file with BLASTN resulted in 79 matches. Genes with the highest query coverage, percent identity, and E-value were retained where queried sequences had aligned to multiple genes. In total, 74 open reading frames in H37Rv aligned to our pangenome’s putative novel genes, two open reading frames aligned to named genes (*Rv2807, dsx*), and one open reading frame did not match any genes in the pangenome. Most of these unannotated gene regions in H37Rv aligned with high percent identity (median = 100%, range = 97.87%–100%) and sequence coverage (70/74 aligned at 100% coverage). Only four sequences matched at <100% coverage (median: 70.80%, range: 24.34-87.64%). These findings support our hypothesis that many genes identified by Panaroo and missing in H37Rv were due to using the NCBI curated GFF file as input to Panaroo, and not the Prokka-produced version. These findings spotlight the areas of the H37Rv file from NCBI that are yet unannotated, distinguishing them from truly novel genes in the MTBC pangenome (i.e., genes absent from the reference genome, 216/290 genes, 74%), and denote how relying on referenced-based annotations alone will likely miss some genetic content. It is important to also note that most isolates had at least 171 putative novel genes, which comprised at least 4.25% of their genomes. Seeing that H37Rv still had a much smaller number (*N =* 74 matched) and percent (1.9% of total genome) of putative novel genes than the other isolates signifies that H37Rv lacks genetic content that is present in clinical isolates (Data Set S6).

### Characterization of putative novel genes show conserved regions of unknown function

Our principal component analysis highlighted how the presence or absence of putative novel genes may drive lineage clustering and were fairly conserved (Fig. 1; Table S2). We hypothesized that Panaroo-identified putative novel genes were likely gene fragments since they generally were short (median: 218 base pair [bp], range: 90–2,746 bp). Therefore, we aligned the Panaroo-derived representative sequences of these genes to the NCBI H37Rv genome to assess the proportion of putative novel genes that are gene duplicates, fragments, or unable to be determined. We assessed the best BLASTN hits with the highest percent identity and highest coverage (i.e., alignment length/query length) (Data Set S6). Of the 290 putative novel genes identified in the pangenome, 167 (58%) putative novel genes were able to roughly align to a named gene in H37Rv (164/167 [98%] were truly novel genes and entirely absent from the H37Rv reference genome). For 136 putative novel genes, the gene in H37Rv that aligned with the highest percent identity also had the highest query coverage where multiple BLAST hits were seen. Among these 136 putative novel genes that had concordant matches on identity and coverage, all exceeded 80% identity (median: 100%); however, the query coverage was often low (min: 1.48%, median: 23.18%, max: 102.13%, IQR: 11.20%–44.02%).

Twelve putative novel genes matched to a gene in H37Rv with high identity and coverage ≥80%, indicating that they are likely duplicates. While query coverage remained relatively high, the length of the gene in Panaroo exceeded the length of the aligned gene in H37Rv by ≥100 bp for five genes. The functions of most of the aligned genes remain unknown; however, some are hypothesized to encode fatty acid CoA ligase (*fadD18* [*Rv3513c*]) and a two-component system sensor kinase (*Rv2998A*). Two putative novel genes (group_266 and group_361) aligned with 100% identity and 92%–94% coverage to *fadD18*. Seeing that most of these putative novel genes are prevalent in our sample (*N* = 6/12 conserved in the core genome; 9/12 present in over two-thirds of our isolates), it is important to further investigate whether these genes are being expressed and what role they may play in MTBC survival.

Of the 290 putative novel genes, 123 did not align with a gene by BLASTN. Of these, 76 genes were conserved in the core genome (18/76 [24%] were truly novel genes and not present in the H37Rv reference genome), constituting roughly 58% of all core putative novel genes. The genes that did not align did not differ greatly in sequence length (median: 186 bp vs. 255 bp in aligned genes). Gene ontology analysis of aligned genes revealed that general cellular processes were underrepresented (0.52-fold enrichment, FDR adjusted *P*-value = 0.023), and over-enriched for unclassified molecular function (1.57-fold enrichment, FDR adjusted *P*-value = 0.038), especially among aligned genes that are in the core pangenome (1.73-fold enrichment, FDR adjusted *P*-value = 0.018). These findings collectively suggest that while few putative novel genes may be duplicates, most are mutated fragments of genes of unknown function. As previous studies have shown that translation of mutated and truncated genes often retains some function (103), sometimes with clinically relevant changes (104), further research investigation of these mutated fragments’ function and role during infection is warranted, prioritizing those in the core genome.

Many putative novel genes were found in only one lineage (82/290, 28.3%). Only one putative novel gene was seen in all L3 isolates, one gene was in all L4 isolates (except for H37Rv), two genes were in all L5 isolates, and three genes were only seen in our L6 isolate (Data Set S6). The putative novel genes found only in L5 and L6 matched an annotated gene present in H37Rv with high percent identity (>99%) but low coverage (<25%), including a putative novel gene with high percent identity to a portion of IS6110 at a locus in the pangenome unique to all L5 isolates. More research with expanded data sets is needed to confirm the use of these regions as potential lineage markers.

### Gene duplications of drug-resistant and virulence genes are common and may aid drug resistance

To further explore mechanisms of gene gain, we assessed how many genes were duplicated across the pangenome. A total of 86 genes were duplicated in the pangenome of all 110 isolates, adding 144 extra gene copies, constituting approximately 5% of the pangenome (Table 3). Genes with the highest average copy numbers included IS6110, IS1081, and *esxJ* (*Rv1038c*) (Table 4). However, some of the copies of *esxJ* were mis-annotations of *esxK* (*Rv1197*) since the two are nearly identical (six SNPs different) and the same length. While Panaroo corrects for most gene mis-annotation, Panaroo might not distinguish closely homologous genes well. The persistent challenge to correctly annotate these genes highlights the need for better annotation tools.

**TABLE 3:** Distribution and frequency of duplicate genes in core and accessory genomes

Duplicated gene category	Number of genes
Total duplicated (% pangenome)	86 unique/230 total (5%)
Total core (% pangenome)	83 (36%)
Total accessory (% pangenome)	147 (64%)
All copies in the core	10 unique/20 total
All copies in the accessory	20 unique/70 total
Copies in core and accessory	56 unique/140 total

**TABLE 4:** Average copy number of commonly duplicated genes

Commonly duplicated genes	Average copy no.
*ppe59*; *ppe57*	1.83
*pe1*	1.98
*Rv0036/Rv0515*	2
*mgtE*	2
*tuf*	1.57
*Rv0795* (IS6110 (1))	13.33
*Rv0796* (IS6110 (2))	15.11
*sseC1/2*	2
*cysA2/3*	2
*esxJ*	3.28
*Rv1042c/Rv1149*	1.99
*Rv1047* (IS1081)	4.19
*embR*	1.87
*Rv1313c* (IS1557)	2
*ppe34*	1.61
*Rv2082*	2.13
*ppe38*	1.56
*pe25*	1.82
*Rv2807*	1.99
*Rv3348* (IS1608)	1.96
*amiD*	1.99
*espI*	1.99

**Fig 2:**
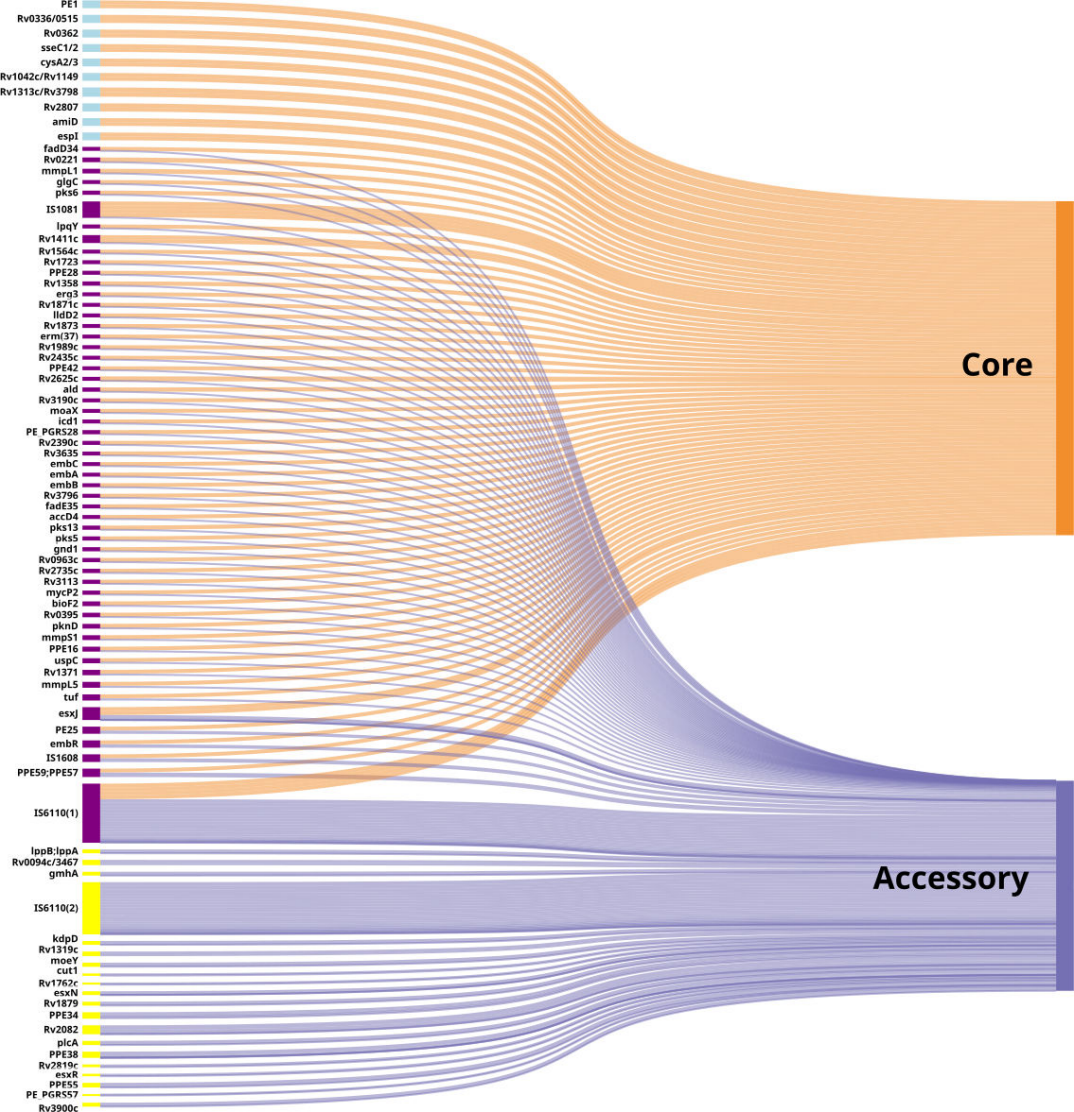
Sankey diagram showing the distribution of genes with duplicate copies in the pangenome by the number of sequences in the core and/or accessory pangenome. The thickness of the line indicates a greater number of sequences. Sankey diagram made on SankeyMATIC (105).

Most duplicated genes (56/86 genes) had a single gene copy (i.e., core copy) and less than 99% of isolates had duplicate copy/copies (i.e., accessory copy). Ten duplicated genes had all copies in the core, while 20 genes had all copies in the accessory (Fig. 2; Table 3). Core duplications comprised transposases (2/10), and sulfur metabolism (2/10), among other functions (amidase, ESX-1 secretion protein, magnesium transport protein). Duplicated genes in the accessory were not enriched for any particular function. Most duplicates were not tandem but duplicated elsewhere in the pangenome; however, six genes also had tandem duplicates in the pangenome neighborhood structure (IS1081*,* IS6110, *pe_pgrs57, esxJ*, and *cysA2* [*Rv0815c*]).

For lineage-specific pangenomes, duplicated genes were considered “core” if all isolates in the lineage had the duplication, and accessory if the duplication was in at least one but less than all isolates in the lineage. L4 had the fewest duplications where all gene copies were in the lineage-specific core genome, whereas L1 had the most genes with all copies in its core (Fig. S4; Table S4). The median number of duplicates differed between lineages (Kruskal Wallis, *P*-value = 0.0064), and were more frequent in L3 than in L4 (Fig. S5, *P* = 0.014). While some genes were consistently duplicated across all lineages, the number of duplicate copies and genes with the duplication varied across lineages. There were variable numbers of copies across lineages for IS element genes (IS1081 and IS6110) and *esxJ* (Fig. S5). L6 and 7 had fewer copies of IS6110 genes compared to other lineages, but not for IS1081. In general, modern lineages (L2, L3, and L4) had more IS element copies and more duplications among *pe* and *ppe* genes. However, ancient (L1 and L5) and intermediate lineages (L6 and L7) have more prevalent duplications of MmpL family genes than modern lineages (Fig. S5).

A few of the duplicated genes in the pangenome are known to be associated with drug resistance, such as *mmpL5* (*Rv0676c*) and *embC-A* (*Rv3793-Rv3795*), affiliated with bedaquiline and ethambutol resistance, respectively. Duplications of *mmpL5* and *mmpS1* (*Rv0403c*) were observed in all L1 isolates and thus considered core for that lineage. Only two of these isolates had a fragmented copy of *mmpL5*. Upon further investigation, while the amino acid sequence of the *mmpL5* duplicate only had 32% identity to the copy in H37Rv, the E-value was very significant (8.34e−159), had a similar length (945 vs. 964 amino acids), and was predicted by AlphaFold (106, 107) to produce a MmpL5-like protein. Similarly, some genes in the *embC-A* operon had duplicate copies in isolates across all lineages. While duplication of *embR* (*Rv1267c*) was very prevalent (*N =* 96 isolates), this duplication also had a low percent identified by BLASTp (57%) but significant E-value (8e−156) and protein folding prediction of an EmbR protein. Only one isolate (1-0110) had a duplication of all genes in the *embC-A* operon. This isolate was found resistant to all tested antibiotics and thus labeled as XDR. None of these gene copies were found to have any intragenic variants; however, one copy of *embC* was shortened (1,550 bp, 47% of gene in H37Rv).

### Fragmentation-causing variants specifically can lead to pseudogenization and provide a biological advantage

To characterize gene loss, we evaluated the number of potentially pseudogenized genes caused by gene fragmentation. These fragments often occur through a mutation that produces an early stop codon that putatively disrupts translation. While Prokka can annotate open reading frames in prokaryotic genomes, it does not accurately label all gene fragments as arising from the same gene. Instead, it often separates fragments as distinct unnamed open reading frames and describes them in the note field as a “hypothetical protein.” Panaroo’s gene adjacency network corrects for these labeling errors by grouping fragments that arise within the same gene locus and reporting multiple Prokka locus tags at a given locus. We assessed the genes with multiple open reading frames at a given locus to delineate the distribution of fragmented and thus potentially pseudogenized genes in the pangenome. Our pangenome analysis identified 667 genes (15.4% of pangenome) that had several Prokka-specific locus tag names in a single locus for at least one isolate. Of these 667 genes, 102 were merged annotations representing multi-gene loci, six were putative novel genes, and 559 named genes (554 unique genes); together, the 559 named genes and the 6 putative novel genes comprised 565 putative fragmented genes. Fragmented genes commonly occurred in isolation, with 294 out of 565 fragmented in only one isolate, and 495 genes fragmented in 10 or fewer isolates (Fig. S6). Except for H37Rv which did not have any fragmented genes in the NCBI version, there were an average of 26 genes fragmented in each isolate (min: 8, median: 24, max: 65, std: 9.72). L1 isolates had a significantly higher median number of fragmented genes compared to L2 (L1: 36 genes, L2: 25 genes, *P*-value = 0.0001), and L4 (L4: 17.5 genes, *P*-value = 7.7e−13) (Fig. S7). While PacBio SMRT sequencing does have increased indel error rates, the error rate at the consensus level has been reported as low (108). Therefore, although we cannot definitively rule out some fragmentation as a result of sequencing error, it is reasonable to conclude that these fragments cannot be solely attributed to sequencing errors, but mostly reflect genes truly fragmented in these genomes.

We further investigated genes most frequently fragmented to assess if mutations causing fragmentation could provide biological advantages or simply reflect genes individually undergoing negative selection. No genes were fragmented in all isolates; however, a few genes were frequently fragmented, including *Rv0061c* (66 isolates), *Rv0134* (*ephF,* 56 isolates), and *Rv0797* (IS1547, 51 isolates). These top three most commonly fragmented genes were all fragmented by nonsense mutations, though *ephF* also had a mixture of nonsense variants and indels causing frameshifts. None of these genes had any duplications to recover function. *Rv0061c* had a nonsense mutation at nucleotide position 139 in 36 isolates (*Rv0061c*_c139t), which constituted all but one of our sample’s L2 isolates. The nonsense mutation within *Rv0061c* on the complement strand occurs 402 base pairs upstream of the start of *Rv0062* (*celA1*) (*celA1*_g-402a) on the positive strand, a gene encoding for cellulase that may bear significance for biofilm maintenance and drug tolerance (109). To assess whether this mutation disrupted the promoter region of *celA1*, we utilized the motif-variant probe to identify if sigma factor binding motifs for *celA1* were disrupted by this variant (110). We found that the *celA1*_g-402a variant caused the creation of a SigI_10 sigma factor motif (G[AGT][AGCT][GCA][TAG][CA], or GDNVDM in terms of IUPAC nucleotide ambiguity codes). Extra-cytoplasmic function (ECF) sigma factors like SigI are important for response to adverse environmental stressors (111, 112). The creation of a SigI binding motif preceding *celA1* may allow rapid regulation of biofilm, thus aiding its survival.

### Structural variants contribute to genetic diversity in conjunction with single-gene events

**Fig 3:**
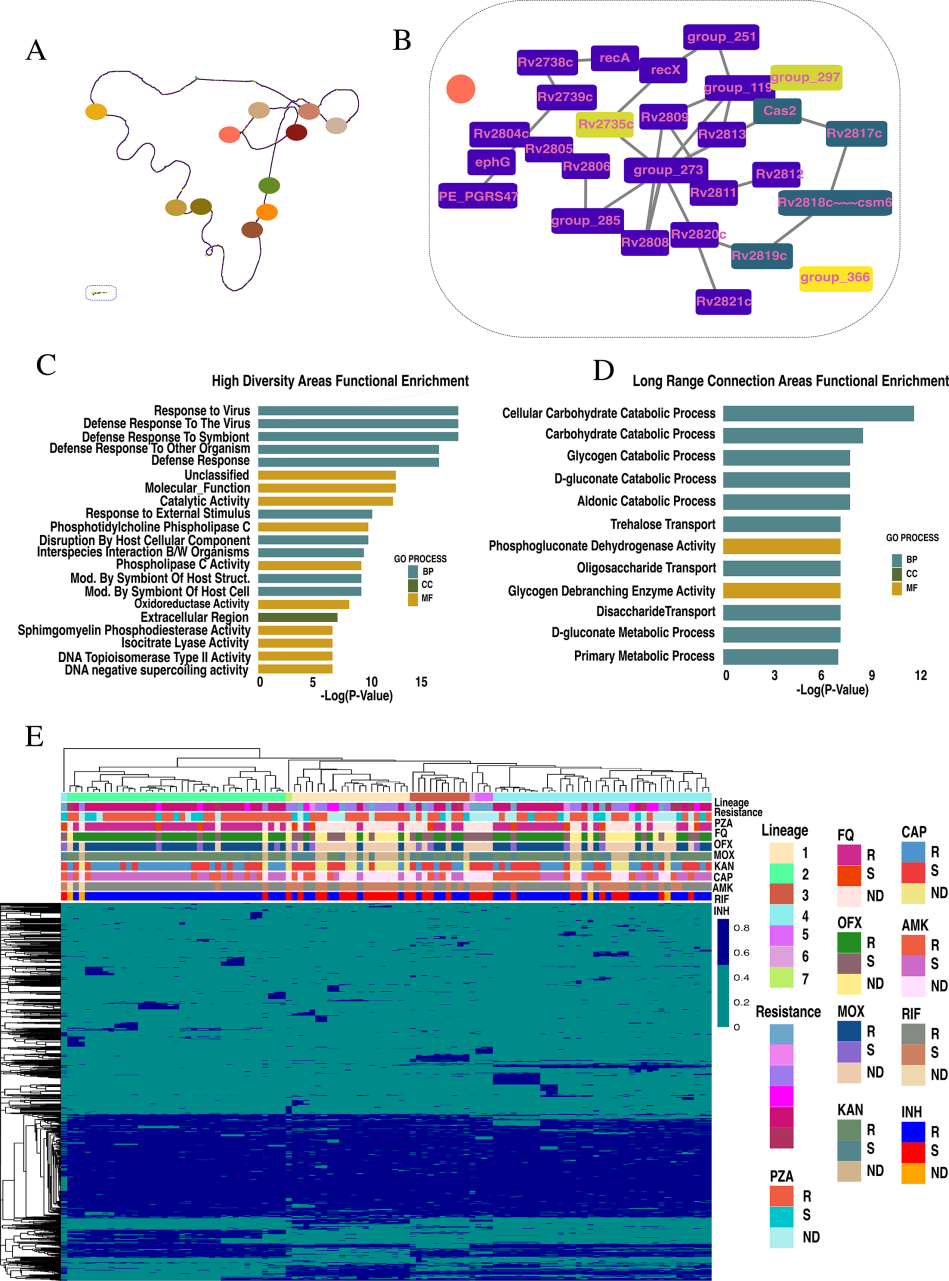
(**A**) Topological overview of gene adjacency graph of *N =* 110 isolates in this pangenome analysis. (**B**) Gene-adjacency network of highly structurally variable region containing *cas2* gene-containing variants. (**C**) GO enrichment analysis of highly variable genomic regions found through orthogonal network analysis. (**D**) GO enrichment analysis of long-range connections found through orthogonal network analysis. (**E**) Hierarchical clustering of Panaroo determined structural variants by Jaccard Index on presence-absence data. Annotation of drug resistance for pza, fq, ofx, mox, kan, cap, amk, rif, inh, lineage, and WHO classified drug resistance profile. (**E**) Counts of isolates according to antibiotic resistance (resistant, susceptible, or not determined).

Structural variations (SVs) (insertions, deletions, transpositions, and variable-tract duplications) can be studied using gene-adjacency information in network analysis. The gene adjacency graph of the MTBC pangenome demonstrates resolved adjacency minus some regions (Fig. 3A). Panaroo found 1,935 SVs among the 110 isolates (Fig. 3D) distributed along the walk of the network with uniform genomic concentration. Short SVs (five or fewer genes) output by Panaroo were hierarchically clustered for global patterns in their distribution among isolates. This revealed no clear pattern between SV presence/absence and drug resistance for PZA, RIF, INH, or any fluoroquinolone or injectable drugs (Fig. 3E). SVs organized according to lineage, except for H37Rv, which clustered apart from L4 isolates. The discordant clustering of H37Rv with lineage is likely explained by SVs accumulated during its prolonged serial passaging and *in vitro* evolution. The median number of structural variants in an isolate was 676, with the max number (*N* = 702) contained by an L2 isolate.

Single genes contributing to genetic diversity through aggregate formations were extracted from the SVs, and a dictionary of contributions was created. Of the 4,325 genes identified by Panaroo, 1,457 were involved in an SV across the pangenome. These genes contributed to an average of two SVs, but 687 genes contributed to up to 44 SVs. Many SV contributors were duplicated, namely *Rv3799c* (*accD4*)*, Rv1872c* (*lldD2*)*, Rv2608* (*ppe42), Rv2352c* (*ppe38*)*, Rv0035* (*fadD34*)*, Rv1989c, Rv2780* (*ald*)*, Rv2819c, Rv1873, Rv2625c, Rv1879, Rv1844c* (*gnd1*)*, Rv0221, Rv3190c, esxJ, Rv2318* (*uspC*)*, Rv1213* (*glgC*)*, Rv0113* (*gmhA*)*, Rv2390c, Rv0402c* (*mmpL1*)*, Rv2351c* (*plcA*)*, embR, Rv0795* (IS6110)*, mmpS1, ppe34,* and *mmpL5*. Interestingly, the top 20 gene contributors to SVs were putative novel genes (each contributing between 44 and 30 SVs), followed by *ppe39* (*Rv2351c,* 30 SVs) and an IS6110 element (29 SVs).

### Top contributors to structural variations associated with lineage and drug resistance include many putative novel genes and genes with merged annotations

An SV-genome-wide association study was performed to better capture the association between drug resistance phenotype and Panaroo-detected SVs. The model found SVs associated with drug resistance phenotype, but these results were nonsignificant after multiple hypothesis testing corrections (Table S4). The intersect of top hits before multiple hypothesis testing corrections, however, found SVs containing 96 putative novel genes (“group” genes) as well as 12 *ppe* genes, IS6110, 6 *esx* genes, 6 *pe_pgrs* genes, *Rv1266c* (*pknH*)*,* and several *plc* genes (Table S5), supporting their importance not as singular genes, rather SVs in association with drug resistance. Eighty-seven genes were found in multiple SVs associated with drug resistance to any antibiotic, with the top prevalent genes including group_303*, Rv2348c,* group_217*,* the *esxN~~~esxO~~~esxL* merged annotation, the *lppA~~~vapB18~~~lppB* merged annotation and group_340.

The presence of the *esxN~~~esxO~~~esxL* merged annotation was interesting as it was not the separate genes called as significant in many drug-resistant SVs, but rather the multiple genes identified at that locus across genomes. This is a “merged annotation” with variable genetic composition among isolates. Figure S8 illustrates that these merged annotations (containing a tilde between gene names, e.g., geneA~~~geneB) were interchangeable at a given locus in the pangenome, different genes that were interchangeable with an added duplicate copy of one of the genes, multiple annotated genes present at the same locus, or an unnamed gene annotated as “*ab initio* prediction” by Prokka. L1 (*N =* 18), L2 (*N =* 353), L3 (*N =* 75), and L4 (*N =* 335, *N =* 320 when excluding H37Rv) contained SVs specific to the respective lineage (Fig. S9 and S10). L1 had 18 lineage-specific SVs, L2 had 353, and L3 included 75 variants (Tables S6 through S10). L4-specific variants (not including H37Rv) totaled 320 variants, adding H37Rv provided 15 additional variants (Tables S9 and 10).

### Systematic network analysis identifies larger structural variants than those reported by Panaroo

Complementary to Panaroo output SVs, a systematic network analysis was conducted to identify larger SVs. The gene-adjacency network (Fig. 3A) was not well interconnected as interpreted by the clustering coefficient (0.1 ± 0.2), betweenness centrality (0.0 ± 0.01), neighborhood connectivity (2.7 ± 1.9), and topological coefficient (0.5 ± 0.1) all being low (the value resting between 0 and 1; Fig. S11). Along the network, there are a total of 11 gene neighborhoods with high complexity in gene adjacency uncharacterizable by known network structures and contain an overrepresented number of nodes (2–3 std. deviations) (Fisher’s exact test, *P*-value < 0.0001; Table S12; Fig. 3A and E). These regions house *pe*, *ppe*, and *pe_pgrs* genes, sites of IS transposon insertion, and consistently include many putative novel genes. GO term enrichment analysis of the high SV network regions identified enrichment in defense responses to virus or other organisms, interspecies interaction between organisms, modification by symbiont of host structure, molecular functions such as phosphatidylcholine phospholipase C activity, oxidoreductase activity, isocitrate lyase activity, and cellular component activity in the extracellular region (Fig. 3C).

In all, 191 genes contributing to Panaroo SVs were also found in this analysis. Many overlaps contributed to lower numbers of SVs, which is paradoxical to the high-order count and centrality of the genes/nodes in high SV regions. Five of the genes contributing to the most SVs were found in high structurally variable regions: group_122, group_290, *ppe39,* group_110, and group_134, contributing to 43, 37, 30, 32, and 42 variants, respectively.

When the backbone of the gene-adjacency network was resolved to an isolate dissimilar to the isolates included here (SEA11278), a total of 1,130 edges were cut, compared to the 4,057 that were conserved in the network. These long-range connections were through 69 nodes including IS element transposons, *pe*/*pe_pgrs*/*pe* genes, putative novel genes, and some ESX secretion system-associated genes. GO enrichment analysis rendered catabolic processes for cellular carbohydrate, glycogen, D-gluconate, aldonic acid, enzymatic activity for compounds such as phosphogluconate dehydrogenase and glycogen debranching, and some transport activity for trehalose, disaccharides, and oligosaccharides (Fig. 3D). The enrichment of these pathways was largely supported by the presence of genes *gnd1, Rv1564c* (*glgX*)*, Rv1140, Rv2058c* (*rpmB2*)*, Rv1358, Rv3327,* and Rv1527c (*pks5*).

The number of SVs found in these reference-quality genomes showcases the ability of these polished genomes to identify variations in gene adjacency across all contigs which have formed a resolved, contiguous genome. The genes that contribute to a low number of SVs are tributaries to low-level genetic diversity, complemented by those that contribute to a higher number of SVs.

### Pangenome analysis indicates MTBC has a slightly open pangenome

**Fig 4:**
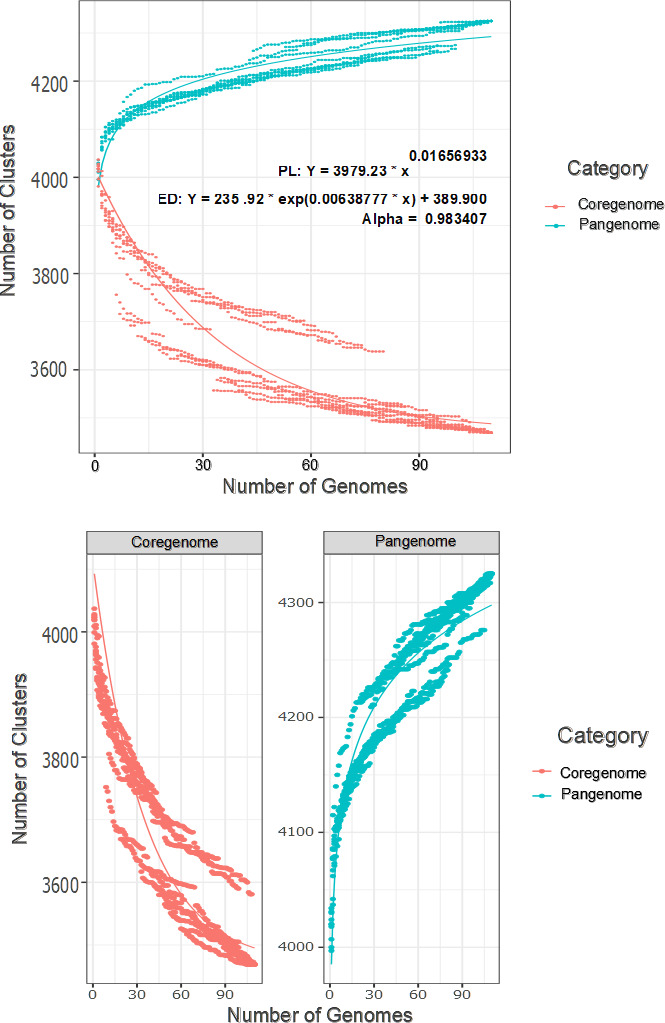
Fitted power law (PL) and exponential decay (ED) curve for the pangenome rarefaction simulation. The attribute Heaps alpha < 1 indicates an open pangenome. Therefore, analysis of our sample of mostly drug-resistant isolates indicates *M. tuberculosis* complex pangenome is expanding at a very slow rate.

A comprehensive analysis of the pangenome was conducted to assess whether our MTBC isolates produce an open or closed pangenome. We conducted pangenome and core genome rarefaction simulations to fit the power law and exponential decay curves that comprise the pangenome curve (113). Figure 4 displays the change in gene clusters by step-wise random addition of genomes. The resulting Heaps alpha metric of less than 1 (0.983) indicates an open pangenome (114), which is supported elsewhere (29). This indicates that MTBC has an expanding genome, where increases in accessory genes are seen with the addition of more isolates. The alpha metric in our study is close to one, suggesting that the MTBC pangenome increases at a very slow rate.

## DISCUSSION

Our pangenome analysis showcases regions of the *M. tuberculosis* complex genome that exhibit the greatest genomic diversity across lineages. This pangenome was constructed from a large set of polished, reference-quality *M. tuberculosis* complex genomes, and was bolstered by two distinct forms of network analysis, supporting the robustness of identified structural variants implicated in drug resistance and virulence. Although most of the pangenome comprised highly conserved genes, genomic and network analyses showed genetic diversity through duplication events, gene fragmentation, creation of putative novel genes, and structural variation which could lead to changes in cellular phenotype (68, 115). Our findings also spotlight the need for more research into gene essentiality, as defined by H37Rv genes required for *in vitro* survival. Our finding that numerous *in vitro* non-essential genes are in the core genome, 38% of which provide functions known to be important for *in vivo* survival, suggests that the current definition of essentiality may underestimate and miss genes that have essential roles during infection or transmission within the host. More research is needed to clarify which genes are essential within these important *in vivo* contexts in humans, including genes that are not present in H37Rv.

The core genome falls between other reported core pangenome sizes, which range from 1,176 to 4,391 genes in sample populations comprised of 21 to >3,400 isolates. Other pangenome studies’ foci were distinct from ours and included pangenomes among animal-host *Mycobacterium* strains (66), lineage-specific pangenomes (29), the pangenome among modern and ancient lineages (55), and a pangenomes solely among *M. africanum* strains(Data Set S1) (30, 45, 57, 116). These pangenomes contribute a composite image of the state of genetic diversity among important MTBC populations and affirm many of the findings here including gene loss or duplication as a driver of virulence, host-tissue colonization, and the importance of metabolism in bacterial survival (particularly lipid metabolism). Our graph-based pangenome analysis of our in-house *de novo* assembled, long-read sequenced, reference-quality isolates captured a core genome size comparable to studies with much greater sample sizes, potentially due to improved annotation correction and capture of genes that previously were excluded due to shortcomings of short-read sequencing which results in annotation or alignment difficulties (54, 56); Table S1).

Our study found similar counts and composition, but higher diversity of *ppe*, *pe*/*pe_pgrs,* and *esx* family genes as have been described elsewhere (93). These genes are often excluded from studies due to their high sequence homology (35), frequent gene duplication and conversion, and large repetitive regions, which each complicate assembly and annotation (56, 117–120). Although *esx* genes carried the greatest relative diversity, the diversity of *ppe* genes carries more information. The greatest contributor to the diversity (among all genes) was *ppe34* which was frequently duplicated (often multiple times). *ppe34* is known to be highly polymorphic, and a hotspot for recombination IS6110 insertion hotspot (121). These duplications likely impact phenotype; *ppe34* is a cell wall protein that modulates host immunity: *ppe34*-induced T_h_2 cellular immunity is associated with increased expression of cyclooxygenase-2 (*cox-2*) in dendritic cells (122). As such, *ppe34* duplication enhances the cell’s ability to induce *cox-2* expression in dendritic cells. Since *esx* genes secrete PPE proteins (123), it appears that *ppe* genes’ diversity may be used in targeted responses (e.g., *cox-2* expression modulation) to specific host/drug pressures while *esx* diversity is employed more for broad-scale environmental responses. As *pe/pe_pgrs*/*ppe* family genes play an important role in host immune interactions (93–95, 124), specifically through immune evasion (47, 97, 125) from antigenic variation caused by SVs, and are the target of several vaccine candidates (126–132), these findings could impact the research of pathogen therapeutic targets for vaccine development. Due to this genomic variation, research must focus on continuing to type the diversity of these genes before they can be considered for immune therapeutic purposes against infection.

Network analyses showcase other genes important for interaction with the host immune system and highlight areas of further research. The highly variable network regions which stemmed off the shared backbone (i.e., omnipresent and structurally conserved genes among our isolates) contained a GO enrichment for defense against phages/viruses, reported through overrepresentation of *Rv2816c* (*cas2*)*, Rv2817c* (*cas1*)*, Rv2818c* (*csm6*)*, Rv2819c* (*csm5*)*, Rv2820c* (*csm4*)*,* and *Rv2821c* (*csm3*). *cas2, cas1,* and *csm6* are predicted to be in an operon, as are *csm5, csm4,* and *csm3. cas1* and *cas2* participate in a highly variable network region (breaking the operon structure) identified through systematic network analysis. *cas2* is reported in several structural variants implicated in fluoroquinolone (*cas1-cas2*-group_117) and moxifloxacin (*cas1-cas2-*group194) resistance in the Pyseer model executed. These genes center on a secretion system and induce IL-6, IL-1β, and TNF-α release in macrophages altering host immunity (133). These highly variable regions in the network also contain phospholipase C activity via the *plc* family of genes (corroborated through our orthogonal analyses), which induce apoptosis in macrophages. Any isolates with these genes (and sometimes their duplicates) could experience greater resistance to macrophage microbiocidal activity (134). More research needs to be done to elucidate the nature of the association between many SVs in gene order and drug resistance. Also, more investigation is needed to assess whether variations in gene order and copy number of *plc* genes may attribute a survival advantage and aid virulence through operon restructuring, or simply reflect genomic changes that occur upon culturing *in vitro* (135). These genes have been repeatedly implicated in gene loss events and variation in clinical isolates (136, 137), substantiating the validity of our network analysis approach.

Higher-order structural variants similarly identified genes associated with host tissue colonization and immune system evasion in a few clinical isolates. The prominent trehalose transport pathway is differentially distinct among our isolates and contributes to caseous granuloma catabolism processes, macrophage lysosome-phagosome fusion, IL-12-mediated modulation of IFN-γ expression which centrally facilitates canonical granuloma formation, and the development of post-primary disease due to the high lipid content. The enrichment of lipid metabolism processes in the core genome through GO analysis highlights the centrality of functions that sustain the bacteria in-host within macrophages (48) and granuloma, the immune-cell environment that encases infection in the lung. This phenomenon as supported by these two orthogonal analyses has not been specifically named in previous pangenome analyses as an important delineating factor among isolates, despite these processes contributing to the high virulence of MTBC which can vary among isolates and even lineages. The long-range connections separated from the network backbone, signifying genes that are only in a few isolates and not structurally conserved, also show high enrichment in glycogen, glucan catabolism, and metabolism, which are important for bacterial cell wall maintenance and interaction with host cells during colonization of host tissues. Cellular carbohydrates form part of the pathogen’s envelope and promote pathogenicity and virulence due to their protective effects from the host. This allows MTBC to remain in the host for extended periods of time and contributes to persistence (138). Since MTBC’s ability to control the environment and immune system enables the pathogen to persist and infection advance, it is pivotal to evaluate whether genomes with an increased number of genes engaged in immunomodulatory processes, such as cell envelope or trehalose production, may produce more clinically severe disease.

Our pangenome analysis also shows gene duplication as a potential mechanism for drug resistance and tolerance. Our analyses revealed not only that an MmpL5-like protein is duplicated in several isolates across lineages but that this duplication was core in the lineage 1 pangenome. *mmpL5* has long been implicated in bedaquiline and clofazimine resistance through its ability to form a complex with membrane fusion protein mycobacterial membrane protein small 5 (MmpS5, encoded by *Rv0677c*) to transport drug compounds outside of the cell (139). Although overexpression of this operon through mutation in its repressor (*Rv0678*) has been shown to cause drug resistance, other mechanisms of overexpression such as duplication of genes and/or their paralogs have not been explored previously (140, 141). Importantly, given that the resistance gained by mutations in *Rv0678* is lost if there is a loss of function in *mmpL5* (142), it is also essential to assess whether the presence of an additional MmpL5-like protein may restore resistance to bedaquiline or cause drug tolerance. In addition, phenotypic consequences of changes in the expression of *mmpL1* or *mmpS1* (genes also important in transmembrane transportation) have not been investigated. Given that the MmpL gene family encodes for multidrug efflux pumps and undergoes partial if not whole gene duplication (143), it will be important to assess clinical and phenotypic relevance with increased expression of genes in the resistance-nodulation-cell-division superfamily (RND family) (144).

Similarly, analysis of gene duplications in one extensively drug-resistant isolate in our sample set revealed a duplicate copy of each gene in the *embC-A* operon which is implicated in ethambutol resistance. These genes encode for arabinosyl transferases which are important for arabinan biosynthesis needed to produce arabinogalactan in the cell wall (145). Although this was seen in only one isolate, 96 isolates had a duplication of an EmbR-like protein. The transcription factor *embR* is hypothesized to regulate the *embC-A* operon through its phosphorylation by PknH (146), as well as potentially regulate response to hypoxic stress (147). Duplications of these genes may provide a physical means of resistance or tolerance through increased cell well density, as noted elsewhere (148). Also, gene duplication may compensate for loss of function mutations and confound molecular diagnostic tests. Overall, gene duplications may provide another method to confer drug resistance or tolerance, as increased gene dosage may enhance the function of drug efflux pumps and cell wall synthesis. Duplications and duplications of their paralogs may compromise molecular diagnostic testing, which will need to adapt not just for single nucleotide polymorphisms but also gene copy numbers or compensatory proteins.

While our pangenome analysis and others assert that MTBC may have an open pangenome, some studies have concluded the opposite. There are several contending facts that complicate calling the pangenome open or closed. MTBC strains are clonal species with minimal diversity compared to other bacteria, making a closed pangenome plausible. MTBC strains have no horizontal gene transfer, which differs from the canonical open pangenome seen in bacteria with horizontal gene transfer, such as *E. coli* (114). However, our analyses show that MTBC exhibits diversity through gene duplication events and structural changes in gene order which likely aid survival and pathogenic success (68). Also, roughly 5% of the clinical isolates’ genomes were comprised of unnamed *ab initio* predicted genes (putative novel genes), giving more support to an open pangenome. Seeing that other pangenome studies with greater sample sizes (>1,000 isolates) reported slightly smaller core and larger accessory genomes than our study (54, 56)(Fig. S11), we conclude that the MTBC pangenome likely is open, despite having a slow rate of increase as indicated by our Heaps alpha metric.

Our analyses also highlighted important differences between our diverse set of clinical isolates and the H37Rv reference genome. While the proportion of putative novel genes remained consistent across clinical isolates, H37Rv contained a smaller number and proportion of these genes. These differences resulted in H37Rv clustering separate from all other isolates, including other lineage 4 isolates. Removing H37Rv from the L4 lineage-specific pangenome analysis increased the number of genes shared across all L4 isolates from 3,678 to 3,826 genes. This increase in L4 core genes suggests the genomic composition of H37Rv differed from other isolates within the L4 lineage but does not solely explain the difference in diversity seen in L4 compared to other lineages. The notable difference between H37Rv and the rest of L4 may be attributed to the fact that H37Rv has become a laboratory strain for the past several decades and has undergone a separate evolutionary path. This underscores the genomic differences in H37Rv from clinical isolates acknowledged by others (29, 40). One study of publicly available clinical isolates showed that H37Rv did not reflect the genomic structure observed in hypervariable regions, such as the *ppe38* region. A study of Beijing lineage isolates found 212 genes in their genomes that were not in H37Rv (149, 150). These studies and ours emphasize the caution of utilizing H37Rv for reference-based alignment of whole genomes, as a model strain for understanding TB infection in the host and spotlight the immense knowledge gap regarding the functional role of the putative novel genes in the MTBC genome.

A common theme among genes contributing to *Mtb* pangenomic diversity is frequent insertion by IS6110. The *ppe* genes with the highest information content (*ppe16* and *ppe34*), the genes identified by pangenome network analysis (the *Rv2816c-Rv2821c* and *plc* genes), and several resistance-associated structural variants all occur near, and suggest, IS6110 transposition. Importantly, not all genetic diversification that drives infection outcomes are captured by consensus sequence in cultured isolates; only those that are expelled and successfully transmitted to others will be captured. Isolates with a higher frequency of genomic compositional change will more frequently evolve subpopulations, some of which will contribute to worse infection outcomes. Our observations here are interesting in the context of Beijing isolates given their higher IS6110 copy number, their rapid global spread, and worse clinical outcomes for patients infected by them.

Our study contained a few limitations. Our study focused on *in silico* pangenome analyses and therefore requires further experimental confirmation of the effect of our findings on clinical outcomes and drug resistance. Because our data set is heavily representative of isolates from lineages 1, 2, and 4 and drug-resistant phenotypes, the pangenome does not reflect the entire *M. tuberculosis* complex species. The fixed effects model regressing the Panaroo structural variants on antibiotic resistance profiles did not reach statistical significance, likely due to sample size and an imbalance of resistant/susceptible isolates (Table S13). In addition, Panaroo’s gene reporting depends on the annotated inputs from Prokka to construct the pangenome. Using different annotation tools or parameters may produce different gene boundary calls, and thus may slightly vary in the number of genes identified, especially of the putative novel genes and gene fragments. Although Panaroo corrects for inaccurate gene labeling, it may struggle to identify genes with significant mutations and gene conversion events. Also, our estimation of an open pangenome does not consider the genes that are fragmented and thus potentially pseudogenized. While gene fragmentation adds to genetic diversity, not taking these into account could potentially overestimate pangenome growth. Lastly, given the high prevalence of frameshift mutations in the fragmented genes, we cannot rule out the possibility of sequencing error accounting for a small portion of our observed gene fragments. However, these genomes were sequenced with high depth (~100× on average) and used accurate P6C4 chemistry. PacBio continuous long-read sequencing is touted to maintain a low consensus error rate (108). Therefore, we can be confident that the observed gene fragmentation remains highly common.

### Conclusions

Our pangenome characterization of 109 *de novo* assembled genome of *Mtb* clinical isolates (and virulent type strain H37Rv) demonstrates conserved and variable regions among *M. tuberculosis* complex isolates. The variable regions can be attributed to manifold diversifying mechanisms, but IS6110 transposition was implicated especially frequently, particularly for diversifying genes with clear links to clinically pertinent phenotypic variation. This suggests that structural variation, and particularly IS6110 transposition, contributes to the high adaptability of this pathogen more so than currently appreciated. The pangenome is expanding, yet at a very slow rate, due to genetic duplications (including drug-resistant genes), fragmentations, and highly diverse structurally variable regions, which contribute to the balance of gene loss and gain. The slow rate of pangenome expansion can be explained by a genome that is mostly stable but highly dynamic and expanding in specific loci, such as *pe/ppe* genes. Clinically important areas of genetic variation included genes involved in host immune evasion (*Rv0071-73, Rv2817c, cas2*) and drug resistance (*mmpL5*, *embC-A*), which have significant implications for the development of vaccines and molecular diagnostics targeting these genes and their products.

## MATERIALS AND METHODS

### Pangenome analysis: preparation and global characterization

A pangenome was constructed using 109 phenotypically diverse (in terms of antibiotic resistance, Table S13, Set S7) clinical isolates of *M. tuberculosis* complex infection collected from the World Health Organization Supra-national References Laboratories in Stockholm, Sweden and Antwerp, Belgium, and the Global Consortium for Drug-resistant tuberculosis Diagnostics (GCDD) (PRJNA555636 and PRJEB8783), which have been previously described (81, 82, 88, 151). These sequencing data were collected between 2015 and 2019 on the Pacific Biosciences RSII instrument using P6-C4 chemistry and we previously assembled many of them in these aforementioned works. More details on genome assembly and quality control analyses are in the supplemental methods. Although four isolates have increased deletion rates compared to other isolates, these genomes were retained in the analysis since they met all other QC standards and did not differ greatly in our pangenome analysis (Fig. S12). Lineages were called using TBProfiler version 4.1.1 and database version 7fc199d (152). The GFF annotation files of these *de novo* assembled genomes were produced by Prokka version 1.14.6 using the following input parameters: --genus Mycobacterium --kingdom bacteria --rfam --rnammer --gram pos –usegenus –coverage 0.95 (153). Using the --proteins flag, we also provided Prokka with H37Rv protein sequences based on a recently curated annotation of this reference genome (154). With the addition of the H37Rv reference strain GFF annotation file from the National Center of Biotechnology Information (NCBI) (H37Rv-NC_000962.3), these files were input into Panaroo version 1.3.0, a pangenome analysis software that utilizes a graph-based algorithm to construct the pangenome based on gene adjacency (78). Panaroo was run with default parameters to produce an output file of gene presence and absence by isolating FASTA sequences of representative genes in the pangenome, a Panaroo-generated pangenome graph, and the sequence alignment of the core pangenome.

### Gene essentiality

To evaluate the distribution of genes experimentally identified as essential through *in vitro* transposon mutagenesis studies (TnSeq) and a CRISPRi study (87), supplemental data from DeJesus et al.(80), Zhang et al. (86), and Bosch et al. were merged with the Panaroo output. In accordance with DeJesus et al., essentiality was defined as a multilevel categorical variable (80). Similarly, essentiality according to Zhang et al. was a multi-level categorical variable (86); essentiality was also evaluated as a dichotomous variable according to the Bosch et al. definition. Essentiality was also evaluated as a binary covariate according to the DeJesus et al. definition, with “essential” being recoded to include “essential gene,” “essential domain,” and “growth advantage,” while “not essential” included “growth defect,” “not essential,” and “uncertain.” The proportion of binary essential genes in the accessory pangenome (i.e., comprised of genes that were in less than 99% of isolates) was also evaluated as a continuous variable.

Enrichment of genes implicated in the literature as functionally important or producing clinically relevant phenotypes from *in vivo* mouse models were also evaluated. The data sets used here include genes that are known transcription factors (155), produce toxins and antitoxins, are in *ppe*/*pe* gene families, have MamA, MamB, or MamC DNA methylation motif sites in their promoters (88), have effects on drug tolerance or resistance in mouse model (156), whose deletion mutants are underrepresented in TB mouse model (156), are in the enduring hypoxic response stimulon (89), are in the DosR regulon (89), or are conserved across other *Mycobacterial* species, specifically *M. leprae, M. smegmatis, M. avium,* and *M. abscessus* (157). Genes used in this enrichment analysis were not exclusively in either the core or accessory genomes. Instead, a core gene (i.e., in 99% of isolates) with an accessory duplicate could be represented both in the core and accessory genomes.

### Shannon’s entropy

We used Shannon’s entropy to identify which gene family’s diversity (as defined by gene presence/absence) carries the highest information content and therefore has the potential for the most impact on the strains’ phenotypic diversity. Shannon’s entropy is calculated as follows:


$$H=-\sum_{i=1}^{S}P_{i}ln\left(P_{i}\right)$$

where *S* is the total number of genes in a family, *i* is the gene index, and *P_i_* is the ratio of isolates whose genome harbors gene *i* over a total number of isolates (101, 102). Gene duplications and merged annotations were included in this analysis.

### Principal component analysis and cluster analysis

We conducted principal component analysis (PCA) and unsupervised hierarchical cluster analysis to compare genomic profiles constructed from gene presence/absence data between isolates. Binary presence and absence data of genes across the 110 queried genomes was input to construct both PCA biplots and heatmaps, demonstrating the patterns of gene presence in the core and accessory genomes across lineages. PCA loading scores for individual genes and their respective Pearson correlation *r*^2^ values were evaluated to assess which genes were most highly correlated to the principal components. All PCAs and figures were conducted using the PCAtools package in R (158).

### GO enrichment analysis

Functional gene enrichment of the gene clusters found in the core and accessory pangenomes was conducted using gene ontology (GO) analysis via PANTHER (159). Over-representation of biological process, molecular function, and cellular component GO terms present in the pangenome core and accessory genomes was assessed for significance using a Fisher’s exact test at *P* < 0.01 and corrected for multiple hypothesis testing using a false discovery rate against the background of the *M. tuberculosis* curated gene set collection hosted on the PANTHER database. These enrichments were also assessed by lineage to find potential functional distinctions of conserved function among isolates according to this factor.

### Putative novel genes

Prokka v. 1.14.6 was run on the H37Rv FASTA file from NCBI (H37Rv-NC_000962.3) with the same input parameters as the clinical isolates to identify which Panaroo-identified unique genes were, in fact, present in H37Rv as unnamed open reading frames. The open reading frames that were not assigned a gene name in the Prokka-produced GFF of H37Rv were extracted. The start and stop locations of these open reading frames were compared with the H37Rv-NCBI curated GFF file to filter out open reading frames that overlapped in nucleotide position with other annotated genes. The nucleotide sequences from the remaining unnamed open reading frames were aligned to the Panaroo-derived reference multi-FASTA file using BLASTN (160) to identify putative novel genes in our pangenome that are missed from the NCBI version of the H37Rv annotation.

We then sought to roughly characterize from which genes the putative novel genes may be derivatives as gene duplicates or gene fragments. Thus, Panaroo-derived sequences of these genes were all aligned to the NCBI H37Rv genome using BLASTN with default parameters. The best hits according to percent identity and query coverage were reported.

### Gene fragmentations and power law curve

Potential fragments were identified by Panaroo as having multiple Prokka-generated IDs in a single locus in the binary presence/absence output. Panaroo routinely combines fragmented genes as a result of a fragmented assembly (78). However, since our genomes were constructed with long read sequencing and a robust assembly pipeline, we conclude that these Prokka-identified open reading frames disrupting normal gene coordinates are likely to be genes fragmented by an early stop codon. The isolates and their genes with potential fragments were indexed, and their gene positions and lengths were compared to the corresponding gene length in H37Rv to identify which were tandem duplications versus fragments whose summed coverage equaled that of the copy in H37Rv. Since most putative novel genes were not in H37Rv, the lengths of these putative novel genes with putative fragments were compared to their average sequence length in the pangenome. Genes with multiple different genes annotated at a single locus (i.e., merged annotations, Fig. S8) were also excluded from this analysis.

A pangenome and core genome rarefaction simulation was conducted to estimate the number of genes in the core or pangenome given 100 random permutations of increasingly larger sample sizes of genomes (113). The pangenome rarefaction simulation was then used to construct the power law curve, while the core genome rarefaction simulation was used to fit an exponential decay curve. Collectively, these two curves are graphed to create the pangenome curves, which reflect the tradeoff between core and accessory gene cluster gain as the pangenome size expands with the stepwise addition of more isolates (113). The Heaps alpha value was calculated according to the Heaps law model described elsewhere (114) to inform if the pangenome is open (i.e., increases in genomic content with the addition of isolates) or closed (i.e., the number of genes do not continue to increase with more isolates, suggesting a limit to the bacterial genomic content). These analyses and graphs were constructed in R with the Pagoo package using default parameters (113).

### Network analysis

A fixed-effects linear model with a binomial family and a population structure correction using mash-calculated distances was conducted for structural variants output by Panaroo. The models were executed against binary drug resistance profiles (Resistant/Susceptible/Not Determined) for the 110 genomes in this data set using Pyseer v.1.3.10 (161). The phenotypes tested included the following: pyrazinamide, fluoroquinolone, ofloxacin, moxifloxacin, kanamycin, capreomycin, amikacin, isoniazid, and rifampicin resistance. Beta coefficients of all models were exponentiated to calculate the odds ratios, and confidence intervals based on beta coefficient standard errors were also calculated. A correction for multiple hypothesis testing was determined by assessing the FDR using p.adjust() in base R v.4.2.2 (162).

The adjacency-derived network graph’s layout was simplified with the gene-adjacency information for isolate SEA11278, a monoresistant lineage 7 isolate (biosample SAMN12325265 bioproject PRJNA555636) that is most dissimilar (demonstrated through hierarchical clustering analysis; Fig. 1) to the other isolates present in this pangenome, then circularized for downstream network analysis. Using Cytoscape 3.9.1 (163), a standard network analysis approach was used to query the network for potential drivers of SVs by extracting areas of the genome where the gene neighborhood contained more than two nodes with more than two degrees. This threshold served as the distinguishing factor for local gene neighborhoods of high adjacency variation. Centrality measures for all nodes in the pangenome graph including global network measures were calculated using Cytoscape NetworkAnalyzer (164), which reported on network size (defined as the presence of nodes across all genomes included in this networks analysis), degree, average shortest path length, clustering coefficient, betweenness centrality, neighborhood connectivity, radiality, closeness centrality, eccentricity, and topological coefficient. Long-range connections (i.e., edges between the main pangenome network graph and unsupported nodes removed due to their trans-network connections) were characterized by size and functional enrichment using GO analysis through PANTHER. Networks were visualized and analyzed in Cytoscape, with all other data cleaning and analyses done in R4.1.0 (162), Rstudio 1.4.2.1, and Python 3.9.7 (165).

### Statistical analyses

Comparisons of genome size, lineage-specific pangenomes, and percent of essential genes across lineages were evaluated using one-way Welch’s ANOVA and Games-Howell test for multiple pairwise comparisons due to unequal variances across the lineages. Kruskal-Wallis and Dunn’s tests were used to evaluate statistical differences in the number of fragmented and duplicated genes across lineages, as data for these variables necessitated non-parametric tests. Since the H37Rv-NCBI GFF lacked the annotation of any putative novel genes and fragmented genes, it was identified as an outlier and removed from these bivariate statistical analyses. Lineages 6 and 7 were also removed from these statistical tests due to low sample size. Lineage 5 isolates were also removed from Kruskal-Wallis tests due to low sample size.

As essentiality studies were defined in the context of genes in H37Rv, bivariate analyses were restricted to named genes within H37Rv, and thus we removed 488 putative novel genes and merged annotations (i.e., loci in the pangenome where different isolates had different and at times several genes called, Fig. S8). Also, we restricted the analysis to genes with no missing essentiality data (DeJesus et al.: 3,766/3,837 [98%], Zhang et al.: 3,739/3,837 [97%], Bosch et al.: 3,837/3,935 [98%]) or functional enrichment data (Table 2). Bivariate relationships for essentiality were evaluated using Fisher’s exact test to assess enrichment in the core versus accessory genomes. *P*-values were adjusted for FDR due to the large number of multiple comparison tests. Simple logistic regression models provided quantified estimates of the size of enrichment, with model fit estimates used to determine the appropriateness of the models.

## Supplementary Material

Reviewer comments

## Data Availability

Complete genomes for all 109 *M*. *tuberculosis* complex clinical strains are deposited under BioProject accessions PRJNA555636 and PRJEB8783. Custom codes for pangenome analyses are located at https://gitlab.com/LPCDRP/pangenome.

## SUPPLEMENTAL MATERIAL

Data Set S1 msystems.00499-24-s0001.xls Published Mtb pangenomes.

Data Set S2 msystems.00499-24-s0002.xlsx List of all genes present in pangenomes.

Data Set S3 msystems.00499-24-s0003.csv List of all genes present in lineage 4.

Data Set S4 msystems.00499-24-s0004.xlsx Shannon's entropy scores of PPE, ESX, PGRS, and PE genes.

Data Set S5 msystems.00499-24-s0005.xlsx Unique, non-merged genes by gene family with their representative sequences.

Data Set S6 msystems.00499-24-s0006.xlsx Unnamed *ab initio* predicted genes identified by Panaroo.

Data Set S7 msystems.00499-24-s0007.xlsx Study isolate pDST, lineage, and NCBI Biosample accessions.

Supplemental material msystems.00499-24-s0008.docx Supplemental figures and tables.

OPEN PEER REVIEW reviewer-comments.pdf An accounting of the reviewer comments and feedback.

ASM does not own the copyrights to Supplemental Material that may be linked to, or accessed through, an article. The authors have granted ASM a non-exclusive, world-wide license to publish the Supplemental Material files. Please contact the corresponding author directly for reuse.

## References

[1] van Soolingen D, Qian L, de Haas PE, Douglas JT, Traore H, Portaels F, Qing HZ, Enkhsaikan D, Nymadawa P, van Embden JD. 1995. Predominance of a single genotype of Mycobacterium tuberculosis in countries of east Asia. J Clin Microbiol 33:3234–3238. doi:10.1128/jcm.33.12.3234-3238.1995 8586708 PMC228679

[2] Gagneux S. 2018. Ecology and evolution of Mycobacterium tuberculosis. Nat Rev Microbiol 16:202–213. doi:10.1038/nrmicro.2018.8 29456241

[3] Organization WH. 2021. Global tuberculosis report 2021.

[4] Larson N. 2022. The world’s biggest infectious killer regains its deadly lead. Available from: https://www.sciencealert.com/the-worlds-biggest-infectious-killer-regains-its-deadly-lead. Retrieved 30 Nov 2022.

[5] Paulson T. 2013. Epidemiology: a mortal foe. Nature 502:S2–3. doi:10.1038/502S2a 24108078

[6] Werngren J, Alm E, Mansjö M. 2017. Non-pncA gene-mutated but pyrazinamide-resistant mycobacterium tuberculosis: why is that? J Clin Microbiol 55:1920–1927. doi:10.1128/JCM.02532-16 28404681 PMC5442549

[7] Domenech P, Zou J, Averback A, Syed N, Curtis D, Donato S, Reed MB. 2017. Unique regulation of the DosR regulon in the beijing lineage of Mycobacterium tuberculosis. J Bacteriol 199:e00696-16. doi:10.1128/JB.00696-16 27799329 PMC5198487

[8] de Keijzer J, de Haas PE, de Ru AH, van Veelen PA, van Soolingen D. 2014. Disclosure of selective advantages in the “modern” sublineage of the Mycobacterium tuberculosis Beijing genotype family by quantitative proteomics. Mol Cell Proteomics 13:2632–2645. doi:10.1074/mcp.M114.038380 25022876 PMC4188992

[9] Galagan JE. 2014. Genomic insights into tuberculosis. Nat Rev Genet 15:307–320. doi:10.1038/nrg3664 24662221

[10] Peters JS, Ismail N, Dippenaar A, Ma S, Sherman DR, Warren RM, Kana BD. 2020. Genetic diversity in Mycobacterium tuberculosis clinical isolates and resulting outcomes of tuberculosis infection and disease. Annu Rev Genet 54:511–537. doi:10.1146/annurev-genet-022820-085940 32926793 PMC12646202

[11] Gevers D, Vandepoele K, Simillon C, Van de Peer Y. 2004. Gene duplication and biased functional retention of paralogs in bacterial genomes. Trends Microbiol 12:148–154. doi:10.1016/j.tim.2004.02.007 15116722

[12] Liu X, Gutacker MM, Musser JM, Fu YX. 2006. Evidence for recombination in Mycobacterium tuberculosis. J Bacteriol 188:8169–8177. doi:10.1128/JB.01062-06 16997954 PMC1698211

[13] Boritsch EC, Khanna V, Pawlik A, Honoré N, Navas VH, Ma L, Bouchier C, Seemann T, Supply P, Stinear TP, Brosch R. 2016. Key experimental evidence of chromosomal DNA transfer among selected tuberculosis-causing mycobacteria. Proc Natl Acad Sci U S A 113:9876–9881. doi:10.1073/pnas.1604921113 27528665 PMC5024641

[14] Chiner-Oms Á, Sánchez-Busó L, Corander J, Gagneux S, Harris SR, Young D, González-Candelas F, Comas I. 2019. Genomic determinants of speciation and spread of the Mycobacterium tuberculosis complex. Sci Adv 5:eaaw3307. doi:10.1126/sciadv.aaw3307 31448322 PMC6691555

[15] Hershberg R, Lipatov M, Small PM, Sheffer H, Niemann S, Homolka S, Roach JC, Kremer K, Petrov DA, Feldman MW, Gagneux S. 2008. High functional diversity in Mycobacterium tuberculosis driven by genetic drift and human demography. PLoS Biol 6:e311. doi:10.1371/journal.pbio.0060311 19090620 PMC2602723

[16] Domenech P, Kolly GS, Leon-Solis L, Fallow A, Reed MB. 2010. Massive gene duplication event among clinical isolates of the Mycobacterium tuberculosis W/Beijing family. J Bacteriol 192:4562–4570. doi:10.1128/JB.00536-10 20639330 PMC2937425

[17] Shitikov EA, Bespyatykh JA, Ischenko DS, Alexeev DG, Karpova IY, Kostryukova ES, Isaeva YD, Nosova EY, Mokrousov IV, Vyazovaya AA, Narvskaya OV, Vishnevsky BI, Otten TF, Zhuravlev VI, Yablonsky PK, Ilina EN, Govorun VM. 2014. Unusual large-scale chromosomal rearrangements in Mycobacterium tuberculosis Beijing B0/W148 cluster isolates. PLoS One 9:e84971. doi:10.1371/journal.pone.0084971 24416324 PMC3885621

[18] Zhang J. 2003. Evolution by gene duplication: an update. Trends Ecol Evol (Amst) 18:292–298. doi:10.1016/S0169-5347(03)00033-8

[19] Ohno S. 1970. Evolution by gene duplication. Springer, Berlin, Germany.

[20] Bolotin E, Hershberg R. 2015. Gene loss dominates as a source of genetic variation within clonal pathogenic bacterial species. Genome Biol Evol 7:2173–2187. doi:10.1093/gbe/evv135 26163675 PMC4558853

[21] Gordon SV, Brosch R, Billault A, Garnier T, Eiglmeier K, Cole ST. 1999. Identification of variable regions in the genomes of tubercle bacilli using bacterial artificial chromosome arrays. Mol Microbiol 32:643–655. doi:10.1046/j.1365-2958.1999.01383.x 10320585

[22] Brosch R, Philipp WJ, Stavropoulos E, Colston MJ, Cole ST, Gordon SV. 1999. Genomic analysis reveals variation between Mycobacterium tuberculosis H37Rv and the attenuated M. tuberculosis H37Ra strain. Infect Immun 67:5768–5774. doi:10.1128/IAI.67.11.5768-5774.1999 10531227 PMC96953

[23] Bespiatykh D, Bespyatykh J, Mokrousov I, Shitikov E. 2021. A comprehensive map of Mycobacterium tuberculosis complex regions of difference. mSphere 6:e00535-21. doi:10.1128/mSphere.00535-21 34287002 PMC8386458

[24] Levillain F, Poquet Y, Mallet L, Mazères S, Marceau M, Brosch R, Bange F-C, Supply P, Magalon A, Neyrolles O. 2017. Horizontal acquisition of a hypoxia-responsive molybdenum cofactor biosynthesis pathway contributed to Mycobacterium tuberculosis pathoadaptation. PLOS Pathog 13:e1006752. doi:10.1371/journal.ppat.1006752 29176894 PMC5720804

[25] Brosch R, Gordon SV, Marmiesse M, Brodin P, Buchrieser C, Eiglmeier K, Garnier T, Gutierrez C, Hewinson G, Kremer K, Parsons LM, Pym AS, Samper S, van Soolingen D, Cole ST. 2002. A new evolutionary scenario for the Mycobacterium tuberculosis complex. Proc Natl Acad Sci U S A 99:3684–3689. doi:10.1073/pnas.052548299 11891304 PMC122584

[26] Yang T, Zhong J, Zhang J, Li C, Yu X, Xiao J, Jia X, Ding N, Ma G, Wang G, Yue L, Liang Q, Sheng Y, Sun Y, Huang H, Chen F. 2018. Pan-genomic study of Mycobacterium tuberculosis reflecting the primary/secondary genes, generality/individuality, and the interconversion through copy number variations. Front Microbiol 9:1886. doi:10.3389/fmicb.2018.01886 30177918 PMC6109687

[27] Charles C, Conde C, Vorimore F, Cochard T, Michelet L, Boschiroli ML, Biet F. 2023. Features of Mycobacterium bovis complete genomes belonging to 5 different lineages. Microorganisms 11:177. doi:10.3390/microorganisms11010177 36677470 PMC9865570

[28] Mei Y-M, Zhang W-Y, Sun J-Y, Jiang H-Q, Shi Y, Xiong J-S, Wang L, Chen Y-Q, Long S-Y, Pan C, Luo T, Wang H-S. 2023. Genomic characteristics of Mycobacterium tuberculosis isolates of cutaneous tuberculosis. Front Microbiol 14:1165916. doi:10.3389/fmicb.2023.1165916 37266022 PMC10230547

[29] Sanoussi CN, Coscolla M, Ofori-Anyinam B, Otchere ID, Antonio M, Niemann S, Parkhill J, Harris S, Yeboah-Manu D, Gagneux S, Rigouts L, Affolabi D, de Jong BC, Meehan CJ. 2021. Mycobacterium tuberculosis complex lineage 5 exhibits high levels of within-lineage genomic diversity and differing gene content compared to the type strain H37Rv. Microb Genom 7:000437. doi:10.1099/mgen.0.000437 34241588 PMC8477398

[30] Silva-Pereira TT, Soler-Camargo NC, Guimarães AMS. 2024. Diversification of gene content in the Mycobacterium tuberculosis complex is determined by phylogenetic and ecological signatures. Microbiol Spectr 12:e02289-23. doi:10.1128/spectrum.02289-23 38230932 PMC10871547

[31] Dumas E, Christina Boritsch E, Vandenbogaert M, Rodríguez de la Vega RC, Thiberge J-M, Caro V, Gaillard J-L, Heym B, Girard-Misguich F, Brosch R, Sapriel G. 2016. Mycobacterial pan-genome analysis suggests important role of plasmids in the radiation of type VII secretion systems. Genome Biol Evol 8:387–402. doi:10.1093/gbe/evw001 26748339 PMC4779608

[32] Tariq D-E-S. 2024. Pangenomic analyses of tuberculosis strains to identify resistomes using computational approaches. J Pak Med Assoc 74:S74–S78. doi:10.47391/JPMA-DUHS-S15 38385476

[33] Marin MG, Wippel C, Quinones-Olvera N, Behruznia M, Jeffrey BM, Harris M, Mann BC, Rosenthal A, Jacobson KR, Warren RM, Li H, Meehan CJ, Farhat MR. 2024. Analysis of the limited M. tuberculosis accessory genome reveals potential pitfalls of pan-genome analysis approaches. bioRxiv:2024.03.21.586149. doi:10.1101/2024.03.21.586149

[34] Stoler N, Nekrutenko A. 2021. Sequencing error profiles of Illumina sequencing instruments. NAR Genomics Bioinform 3:lqab019. doi:10.1093/nargab/lqab019 PMC8002175 33817639

[35] Modlin SJ, Robinhold C, Morrissey C, Mitchell SN, Ramirez-Busby SM, Shmaya T, Valafar F. 2021. Exact mapping of Illumina blind spots in the Mycobacterium tuberculosis genome reveals platform-wide and workflow-specific biases. Microb Genom 7:000465. doi:10.1099/mgen.0.000465 PMC8190613 33502304

[36] Salzberg SL, Phillippy AM, Zimin A, Puiu D, Magoc T, Koren S, Treangen TJ, Schatz MC, Delcher AL, Roberts M, Marçais G, Pop M, Yorke JA. 2012. GAGE: a critical evaluation of genome assemblies and assembly algorithms. Genome Res 22:557–567. doi:10.1101/gr.131383.111 22147368 PMC3290791

[37] Marin M, Vargas R, Harris M, Jeffrey B, Epperson LE, Durbin D, Strong M, Salfinger M, Iqbal Z, Akhundova I, Vashakidze S, Crudu V, Rosenthal A, Farhat MR. 2022. Benchmarking the empirical accuracy of short-read sequencing across the M. tuberculosis genome. Bioinformatics 38:1781–1787. doi:10.1093/bioinformatics/btac023 35020793 PMC8963317

[38] Ross MG, Russ C, Costello M, Hollinger A, Lennon NJ, Hegarty R, Nusbaum C, Jaffe DB. 2013. Characterizing and measuring bias in sequence data. Genome Biol 14:R51. doi:10.1186/gb-2013-14-5-r51 23718773 PMC4053816

[39] Cole ST, Brosch R, Parkhill J, Garnier T, Churcher C, Harris D, Gordon SV, Eiglmeier K, Gas S, Barry CE 3rd, et al.. 1998. Deciphering the biology of Mycobacterium tuberculosis from the complete genome sequence. Nature 393:537–544. doi:10.1038/31159 9634230

[40] O’Toole RF, Gautam SS. 2017. Limitations of the Mycobacterium tuberculosis reference genome H37Rv in the detection of virulence-related loci. Genomics 109:471–474. doi:10.1016/j.ygeno.2017.07.004 28743540

[41] Zhao X, Collins RL, Lee W-P, Weber AM, Jun Y, Zhu Q, Weisburd B, Huang Y, Audano PA, Wang H, Walker M, Lowther C, Fu J, Human Genome Structural Variation Consortium, Gerstein MB, Devine SE, Marschall T, Korbel JO, Eichler EE, Chaisson MJP, Lee C, Mills RE, Brand H, Talkowski ME. 2021. Expectations and blind spots for structural variation detection from long-read assemblies and short-read genome sequencing technologies. Am J Hum Genet 108:919–928. doi:10.1016/j.ajhg.2021.03.014 33789087 PMC8206509

[42] Mahmoud M, Gobet N, Cruz-Dávalos DI, Mounier N, Dessimoz C, Sedlazeck FJ. 2019. Structural variant calling: the long and the short of it. Genome Biol 20:246. doi:10.1186/s13059-019-1828-7 31747936 PMC6868818

[43] Koren S, Phillippy AM, Simpson JT, Loman NJ, Loose M. 2019. Reply to “Errors in long-read assemblies can critically affect protein prediction”. Nat Biotechnol 37:127–128. doi:10.1038/s41587-018-0005-y 30670797

[44] Zhu L, Zhong J, Jia X, Liu G, Kang Y, Dong M, Zhang X, Li Q, Yue L, Li C, Fu J, Xiao J, Yan J, Zhang B, Lei M, Chen S, Lv L, Zhu B, Huang H, Chen F. 2016. Precision methylome characterization of Mycobacterium tuberculosis complex (MTBC) using PacBio single-molecule real-time (SMRT) technology. Nucleic Acids Res 44:730–743. doi:10.1093/nar/gkv1498 26704977 PMC4737169

[45] Elghraoui A, Modlin SJ, Valafar F. 2017. SMRT genome assembly corrects reference errors, resolving the genetic basis of virulence in Mycobacterium tuberculosis. BMC Genomics 18:302. doi:10.1186/s12864-017-3687-5 28415976 PMC5393005

[46] Berney M, Berney-Meyer L, Wong K-W, Chen B, Chen M, Kim J, Wang J, Harris D, Parkhill J, Chan J, Wang F, Jacobs WR Jr. 2015. Essential roles of methionine and S-adenosylmethionine in the autarkic lifestyle of Mycobacterium tuberculosis. Proc Natl Acad Sci U S A 112:10008–10013. doi:10.1073/pnas.1513033112 26221021 PMC4538671

[47] Phelan JE, Coll F, Bergval I, Anthony RM, Warren R, Sampson SL, Gey van Pittius NC, Glynn JR, Crampin AC, Alves A, et al.. 2016. Recombination in pe/ppe genes contributes to genetic variation in Mycobacterium tuberculosis lineages. BMC Genomics 17:151. doi:10.1186/s12864-016-2467-y 26923687 PMC4770551

[48] Bradley P, Gordon NC, Walker TM, Dunn L, Heys S, Huang B, Earle S, Pankhurst LJ, Anson L, de Cesare M, et al.. 2015. Rapid antibiotic-resistance predictions from genome sequence data for Staphylococcus aureus and Mycobacterium tuberculosis. Nat Commun 6:10063. doi:10.1038/ncomms10063 26686880 PMC4703848

[49] Votintseva AA, Bradley P, Pankhurst L, Del Ojo Elias C, Loose M, Nilgiriwala K, Chatterjee A, Smith EG, Sanderson N, Walker TM, Morgan MR, Wyllie DH, Walker AS, Peto TEA, Crook DW, Iqbal Z. 2017. Same-day diagnostic and surveillance data for tuberculosis via whole-genome sequencing of direct respiratory samples. J Clin Microbiol 55:1285–1298. doi:10.1128/JCM.02483-16 28275074 PMC5405248

[50] Bainomugisa A, Duarte T, Lavu E, Pandey S, Coulter C, Marais BJ, Coin LM. 2018. A complete high-quality MinION nanopore assembly of an extensively drug-resistant Mycobacterium tuberculosis Beijing lineage strain identifies novel variation in repetitive PE/PPE gene regions. Microb Genom 4:000188. doi:10.1099/mgen.0.000188 PMC6113869 29906261

[51] Murphy SG, Smith C, Lapierre P, Shea J, Patel K, Halse TA, Dickinson M, Escuyer V, Rowlinson MC, Musser KA. 2023. Direct detection of drug-resistant Mycobacterium tuberculosis using targeted next generation sequencing. Front Public Health 11. doi:10.3389/fpubh.2023.1206056 PMC10340549 37457262

[52] Fleischmann RD, Alland D, Eisen JA, Carpenter L, White O, Peterson J, DeBoy R, Dodson R, Gwinn M, Haft D, et al.. 2002. Whole-genome comparison of Mycobacterium tuberculosis clinical and laboratory strains. J Bacteriol 184:5479–5490. doi:10.1128/JB.184.19.5479-5490.2002 12218036 PMC135346

[53] Roetzer A, Diel R, Kohl TA, Rückert C, Nübel U, Blom J, Wirth T, Jaenicke S, Schuback S, Rüsch-Gerdes S, Supply P, Kalinowski J, Niemann S. 2013. Whole genome sequencing versus traditional genotyping for investigation of A Mycobacterium tuberculosis outbreak: a longitudinal molecular epidemiological study. PLoS Med 10:e1001387. doi:10.1371/journal.pmed.1001387 23424287 PMC3570532

[54] Park S-C, Lee K, Kim YO, Won S, Chun J. 2019 Large-scale genomics reveals the genetic characteristics of seven species and importance of phylogenetic distance for estimating pan-genome size. Front Microbiol 10. doi:10.3389/fmicb.2019.00834 PMC6491781 31068915

[55] Rufai SB, Ozer EA, Singh S. 2020. Pan-genome analysis of Mycobacterium tuberculosis identifies accessory genome sequences deleted in modern Beijing lineage. bioRxiv. doi:10.1101/2020.12.01.407569

[56] Kavvas ES, Catoiu E, Mih N, Yurkovich JT, Seif Y, Dillon N, Heckmann D, Anand A, Yang L, Nizet V, Monk JM, Palsson BO. 2018. Machine learning and structural analysis of Mycobacterium tuberculosis pan-genome identifies genetic signatures of antibiotic resistance. Nat Commun 9:4306. doi:10.1038/s41467-018-06634-y 30333483 PMC6193043

[57] Olawoye IB, Frost SDW, Happi CT. 2020. Pan-genome analysis of Mycobacterium africanum insights to dynamics and evolution. bioRxiv. doi:10.1101/2020.03.17.995167.

[58] Periwal V, Patowary A, Vellarikkal SK, Gupta A, Singh M, Mittal A, Jeyapaul S, Chauhan RK, Singh AV, Singh PK, Garg P, Katoch VM, Katoch K, Chauhan DS, Sivasubbu S, Scaria V. 2015. Comparative whole-genome analysis of clinical isolates reveals characteristic architecture of Mycobacterium tuberculosis pangenome. PLoS One 10:e0122979. doi:10.1371/journal.pone.0122979 25853708 PMC4390332

[59] Li X, Lin J, Hu Y, Zhou J. 2020. PARMAP: a pan-genome-based computational framework for predicting antimicrobial resistance. Front Microbiol 11:578795. doi:10.3389/fmicb.2020.578795 33193203 PMC7642336

[60] Khan MF, Ali A, Rehman HM, Noor Khan S, Hammad HM, Waseem M, Wu Y, Clark TG, Jabbar A. 2024. Exploring optimal drug targets through subtractive proteomics analysis and pangenomic insights for tailored drug design in tuberculosis. Sci Rep 14:10904. doi:10.1038/s41598-024-61752-6 38740859 PMC11091173

[61] Kayani MR, Zheng Y-C, Xie F-C, Kang K, Li H-Y, Zhao H-T. 2018. Genome sequences and comparative analysis of two extended-spectrum extensively-drug resistant Mycobacterium tuberculosis strains. Front Pharmacol 9:1492. doi:10.3389/fphar.2018.01492 30618776 PMC6305476

[62] Dar HA, Zaheer T, Ullah N, Bakhtiar SM, Zhang T, Yasir M, Azhar EI, Ali A. 2020. Pangenome analysis of Mycobacterium tuberculosis reveals core-drug targets and screening of promising lead compounds for drug discovery. Antibiotics (Basel) 9:819. doi:10.3390/antibiotics9110819 33213029 PMC7698547

[63] Soler-Camargo NC, Silva-Pereira TT, Zimpel CK, Camacho MF, Zelanis A, Aono AH, Patané JS, dos Santos AP, Guimarães AMS. 2022. The rate and role of pseudogenes of the Mycobacterium tuberculosis complex. Microb Genom 8:000876. doi:10.1099/mgen.0.000876 PMC9676053 36250787

[64] Negrete-Paz AM, Vázquez-Marrufo G, Gutiérrez-Moraga A, Vázquez-Garcidueñas MS. 2023. Pangenome reconstruction of Mycobacterium tuberculosis as a guide to reveal genomic features associated with strain clinical phenotype. Microorganisms 11:1495. doi:10.3390/microorganisms11061495 37374997 PMC10300688

[65] Wan X, Koster K, Qian L, Desmond E, Brostrom R, Hou S, Douglas JT. 2017. Genomic analyses of the ancestral Manila family of Mycobacterium tuberculosis. PLoS One 12:e0175330. doi:10.1371/journal.pone.0175330 28394899 PMC5386241

[66] Zakham F, Sironen T, Vapalahti O, Kant R. 2021. Pan and core genome analysis of 183 Mycobacterium tuberculosis strains revealed a high inter-species diversity among the human adapted strains. Antibiotics (Basel) 10:500. doi:10.3390/antibiotics10050500 33924811 PMC8145561

[67] Zimpel CK, Brandão PE, de Souza Filho AF, de Souza RF, Ikuta CY, Ferreira Neto JS, Camargo NCS, Heinemann MB, Guimarães AMS. 2017. Complete genome sequencing of Mycobacterium bovis SP38 and comparative genomics of Mycobacterium bovis and M. tuberculosis strains. Front Microbiol 8:2389. doi:10.3389/fmicb.2017.02389 29259589 PMC5723337

[68] Periwal V, Scaria V. 2015. Insights into structural variations and genome rearrangements in prokaryotic genomes. Bioinformatics 31:1–9. doi:10.1093/bioinformatics/btu600 25189783

[69] Ceres KM, Stanhope MJ, Gröhn YT. 2022. A critical evaluation of Mycobacterium bovis pangenomics, with reference to its utility in outbreak investigation. Microb Genom 8:000839. doi:10.1099/mgen.0.000839 PMC9455707 35763423

[70] Hurtado-Páez U, Álvarez Zuluaga N, Arango Isaza RE, Contreras-Moreira B, Rouzaud F, Robledo J. 2022. Pan-genome association study of Mycobacterium tuberculosis lineage-4 revealed specific genes related to the high and low prevalence of the disease in patients from the North-Eastern area of Medellín, Colombia. Front Microbiol 13:1076797. doi:10.3389/fmicb.2022.1076797 36687645 PMC9846648

[71] Reis AC, Cunha MV. 2021. The open pan-genome architecture and virulence landscape of Mycobacterium bovis. Microb Genom 7. doi:10.1099/mgen.0.000664 PMC8627212 34714230

[72] Das S, Pettersson BMF, Behra PRK, Mallick A, Cheramie M, Ramesh M, Shirreff L, DuCote T, Dasgupta S, Ennis DG, Kirsebom LeifA. 2018. Extensive genomic diversity among Mycobacterium marinum strains revealed by whole genome sequencing. Sci Rep 8:12040. doi:10.1038/s41598-018-30152-y 30104693 PMC6089878

[73] Tateishi Y, Ozeki Y, Nishiyama A, Miki M, Maekura R, Fukushima Y, Nakajima C, Suzuki Y, Matsumoto S. 2021. Comparative genomic analysis of Mycobacterium intracellulare: implications for clinical taxonomic classification in pulmonary Mycobacterium avium-intracellulare complex disease. BMC Microbiol 21:103. doi:10.1186/s12866-021-02163-9 33823816 PMC8025370

[74] Richards VP, Nigsch A, Pavinski Bitar P, Sun Q, Stuber T, Ceres K, Smith RL, Robbe Austerman S, Schukken Y, Grohn YT, Stanhope MJ. 2021. Evolutionary genomic and bacteria GWAS analysis of Mycobacterium avium subsp. paratuberculosis and dairy cattle Johne’s disease phenotypes. Appl Environ Microbiol 87:e02570-20. doi:10.1128/AEM.02570-20 33547057 PMC8091108

[75] Uchiya K-I, Tomida S, Nakagawa T, Asahi S, Nikai T, Ogawa K. 2017. Comparative genome analyses of Mycobacterium avium reveal genomic features of its subspecies and strains that cause progression of pulmonary disease. Sci Rep 7:39750. doi:10.1038/srep39750 28045086 PMC5206733

[76] Choo SW, Wee WY, Ngeow YF, Mitchell W, Tan JL, Wong GJ, Zhao Y, Xiao J. 2014. Genomic reconnaissance of clinical isolates of emerging human pathogen Mycobacterium abscessus reveals high evolutionary potential. Sci Rep 4:4061. doi:10.1038/srep04061 24515248 PMC3920477

[77] Karthik K, Subramanian S, Vinoli Priyadharshini M, Jawahar A, Anbazhagan S, Kathiravan RS, Thomas P, Babu RPA, Gopalan Tirumurugaan K, Raj GD. 2023. Whole genome sequencing and comparative genomics of Mycobacterium orygis isolated from different animal hosts to identify specific diagnostic markers. Front Cell Infect Microbiol 13:1302393. doi:10.3389/fcimb.2023.1302393 38188626 PMC10770871

[78] Tonkin-Hill G, MacAlasdair N, Ruis C, Weimann A, Horesh G, Lees JA, Gladstone RA, Lo S, Beaudoin C, Floto RA, Frost SDW, Corander J, Bentley SD, Parkhill J. 2020. Producing polished prokaryotic pangenomes with the Panaroo pipeline. Genome Biol 21:180. doi:10.1186/s13059-020-02090-4 32698896 PMC7376924

[79] Jiang Q, Qiu Y, Kurland IJ, Drlica K, Subbian S, Tyagi S, Shi L. 2022. Glutamine is required for M1-like polarization of macrophages in response to Mycobacterium tuberculosis infection. mBio 13:e01274-22. doi:10.1128/mbio.01274-22 35762591 PMC9426538

[80] DeJesus MA, Gerrick ER, Xu W, Park SW, Long JE, Boutte CC, Rubin EJ, Schnappinger D, Ehrt S, Fortune SM, Sassetti CM, Ioerger TR. 2017. Comprehensive essentiality analysis of the Mycobacterium tuberculosis genome via saturating transposon mutagenesis. MBio 8:e02133-16. doi:10.1128/mBio.02133-16 28096490 PMC5241402

[81] Rodwell TC, Valafar F, Douglas J, Qian L, Garfein RS, Chawla A, Torres J, Zadorozhny V, Kim MS, Hoshide M, Catanzaro D, Jackson L, Lin G, Desmond E, Rodrigues C, Eisenach K, Victor TC, Ismail N, Crudu V, Gler MT, Catanzaro A. 2014. Predicting extensively drug-resistant Mycobacterium tuberculosis phenotypes with genetic mutations. J Clin Microbiol 52:781–789. doi:10.1128/JCM.02701-13 24353002 PMC3957771

[82] Garfein RS, Catanzaro DG, Rodwell TC, Avalos E, Jackson RL, Kaping J, Evasco H, Rodrigues C, Crudu V, Lin S-YG, Groessl E, Hillery N, Trollip A, Ganiats T, Victor TC, Eisenach K, Valafar F, Channick J, Qian L, Catanzaro A. 2015. Phenotypic and genotypic diversity in a multinational sample of drug-resistant Mycobacterium tuberculosis isolates. Int J Tuberc Lung Dis 19:420–427. doi:10.5588/ijtld.14.0488 25859997 PMC4494864

[83] Stucki D, Brites D, Jeljeli L, Coscolla M, Liu Q, Trauner A, Fenner L, Rutaihwa L, Borrell S, Luo T, et al.. 2016. Mycobacterium tuberculosis lineage 4 comprises globally distributed and geographically restricted sublineages. Nat Genet 48:1535–1543. doi:10.1038/ng.3704 27798628 PMC5238942

[84] Micheni LN, Kassaza K, Kinyi H, Ntulume I, Bazira J. 2021. Diversity of Mycobacterium tuberculosis complex lineages associated with pulmonary tuberculosis in Southwestern, Uganda. Tuberc Res Treat 2021:5588339. doi:10.1155/2021/5588339 34306752 PMC8264515

[85] Qin L, Wang J, Lu J, Yang H, Zheng R, Liu Z, Huang X, Feng Y, Hu Z, Ge B. 2019. A deletion in the RD105 region confers resistance to multiple drugs in Mycobacterium tuberculosis. BMC Biol 17:7. doi:10.1186/s12915-019-0628-6 30683096 PMC6347829

[86] Zhang YJ, Ioerger TR, Huttenhower C, Long JE, Sassetti CM, Sacchettini JC, Rubin EJ. 2012. Global assessment of genomic regions required for growth in Mycobacterium tuberculosis. PLOS Pathog 8:e1002946. doi:10.1371/journal.ppat.1002946 23028335 PMC3460630

[87] Bosch B, DeJesus MA, Poulton NC, Zhang W, Engelhart CA, Zaveri A, Lavalette S, Ruecker N, Trujillo C, Wallach JB, Li S, Ehrt S, Chait BT, Schnappinger D, Rock JM. 2021. Genome-wide gene expression tuning reveals diverse vulnerabilities of M. tuberculosis. Cell 184:4579–4592. doi:10.1016/j.cell.2021.06.033 34297925 PMC8382161

[88] Modlin SJ, Conkle-Gutierrez D, Kim C, Mitchell SN, Morrissey C, Weinrick BC, Jacobs WR, Ramirez-Busby SM, Hoffner SE, Valafar F. 2020. Drivers and sites of diversity in the DNA adenine methylomes of 93 Mycobacterium tuberculosis complex clinical isolates. Elife 9:e58542. doi:10.7554/eLife.58542 33107429 PMC7591249

[89] Rustad TR, Harrell MI, Liao R, Sherman DR. 2008. The enduring hypoxic response of Mycobacterium tuberculosis. PLoS One 3:e1502. doi:10.1371/journal.pone.0001502 18231589 PMC2198943

[90] Ates LS, Dippenaar A, Ummels R, Piersma SR, van der Woude AD, van der Kuij K, Le Chevalier F, Mata-Espinosa D, Barrios-Payán J, Marquina-Castillo B, Guapillo C, Jiménez CR, Pain A, Houben ENG, Warren RM, Brosch R, Hernández-Pando R, Bitter W. 2018. Mutations in ppe38 block PE_PGRS secretion and increase virulence of Mycobacterium tuberculosis. Nat Microbiol 3:181–188. doi:10.1038/s41564-017-0090-6 29335553

[91] Dong D, Wang D, Li M, Wang H, Yu J, Wang C, Liu J, Gao Q. 2012. PPE38 modulates the innate immune response and is required for Mycobacterium marinum virulence. Infect Immun 80:43–54. doi:10.1128/IAI.05249-11 22038915 PMC3255678

[92] Weerdenburg EM, Abdallah AM, Mitra S, de Punder K, van der Wel NN, Bird S, Appelmelk BJ, Bitter W, van der Sar AM. 2012. ESX-5-deficient Mycobacterium marinum is hypervirulent in adult zebrafish. Cell Microbiol 14:728–739. doi:10.1111/j.1462-5822.2012.01755.x 22256857

[93] Ates LS. 2020. New insights into the mycobacterial PE and PPE proteins provide a framework for future research. Mol Microbiol 113:4–21. doi:10.1111/mmi.14409 31661176 PMC7028111

[94] Qian J, Chen R, Wang H, Zhang X. 2020. Role of the PE/PPE family in host-pathogen interactions and prospects for anti-tuberculosis vaccine and diagnostic tool design. Front Cell Infect Microbiol 10:594288. doi:10.3389/fcimb.2020.594288 33324577 PMC7726347

[95] Sampson SL. 2011. Mycobacterial PE/PPE proteins at the host-pathogen interface. Clin Dev Immunol 2011:497203. doi:10.1155/2011/497203 21318182 PMC3034920

[96] De Maio F, Berisio R, Manganelli R, Delogu G. 2020. PE_PGRS proteins of Mycobacterium tuberculosis: a specialized molecular task force at the forefront of host-pathogen interaction. Virulence 11:898–915. doi:10.1080/21505594.2020.1785815 32713249 PMC7550000

[97] Karboul A, Gey van Pittius NC, Namouchi A, Vincent V, Sola C, Rastogi N, Suffys P, Fabre M, Cataldi A, Huard RC, Kurepina N, Kreiswirth B, Ho JL, Gutierrez MC, Mardassi H. 2006. Insights into the evolutionary history of tubercle bacilli as disclosed by genetic rearrangements within a PE_PGRS duplicated gene pair. BMC Evol Biol 6:107. doi:10.1186/1471-2148-6-107 17163995 PMC1762029

[98] Uplekar S, Heym B, Friocourt V, Rougemont J, Cole ST. 2011. Comparative genomics of Esx genes from clinical isolates of Mycobacterium tuberculosis provides evidence for gene conversion and epitope variation. Infect Immun 79:4042–4049. doi:10.1128/IAI.05344-11 21807910 PMC3187259

[99] Fishbein S, van Wyk N, Warren RM, Sampson SL. 2015. Phylogeny to function: PE/PPE protein evolution and impact on Mycobacterium tuberculosis pathogenicity. Mol Microbiol 96:901–916. doi:10.1111/mmi.12981 25727695

[100] Gómez-González PJ, Grabowska AD, Tientcheu LD, Tsolaki AG, Hibberd ML, Campino S, Phelan JE, Clark TG. 2023. Functional genetic variation in pe/ppe genes contributes to diversity in Mycobacterium tuberculosis lineages and potential interactions with the human host. Front Microbiol 14:1244319. doi:10.3389/fmicb.2023.1244319 37876785 PMC10591178

[101] Shannon CE. 1948. A mathematical theory of communication. Bell Syst Tech J 27:379–423. doi:10.1002/j.1538-7305.1948.tb01338.x

[102] Simpson EH. 1949. Measurement of diversity. Nature 163:688–688. doi:10.1038/163688a0

[103] Goodhead I, Darby AC. 2015. Taking the pseudo out of pseudogenes. Curr Opin Microbiol 23:102–109. doi:10.1016/j.mib.2014.11.012 25461580

[104] Boritsch EC, Frigui W, Cascioferro A, Malaga W, Etienne G, Laval F, Pawlik A, Le Chevalier F, Orgeur M, Ma L, Bouchier C, Stinear TP, Supply P, Majlessi L, Daffé M, Guilhot C, Brosch R. 2016. Pks5-recombination-mediated surface remodelling in Mycobacterium tuberculosis emergence. Nat Microbiol 1:15019. doi:10.1038/nmicrobiol.2015.19 27571976

[105] Bogart S. 2022. SankeyMATIC. Available from: https://sankeymatic.com/. Retrieved 19 Dec 2022.

[106] Jumper J, Evans R, Pritzel A, Green T, Figurnov M, Ronneberger O, Tunyasuvunakool K, Bates R, Žídek A, Potapenko A, et al.. 2021. Highly accurate protein structure prediction with AlphaFold. Nature 596:583–589. doi:10.1038/s41586-021-03819-2 34265844 PMC8371605

[107] Varadi M, Anyango S, Deshpande M, Nair S, Natassia C, Yordanova G, Yuan D, Stroe O, Wood G, Laydon A, et al.. 2022. AlphaFold protein structure database: massively expanding the structural coverage of protein-sequence space with high-accuracy models. Nucleic Acids Res 50:D439–D444. doi:10.1093/nar/gkab1061 34791371 PMC8728224

[108] Korlach J. 2015. Understanding accuracy in SMRT sequencing.

[109] Chakraborty P, Bajeli S, Kaushal D, Radotra BD, Kumar A. 2021. Biofilm formation in the lung contributes to virulence and drug tolerance of Mycobacterium tuberculosis. Nat Commun 12:1606. doi:10.1038/s41467-021-21748-6 33707445 PMC7952908

[110] Elghraoui A, Valafar F. 2022. MVP: detection of motif-making and -breaking mutations. arXiv

[111] Chauhan R, Ravi J, Datta P, Chen T, Schnappinger D, Bassler KE, Balázsi G, Gennaro ML. 2016. Reconstruction and topological characterization of the sigma factor regulatory network of Mycobacterium tuberculosis. Nat Commun 7:11062. doi:10.1038/ncomms11062 27029515 PMC4821874

[112] Mallick Gupta A, Mandal S. 2020. The C-terminal domain of M. tuberculosis ECF sigma factor I (SigI) interferes in SigI-RNAP interaction. J Mol Model 26:77. doi:10.1007/s00894-020-4322-y 32180013

[113] Ferrés I, Iraola G. 2021. An object-oriented framework for evolutionary pangenome analysis. Cell Reports Methods 1:100085. doi:10.1016/j.crmeth.2021.100085 35474671 PMC9017228

[114] Tettelin H, Riley D, Cattuto C, Medini D. 2008. Comparative genomics: the bacterial pan-genome. Curr Opin Microbiol 11:472–477. doi:10.1016/j.mib.2008.09.006 19086349

[115] Barrick JE, Colburn G, Deatherage DE, Traverse CC, Strand MD, Borges JJ, Knoester DB, Reba A, Meyer AG. 2014. Identifying structural variation in haploid microbial genomes from short-read resequencing data using breseq. BMC Genomics 15:1039. doi:10.1186/1471-2164-15-1039 25432719 PMC4300727

[116] Zhang H, Jain C, Aluru S. 2020. A comprehensive evaluation of long read error correction methods. BMC Genomics 21:889. doi:10.1186/s12864-020-07227-0 33349243 PMC7751105

[117] Meehan CJ, Goig GA, Kohl TA, Verboven L, Dippenaar A, Ezewudo M, Farhat MR, Guthrie JL, Laukens K, Miotto P, et al.. 2019. Whole genome sequencing of Mycobacterium tuberculosis: current standards and open issues. Nat Rev Microbiol 17:533–545. doi:10.1038/s41579-019-0214-5 31209399

[118] Mikheecheva NE, Zaychikova MV, Melerzanov AV, Danilenko VN. 2017. A nonsynonymous SNP catalog of Mycobacterium tuberculosis virulence genes and its use for detecting new potentially virulent sublineages. Genome Biol Evol 9:887–899. doi:10.1093/gbe/evx053 28338924 PMC5381574

[119] Casali N, Broda A, Harris SR, Parkhill J, Brown T, Drobniewski F. 2016. Whole genome sequence analysis of a large isoniazid-resistant tuberculosis outbreak in london: a retrospective observational study. PLOS Med 13:e1002137. doi:10.1371/journal.pmed.1002137 27701423 PMC5049847

[120] Farhat MR, Shapiro BJ, Sheppard SK, Colijn C, Murray M. 2014. A phylogeny-based sampling strategy and power calculator informs genome-wide associations study design for microbial pathogens. Genome Med 6:101. doi:10.1186/s13073-014-0101-7 25484920 PMC4256898

[121] Alonso H, Samper S, Martín C, Otal I. 2013. Mapping IS6110 in high-copy number Mycobacterium tuberculosis strains shows specific insertion points in the Beijing genotype. BMC Genomics 14:422. doi:10.1186/1471-2164-14-422 23800083 PMC3701491

[122] Bansal K, Sinha AY, Ghorpade DS, Togarsimalemath SK, Patil SA, Kaveri SV, Balaji KN, Bayry J. 2010. Src homology 3-interacting domain of Rv1917c of Mycobacterium tuberculosis induces selective maturation of human dendritic cells by regulating PI3K-MAPK-NF-kappaB signaling and drives Th2 immune responses. J Biol Chem 285:36511–36522. doi:10.1074/jbc.M110.158055 20837474 PMC2978579

[123] Damen MPM, Phan TH, Ummels R, Rubio-Canalejas A, Bitter W, Houben ENG. 2020. Modification of a PE/PPE substrate pair reroutes an ESX substrate pair from the mycobacterial ESX-1 type VII secretion system to the ESX-5 system. J Biol Chem 295:5960–5969. doi:10.1074/jbc.RA119.011682 32184351 PMC7196631

[124] Ehtram A, Shariq M, Ali S, Quadir N, Sheikh JA, Ahmad F, Sharma T, Ehtesham NZ, Hasnain SE. 2021. Teleological cooption of Mycobacterium tuberculosis PE/PPE proteins as porins: Role in molecular immigration and emigration. Int J Med Microbiol 311:151495. doi:10.1016/j.ijmm.2021.151495 33730677

[125] Namouchi A, Karboul A, Fabre M, Gutierrez MC, Mardassi H. 2013. Evolution of smooth tubercle Bacilli PE and PE_PGRS genes: evidence for a prominent role of recombination and imprint of positive selection. PLoS One 8:e64718. doi:10.1371/journal.pone.0064718 23705005 PMC3660525

[126] Nain Z, Karim MM, Sen MK, Adhikari UK. 2020. Structural basis and designing of peptide vaccine using PE-PGRS family protein of Mycobacterium ulcerans-An integrated vaccinomics approach. Mol Immunol 120:146–163. doi:10.1016/j.molimm.2020.02.009 32126449

[127] Samue B, Volkmann T, Cornelius S, Mukhopadhay S, MejoJose, Mitra K, Kumar AMV, Oeltmann JE, Parija S, Prabhakaran AO, Moonan PK, Chadha VK. 2016. Relationship between nutritional support and tuberculosis treatment outcomes in West Bengal, India. JTR 04:213–219. doi:10.4236/jtr.2016.44023 PMC5201187 28042591

[128] Campuzano J, Aguilar D, Arriaga K, León JC, Salas-Rangel LP, González-y-Merchand J, Hernández-Pando R, Espitia C. 2007. The PGRS domain of Mycobacterium tuberculosis. Vaccine (Auckl) 25:3722–3729. doi:10.1016/j.vaccine.2006.12.042 17399860

[129] Monterrubio-López GP, González-Y-Merchand JA, Ribas-Aparicio RM. 2015. Identification of novel potential vaccine candidates against tuberculosis based on reverse vaccinology. Biomed Res Int 2015:483150. doi:10.1155/2015/483150 25961021 PMC4413515

[130] Brennan MJ. 2017. The enigmatic PE/PPE multigene family of mycobacteria and tuberculosis vaccination. Infect Immun 85:e00969-16. doi:10.1128/IAI.00969-16 28348055 PMC5442627

[131] Zvi A, Ariel N, Fulkerson J, Sadoff JC, Shafferman A. 2008. Whole genome identification of Mycobacterium tuberculosis vaccine candidates by comprehensive data mining and bioinformatic analyses. BMC Med Genomics 1:18. doi:10.1186/1755-8794-1-18 18505592 PMC2442614

[132] Wang Y, Li Z, Wu S, Fleming J, Li C, Zhu G, Chen B, Ren B, Wang X, Du B, Li P, Hu P, Yang J, Liu Y, Zhou C, Zhang X-E, Bi L, Zhang H, Yang J, Zhang Z. 2021. Systematic evaluation of Mycobacterium tuberculosis proteins for antigenic properties identifies Rv1485 and Rv1705c as potential protective subunit vaccine candidates. Infect Immun 89:e00585-20. doi:10.1128/IAI.00585-20 33318140 PMC8097267

[133] Jiao J, Zheng N, Wei W, Fleming J, Wang X, Li Z, Zhang L, Liu Y, Zhang Z, Shen A, Chuanyou L, Bi L, Zhang H. 2021. M. tuberculosis CRISPR/Cas proteins are secreted virulence factors that trigger cellular immune responses. Virulence 12:3032–3044. doi:10.1080/21505594.2021.2007621 34886764 PMC8667911

[134] Assis PA, Espíndola MS, Paula-Silva FWG, Rios WM, Pereira PAT, Leão SC, Silva CL, Faccioli LH. 2014. Mycobacterium tuberculosis expressing phospholipase C subverts PGE2 synthesis and induces necrosis in alveolar macrophages. BMC Microbiol 14:128. doi:10.1186/1471-2180-14-128 24886263 PMC4057917

[135] Le Chevalier F, Cascioferro A, Frigui W, Pawlik A, Boritsch EC, Bottai D, Majlessi L, Herrmann JL, Brosch R. 2015. Revisiting the role of phospholipases C in virulence and the lifecycle of Mycobacterium tuberculosis. Sci Rep 5:16918. doi:10.1038/srep16918 26603639 PMC4658479

[136] Freidlin PJ, Nissan I, Luria A, Goldblatt D, Schaffer L, Kaidar-Shwartz H, Chemtob D, Dveyrin Z, Head SR, Rorman E. 2017. Structure and variation of CRISPR and CRISPR-flanking regions in deleted-direct repeat region Mycobacterium tuberculosis complex strains. BMC Genomics 18:168. doi:10.1186/s12864-017-3560-6 28201993 PMC5310062

[137] Comín J, Cebollada A, Ibarz D, Viñuelas J, Sahagún J, Torres L, Iglesias M-J, Samper S. 2022. Analysis of the twenty-six largest outbreaks of tuberculosis in Aragon using whole-genome sequencing for surveillance purposes. Sci Rep 12:18766. doi:10.1038/s41598-022-23343-1 36335223 PMC9637126

[138] Sarmiento ME, Alvarez N, Chin KL, Bigi F, Tirado Y, García MA, Anis FZ, Norazmi MN, Acosta A. 2019. Tuberculosis vaccine candidates based on mycobacterial cell envelope components. Tuberculosis (Edinb) 115:26–41. doi:10.1016/j.tube.2019.01.003 30948174

[139] Yamamoto K, Nakata N, Mukai T, Kawagishi I, Ato M. 2021. Coexpression of MmpS5 and MmpL5 contributes to both efflux transporter MmpL5 trimerization and drug resistance in Mycobacterium tuberculosis. mSphere 6:e00518-20. doi:10.1128/mSphere.00518-20 33408221 PMC7845600

[140] Kanji A, Hasan R, Hasan Z. 2019. Efflux pump as alternate mechanism for drug resistance in Mycobacterium tuberculosis. Indian J Tuberc 66:20–25. doi:10.1016/j.ijtb.2018.07.008 30797276

[141] Milano A, Pasca MR, Provvedi R, Lucarelli AP, Manina G, Luisa de Jesus Lopes Ribeiro A, Manganelli R, Riccardi G. 2009. Azole resistance in Mycobacterium tuberculosis is mediated by the MmpS5–MmpL5 efflux system. Tuberculosis (Edinb) 89:84–90. doi:10.1016/j.tube.2008.08.003 18851927

[142] Vargas R, Freschi L, Spitaleri A, Tahseen S, Barilar I, Niemann S, Miotto P, Cirillo DM, Köser CU, Farhat MR. 2021. Role of epistasis in amikacin, kanamycin, bedaquiline, and clofazimine resistance in Mycobacterium tuberculosis complex. Antimicrob Agents Chemother 65:e01164-21. doi:10.1128/AAC.01164-21 34460306 PMC8522733

[143] Sandhu P, Akhter Y. 2015. The internal gene duplication and interrupted coding sequences in the MmpL genes of Mycobacterium tuberculosis: Towards understanding the multidrug transport in an evolutionary perspective. Int J Med Microbiol 305:413–423. doi:10.1016/j.ijmm.2015.03.005 25841626

[144] Laws M, Jin P, Rahman KM. 2022. Efflux pumps in Mycobacterium tuberculosis and their inhibition to tackle antimicrobial resistance. Trends Microbiol 30:57–68. doi:10.1016/j.tim.2021.05.001 34052094

[145] Sun Q, Xiao T-Y, Liu H-C, Zhao X-Q, Liu Z-G, Li Y-N, Zeng H, Zhao L-L, Wan K-L. 2018. Mutations within embCAB Are associated with variable level of ethambutol resistance in Mycobacterium tuberculosis Isolates from China. Antimicrob Agents Chemother 62:e01279-17. doi:10.1128/AAC.01279-17 29084750 PMC5740351

[146] Sun Q, Zou Y, Feng Q, Gong Z, Song M, Li M, Chen Z. 2023. The acetylation of pknH is linked to the ethambutol resistance of Mycobacterium tuberculosis. Arch Microbiol 205:337. doi:10.1007/s00203-023-03676-9 37740776

[147] Kumar S, Khan MZ, Khandelwal N, Chongtham C, Singha B, Dabla A, Behera D, Singh A, Gopal B, Arimbasseri GA, Kamat SS, Nandicoori VK. 2022. Mycobacterium tuberculosis transcription factor embr regulates the expression of key virulence factors that aid in ex vivo and in vivo survival. mBio 13:e03836-21. doi:10.1128/mbio.03836-21 35471080 PMC9239209

[148] Nasiri MJ, Haeili M, Ghazi M, Goudarzi H, Pormohammad A, Imani Fooladi AA, Feizabadi MM. 2017. New insights in to the intrinsic and acquired drug resistance mechanisms in mycobacteria. Front Microbiol 8:681. doi:10.3389/fmicb.2017.00681 28487675 PMC5403904

[149] Wang W-F, Lu M-YJ, Cheng T-JR, Tang Y-C, Teng Y-C, Hwa T-Y, Chen Y-H, Li M-Y, Wu M-H, Chuang P-C, Jou R, Wong C-H, Li W-H. 2020. Genomic analysis of Mycobacterium tuberculosis isolates and construction of a beijing lineage reference genome. Genome Biol Evol 12:3890–3905. doi:10.1093/gbe/evaa009 31971587 PMC7058165

[150] McEvoy CRE, Falmer AA, Gey van Pittius NC, Victor TC, van Helden PD, Warren RM. 2007. The role of IS6110 in the evolution of Mycobacterium tuberculosis. Tuberculosis (Edinb) 87:393–404. doi:10.1016/j.tube.2007.05.010 17627889

[151] Hillery N, Groessl EJ, Trollip A, Catanzaro D, Jackson L, Rodwell TC, Garfein RS, Lin S-YG, Eisenach K, Ganiats TG, Park D, Valafar F, Rodrigues C, Crudu V, Victor TC, Catanzaro A. 2014. The global consortium for drug-resistant tuberculosis diagnostics (GCDD): design of a multi-site, head-to-head study of three rapid tests to detect extensively drug-resistant tuberculosis. Trials 15:434. doi:10.1186/1745-6215-15-434 25377177 PMC4232628

[152] Verboven L, Phelan J, Heupink TH, Van Rie A. 2022. TBProfiler for automated calling of the association with drug resistance of variants in Mycobacterium tuberculosis. PLoS One 17:e0279644. doi:10.1371/journal.pone.0279644 36584023 PMC9803136

[153] Seemann T. 2014. Prokka: rapid prokaryotic genome annotation. Bioinformatics 30:2068–2069. doi:10.1093/bioinformatics/btu153 24642063

[154] Modlin SJ, Elghraoui A, Gunasekaran D, Zlotnicki AM, Dillon NA, Dhillon N, Kuo N, Robinhold C, Chan CK, Baughn AD, Valafar F. 2021. Structure-aware Mycobacterium tuberculosis functional annotation uncloaks resistance, metabolic, and virulence genes. mSystems 6:e00673-21. doi:10.1128/mSystems.00673-21 34726489 PMC8562490

[155] Rustad TR, Minch KJ, Ma S, Winkler JK, Hobbs S, Hickey M, Brabant W, Turkarslan S, Price ND, Baliga NS, Sherman DR. 2014. Mapping and manipulating the Mycobacterium tuberculosis transcriptome using a transcription factor overexpression-derived regulatory network. Genome Biol 15:502. doi:10.1186/PREACCEPT-1701638048134699 25380655 PMC4249609

[156] Bellerose MM, Proulx MK, Smith CM, Baker RE, Ioerger TR, Sassetti CM. 2020. Distinct bacterial pathways influence the efficacy of antibiotics against Mycobacterium tuberculosis. mSystems 5:e00396-20. doi:10.1128/mSystems.00396-20 32753506 PMC7406225

[157] Judd JA, Canestrari J, Clark R, Joseph A, Lapierre P, Lasek-Nesselquist E, Mir M, Palumbo M, Smith C, Stone M, et al.. 2021. A mycobacterial systems resource for the research community. mBio 12:e02401-20. doi:10.1128/mBio.02401-20 33653882 PMC8092266

[158] Blighe KLA. 2019. PCAtools:everything principal component analysis.

[159] Mi H, Muruganujan A, Ebert D, Huang X, Thomas PD. 2019. PANTHER version 14: more genomes, a new PANTHER GO-slim and improvements in enrichment analysis tools. Nucleic Acids Res 47:D419–D426. doi:10.1093/nar/gky1038 30407594 PMC6323939

[160] Camacho C, Coulouris G, Avagyan V, Ma N, Papadopoulos J, Bealer K, Madden TL. 2009. BLAST+: architecture and applications. BMC Bioinformatics 10:421. doi:10.1186/1471-2105-10-421 20003500 PMC2803857

[161] Lees JA, Galardini M, Bentley SD, Weiser JN, Corander J. 2018. Pyseer: a comprehensive tool for microbial pangenome-wide association studies. Bioinformatics 34:4310–4312. doi:10.1093/bioinformatics/bty539 30535304 PMC6289128

[162] Foundation TR. 2021. The R project for statistical computing R.

[163] Shannon P, Markiel A, Ozier O, Baliga NS, Wang JT, Ramage D, Amin N, Schwikowski B, Ideker T. 2003. Cytoscape: a software environment for integrated models of biomolecular interaction networks. Genome Res 13:2498–2504. doi:10.1101/gr.1239303 14597658 PMC403769

[164] Smoot M, Albrecht M, Assenov Y. 2021. NetworkAnalyzer.

[165] Van Rossum G. 2009. Python 3 reference manual. Scotts Valley, CA CreateSpace

