# Supplementary material for: Interred mechanisms of resistance and host immune evasion revealed through network-connectivity analysis of M. tuberculosis complex graph pangenome
Source: mSystems. 2025 Mar 6;10(4):e00499-24. doi: 10.1128/msystems.00499-24 (PMC12013269; doi:10.1128/msystems.00499-24)
Supplement: Supplemental material — Supplemental figures and tables. [file msystems.00499-24-s0008.docx]

Contents

[Table S1. PPE/PE/PGRS/ESX Non-merged Annotation Genes in the Core & Accessory Genomes 2](#_Toc146476871)

[Table S2. Predicted function of *ab initio* predicted “group” genes by core and accessory in the pangenome of *M. tuberculosis* isolates (N = 109 isolates) † 3](#_Toc146476872)

[Table S3. BLASTN Alignment of Pangenome-derived Unnamed *Ab Initio* Predicted Genes to H37Rv 3](#_Toc146476873)

[Table S4. Distribution of duplicated genes in core and accessory genome by lineage 1-5 4](#_Toc146476874)

[Table S5. Table of results from structural-gwa fixed effects model with population structure 6](#_Toc146476875)

[Table S6. Panaroo output structural variants only found in lineage 1 isolates. 9](#_Toc146476876)

[Table S7. Panaroo output structural variants only found in lineage 2 isolates. 11](#_Toc146476877)

[Table S8. Panaroo output structural variants only found in lineage 3 isolates. 15](#_Toc146476878)

[Table S9. Panaroo output structural variants only found in lineage 4 isolates, not including H37Rv. 16](#_Toc146476879)

[Table S10. Panaroo output structural variants only found in lineage 4 isolates, including H37Rv. 20](#_Toc146476880)

[Table S11. Table of global network characteristics as determined by Cytoscape network analyzer (N = 109 isolates) on Panaroo output gene adjacency graph 21](#_Toc146476881)

[Table S12. Gene list for high structural variant clusters along pangenome network 21](#_Toc146476882)

[Table S13. Table of Counts Of Isolates By Drug Resistance Phenotype (resistant:R, susceptible:S, Not Determined) across Antibiotics 30](#_Toc146476883)

[Figure S1. Gene ontology functional enrichment analysis of core genome from our pangenome analysis. 31](#_Toc146476884)

[Figure S2. Correlation plot and loadings plot from the principal component analysis of the pangenome 32](#_Toc146476885)

[Figure S3. Randomized permutation test with replacement 33](#_Toc146476886)

[Figure S4. Lineage-specific pangenome Sankey diagrams 34](#_Toc146476887)

[Figure S5. Average copy number of genes with duplicates in the lineage-specific pangenomes. 35](#_Toc146476888)

[Figure S6. Distribution of the number of isolates with a given fragmented gene 36](#_Toc146476889)

[Figure S7. Distribution of fragmented genes per genome by lineage 37](#_Toc146476890)

[Figure S8. Panaroo merged annotations among two genes. 38](#_Toc146476891)

[Figure S9. Hierarchical clustering of high structural variant region genes parsed by high structural variant cluster and lineage. 39](#_Toc146476892)

[Figure S10. Overviews of lineage-specific networks composed of gene contributors to structural variants found by Panaroo. 40](#_Toc146476893)

[Figure S11. Scatterplot of sample size and core genome size reported by other studies and this study, 41](#_Toc146476894)

[Figure S12. Quality of 109 M. tuberculosis clinical isolates de novo assemblies as measured by numbers of SNPs and single-base insertions and deletions with respect to H37Rv 42](#_Toc146476895)

[Supplemental Methods 43](#_Toc146476896)

[Genome Assembly with Quality Control 43](#_Toc146476897)

[1. *Assembly* 43](#_Toc146476898)

[2. *Circularization* 43](#_Toc146476899)

[3. *Consensus polishing* 43](#_Toc146476900)

[4. *Assembly Quality Control* 44](#_Toc146476901)

[References 44](#_Toc146476902)

# Table S1. PPE/PE/PGRS/ESX Non-merged Annotation Genes in the Core & Accessory Genomes±

| **Core Genome** | | | |
| --- | --- | --- | --- |
| **PPE**  **(N= 45)** | **PE**  **(N= 26)** | **PE_PGRS**  **(N= 45)** | **ESX**  **(N= 12)** |
| PPE41  PPE24  PPE54  PPE12  PPE18  PPE10  PPE35  PPE64  PPE44  PPE27  PPE33  PPE21  PPE49  PPE45  PPE68  PPE14  PPE13  PPE30  PPE28  PPE29  PPE51  PPE22  PPE69  PPE16  PPE31  PPE32  PPE52  PPE17  PPE63  PPE1  PPE62  PPE42  PPE61  PPE23  PPE4  PPE11  PPE20  PPE2  PPE53  PPE3  PPE65  PPE37  PPE43  PPE15  PPE36 | PE33  PE25  PE20  PE19  PE13  PE15  PE5  PE14  PE34  PE2  PE12  PE17  PE35  PE23  PE8  PE3  PE4  PE16  PE7  PE36  PE31  PE29  PE27  PE1  PE30  PE11 | PE_PGRS24  PE_PGRS53  PE_PGRS59  PE_PGRS45  PE_PGRS16  PE_PGRS22  PE_PGRS9  PE_PGRS18  PE_PGRS43  PE_PGRS5  PE_PGRS46  PE_PGRS34  PE_PGRS8  PE_PGRS61  PE_PGRS31  PE_PGRS52  PE_PGRS7  PE_PGRS42  PE_PGRS28  PE_PGRS6  PE_PGRS25  PE_PGRS38  PE_PGRS29  PE_PGRS33  PE_PGRS41  PE_PGRS27  PE_PGRS44  PE_PGRS32  PE_PGRS35  PE_PGRS62  PE_PGRS58  PE_PGRS47  PE_PGRS15  PE_PGRS23  PE_PGRS48  PE_PGRS1  PE_PGRS11  PE_PGRS39  PE_PGRS40  PE_PGRS17  PE_PGRS21  PE_PGRS10  PE_PGRS51  PE_PGRS2  PE26 | esxA  esxH  esxG  esxB  esxT  esxF  esxD  esxU  esxC  esxE  esxJ  esxQ |
| **Accessory Genome** | | | |
| **PPE**  **(N= 16)** | **PE**  **(N= 6)** | **PE_PGRS**  **(N= 8)** | **ESX**  **(N= 7)** |
| PPE25  PPE26  PPE55  PPE40  PPE39  PPE38  PPE58  PPE59  PPE34  PPE16  PPE47  PPE8  PPE46  PPE42  PPE28  PPE36 | PE22  PE18  PE32  PE25  PE27A  PE6 | PE_PGRS26 PE_PGRS54 PE_PGRS4 PE_PGRS57 PE_PGRS30 PE_PGRS28 PE_PGRS19  wag22 | esxL  esxN  esxJ  esxS  esxR  esxP  esxO |

**±** Gene counts are presented as the number of unique genes in the respective gene family present in the pangenome, and thus do not include multiple gene copies in the sums.

# Table S2. Predicted function of *ab initio* predicted “group” genes by core and accessory in the pangenome of *M. tuberculosis* isolates (N = 109 isolates) †

|  | **Total Number of Genes=4325** | | |
| --- | --- | --- | --- |
|  | **Total**  **N (%)** | **Core**  **N (%)** | **Accessory**  **N (%)** |
| Total unnamed putative novel genes† | 290 (6.71%) | 130 (44.8%) | 160 (55.2%) |
| Hypothesized function of "group” genes  Hypothetical protein  Hypothetical protein;putative proteinA/RVBD_2386A  Putative antitoxin VapB51  Putative membrane protein  Putative protein A  Putative protein B  ISNCY family transposase ISBli16 | 279 (96.2%)  1 (0.3%)  1 (0.3%)  1 (0.3%)  6 (2.1%)  1 (0.3%)  1 (0.3%) | 121 (43%)  1 (100%)  1 (100%)  0 (0%)  5 (83%)  1 (100%)  1 (100%) | 158 (57%)  0 (0%)  0 (0%)  1 (100%)  1 (17%)  0 (0%)  0 (0%) |

†No unnamed putative novel genes were found in H37Rv using H37Rv-NCBI GFF3 file

# Table S3. BLASTN Alignment of Pangenome-derived Unnamed *Ab Initio* Predicted Genes to H37Rv

| **Panaroo Gene** | **Gene Aligned in H37Rv** | **% Identity** | **Alignment Length (bp)** | **Mismatch** | **Gap** | **E value** | **Bit Score** | **Length in H37Rv (bp)** | **Length in Pangenome (bp)** | **No. Isolates** | **% Coverage** |
| --- | --- | --- | --- | --- | --- | --- | --- | --- | --- | --- | --- |
| group_11 | *Rv0689c* | 100 | 240 | 0 | 0 | 1.04E-129 | 444 | 255 | 249 | 109 | 94.12 |
| group_186 | *Rv3611* | 100 | 586 | 0 | 0 | 0 | 1083 | 654 | 1245 | 97 | 89.60 |
| group_192 | *Rv2644c* | 100 | 300 | 0 | 0 | 7.21E-163 | 555 | 318 | 309 | 107 | 94.34 |
| group_21 | *Rv1545* | 100 | 189 | 0 | 0 | 1.95E-101 | 350 | 228 | 234 | 109 | 82.89 |
| group_266 | *fadD18* | 100 | 615 | 0 | 0 | 0 | 1136 | 657 | 1002 | 2 | 93.61 |
| group_28 | *Rv3224B* | 100 | 219 | 0 | 0 | 3.84E-118 | 405 | 219 | 228 | 109 | 100.00 |
| group_328 | *Rv3861* | 100 | 327 | 0 | 0 | 2.02E-177 | 604 | 327 | 847 | 2 | 100.00 |
| group_341 | *Rv1874* | 100 | 610 | 0 | 0 | 0 | 1127 | 687 | 834 | 1 | 88.79 |
| group_343 | *Rv0699* | 99.55 | 222 | 1 | 0 | 1.31E-117 | 405 | 222 | 756 | 109 | 100.00 |
| group_357 | *pe6* | 100 | 516 | 0 | 0 | 0 | 953 | 516 | 612 | 109 | 100.00 |
| group_361 | *fadD18* | 100 | 603 | 0 | 0 | 0 | 1114 | 657 | 603 | 76 | 91.78 |
| group_414 | *Rv2998A* | 100 | 177 | 0 | 0 | 1.07E-94 | 327 | 204 | 303 | 109 | 86.76 |

# Table S4. Distribution of duplicated genes in core and accessory genome by lineage 1-5

| **All Copies in Core** | **Core Copy + Accessory Cop(ies)** | **All Copies in Accessory** |
| --- | --- | --- |
| *Lineage 1* | | |
| PE1  Rv0036/0515  mgtE  mmpS1  mmpL5  tuf  sseC1/2  cysA2/3  Rv1042c/1149  IS1081  IS1557  Rv2082  Rv2807  IS1608  amiD  espI | lppB;lppA  PPE59;PPE57  bioF2  IS6110(1)  pknD  kdpD  esxJ  embR  p27  PE_PGRS28  pks5  Rv1723  PPE28  Rv1989c  PE25  Rv2735c  Rv3900c | Rv0094c/3467  pks6  IS6110(2)  PPE34  plcA  PPE38  PE_PGRS57 |
| *Lineage 2* | | |
| PE1  Rv0036/0515  mgtE  sseC1/2  cysA2/3  Rv1042c/1149  IS1081  IS1557  Rv2082  Rv2807  IS1608  amiD  espI | PPE59;PPE57  Rv0094c/3467  tuf  IS6110(2)  IS6110(1)  esxJ  PPE16  glgC  embR  Rv1319c  moeY  Rv1358  Rv1371  erg3  gnd1  uspC  Rv2390c  PE25  Rv3190c  Rv3635  embC  embA  embB  Rv3796  fadE35  accD4  pks13  mycP2 | Rv1879  PPE34  PPE38  PE_PGRS57 |
| *Lineage 3* | | |
| PE1  Rv0036/0515  mgtE  Rv0395  sseC1/2  cysA2/3  Rv1042c/1149  IS1081  IS1557  p27  Rv2807  IS1608  amiD  espI | PPE59;PPE57  bioF2  Rv0221  mmpL5  tuf  IS6110(2)  IS6110(1)  esxJ  embR  PPE34  erm(37)  Rv2082  PPE38  PE25  Rv2435c  Rv2735c  ald  mycP2 | cut1  Rv2819c |
| *Lineage 4* | | |
| Rv0036/0515  mgtE  sseC1/2  cysA2/3  IS1081  IS1557 | PPE59;PPE57  bioF2  fadD34  Rv0395  mmpL1  pks6  mmpL5  IS6110(1)  Rv0963c  kdpD  esxJ  Rv1042c/1149  PPE16  lpqY  embR  Rv1358  Rv1371  pks5  treX  Rv1871c  lldD2  Rv1873  PPE34  p27  PPE38  PE25  PPE42  Rv2625c  Rv2807  Rv3113  icd1  IS1608  amiD  espI  mycP2 | lppB;lppA  Rv0094c/3467  PE1  IS6110(2)  Rv1319c  moeY  cut1  Rv1762c  esxN  Rv2082  esxR  PPE55  Rv3900c |
| *Lineage 5* | | |
| PPE59;PPE57  bioF2  PE1  Rv0036/0515  mgtE  mmpS1  sseC1/2  cysA2/3  pknD  Rv0963c  esxJ  Rv1042c/1149  IS1081  embR  IS1557  Rv2082  PE25  Rv2807  IS1608  amiD  espI | gmhA  mmpL5  IS6110(2)  IS6110(1)  pks5 | Rv0094c/3467  cut1 |

Table S5. Table of results from structural-gwa fixed effects model with population structure **correction,** adjusted for multiple hypothesis testing via false discovery rate, by antibiotic tested. Results are all population structure adjusted P-values above 0.05 with odds ratio >1.

|  | af | filter.pvalue | lrt.pvalue | beta | beta.std.err | intercept | odds.ratio | conf.int95_lower | conf.int95_upper | drug | fdr.adj |
| --- | --- | --- | --- | --- | --- | --- | --- | --- | --- | --- | --- |
| Rv1927-mpt63-fadD31 | 0.906 | 0.935 | 0.00427 | 5.01 | 1.9 | -3.19 | 149.9047361 | 146.1997361 | 153.6097361 | INH | 1 |
| group_192-Rv2645-Rv2646 | 0.83 | 0.0000359 | 0.00331 | 4.97 | 2.01 | -2.91 | 144.0268874 | 140.1073874 | 147.9463874 | INH | 1 |
| Rv2645-group_192-arsC | 0.83 | 0.0000359 | 0.00331 | 4.97 | 2.01 | -2.91 | 144.0268874 | 140.1073874 | 147.9463874 | INH | 1 |
| PPE35-PPE34-group_337 | 0.113 | 0.626 | 0.00591 | 4.92 | 2.48 | 0.836 | 137.0026132 | 132.1666132 | 141.8386132 | INH | 1 |
| Rv0756c-Rv0755A-group_289 | 0.0755 | 0.697 | 0.0238 | 4.72 | 2.9 | 0.971 | 112.1682527 | 106.5132527 | 117.8232527 | INH | 1 |
| group_109-group_289-Rv0755A | 0.0755 | 0.697 | 0.0238 | 4.72 | 2.9 | 0.971 | 112.1682527 | 106.5132527 | 117.8232527 | INH | 1 |
| tsaE-alr-PPE59~~~PPE57 | 0.16 | 0.867 | 0.00524 | 4.46 | 1.69 | 0.933 | 86.4875091 | 83.1920091 | 89.7830091 | INH | 1 |
| Rv3430c-PPE59~~~PPE57-alr | 0.16 | 0.867 | 0.00524 | 4.46 | 1.69 | 0.933 | 86.4875091 | 83.1920091 | 89.7830091 | INH | 1 |
| group_268-btuD_1~~~Rv1747-pknH~~~pknD | 0.189 | 0.000282 | 0.0174 | 4.14 | 2.4 | 0.46 | 62.80282145 | 58.12282145 | 67.48282145 | INH | 1 |
| btuD_1~~~Rv1747-pknH~~~pknD-Rv1265 | 0.189 | 0.000282 | 0.0174 | 4.14 | 2.4 | 0.46 | 62.80282145 | 58.12282145 | 67.48282145 | INH | 1 |
| group_225-Rv3901c-group_376 | 0.151 | 0.0185 | 0.0472 | 4.1 | 2.95 | 0.599 | 60.3402876 | 54.5877876 | 66.0927876 | INH | 1 |
| Rv3837c-group_23-Rv3836 | 0.726 | 0.000000244 | 0.00492 | 4.05 | 1.58 | -1.27 | 57.39745705 | 54.31645705 | 60.47845705 | INH | 1 |
| group_23-Rv3836-Rv3835 | 0.726 | 0.000000244 | 0.00492 | 4.05 | 1.58 | -1.27 | 57.39745705 | 54.31645705 | 60.47845705 | INH | 1 |
| sodA-group_221-group_172 | 0.877 | 0.059 | 0.0201 | 3.82 | 2.14 | -1.97 | 45.60420832 | 41.43120832 | 49.77720832 | INH | 1 |
| group_303-group_217-group_312 | 0.66 | 0.00262 | 0.000125 | 3.77 | 1.17 | -0.665 | 43.38006484 | 41.09856484 | 45.66156484 | INH | 1 |
| Rv2083-group_312-group_217 | 0.66 | 0.00262 | 0.000125 | 3.77 | 1.17 | -0.665 | 43.38006484 | 41.09856484 | 45.66156484 | INH | 1 |
| Rv3845-group_172-group_221 | 0.887 | 0.134 | 0.0266 | 3.59 | 1.97 | -1.78 | 36.23407593 | 32.39257593 | 40.07557593 | INH | 1 |
| Rv1930c-hcaB~~~Rv1928c-Rv1927 | 0.811 | 0.0837 | 0.032 | 3.31 | 1.71 | -1.03 | 27.38512547 | 24.05062547 | 30.71962547 | INH | 1 |
| Rv2345-esxN~~~esxO~~~esxL-group_340 | 0.689 | 0.00948 | 0.0046 | 3 | 1.24 | -0.629 | 20.08553692 | 17.66753692 | 22.50353692 | INH | 1 |
| Rv2348c-group_340-esxN~~~esxO~~~esxL | 0.689 | 0.00948 | 0.0046 | 3 | 1.24 | -0.629 | 20.08553692 | 17.66753692 | 22.50353692 | INH | 1 |
| bioF2-Rv0030-Rv0029 | 0.17 | 0.0578 | 0.0203 | 2.98 | 1.4 | 0.753 | 19.68781664 | 16.95781664 | 22.41781664 | INH | 1 |
| acpA-group_283-bioF2 | 0.17 | 0.0578 | 0.0203 | 2.98 | 1.4 | 0.753 | 19.68781664 | 16.95781664 | 22.41781664 | INH | 1 |
| group_255-group_303-group_217 | 0.83 | 0.0000359 | 0.000482 | 2.96 | 0.934 | -0.871 | 19.29797176 | 17.47667176 | 21.11927176 | INH | 1 |
| Rv1761c-group_229-group_319 | 0.481 | 0.00279 | 0.00471 | 2.71 | 1.03 | 0.0617 | 15.02927551 | 13.02077551 | 17.03777551 | INH | 1 |
| group_235-group_317-PE27A | 0.34 | 0.427 | 0.00224 | 2.57 | 0.953 | 0.438 | 13.06582444 | 11.20747444 | 14.92417444 | INH | 1 |
| aceAb~~~icl2~~~aceAa-group_71-group_329 | 0.226 | 0.461 | 0.031 | 2.51 | 1.28 | 0.827 | 12.30493006 | 9.808930061 | 14.80093006 | INH | 1 |
| plcD~~~plcB-Rv1754c-PPE24 | 0.132 | 0.4 | 0.0378 | 2.41 | 1.29 | 1.06 | 11.13396115 | 8.618461145 | 13.64946115 | INH | 1 |
| PPE46~~~PPE47-PE27A-group_317 | 0.302 | 0.635 | 0.00384 | 2.3 | 0.899 | 0.656 | 9.974182455 | 8.221132455 | 11.72723245 | INH | 1 |
| vapC18-lppA~~~vapB18~~~lppB-lppA | 0.283 | 0.874 | 0.0114 | 2.28 | 0.981 | 0.556 | 9.77668041 | 7.86373041 | 11.68963041 | INH | 1 |
| Rv2542-lppA-lppA~~~vapB18~~~lppB | 0.283 | 0.874 | 0.0114 | 2.28 | 0.981 | 0.556 | 9.77668041 | 7.86373041 | 11.68963041 | INH | 1 |
| group_308-eccD2-Rv3888c | 0.896 | 0.0139 | 0.0221 | 2.09 | 0.967 | -0.581 | 8.084915164 | 6.199265164 | 9.970565164 | INH | 1 |
| eccE2-group_308-eccD2 | 0.896 | 0.0139 | 0.0221 | 2.09 | 0.967 | -0.581 | 8.084915164 | 6.199265164 | 9.970565164 | INH | 1 |
| group_229-group_319-Rv1765c~~~Rv2015c | 0.453 | 0.0107 | 0.0163 | 2.04 | 0.903 | 0.401 | 7.690609199 | 5.929759199 | 9.451459199 | INH | 1 |
| group_75-plcA-plcB | 0.858 | 0.00916 | 0.0244 | 2.03 | 0.955 | -0.31 | 7.614086359 | 5.751836359 | 9.476336359 | INH | 1 |
| moaC3-Rv3324A~~~moaB1-group_298 | 0.0943 | 0.935 | 0.0446 | 1.99 | 1.02 | 1.19 | 7.315533762 | 5.326533762 | 9.304533762 | INH | 1 |
| Rv3324A~~~moaB1-group_298-group_311 | 0.0943 | 0.935 | 0.0446 | 1.99 | 1.02 | 1.19 | 7.315533762 | 5.326533762 | 9.304533762 | INH | 1 |
| PPE50~~~PPE51-PPE51-Rv3136A | 0.717 | 0.00000684 | 0.0351 | 1.94 | 0.951 | -0.0415 | 6.958750971 | 5.104300971 | 8.813200971 | INH | 1 |
| devR-Rv3134c-PPE50~~~PPE51 | 0.717 | 0.00000684 | 0.0351 | 1.94 | 0.951 | -0.0415 | 6.958750971 | 5.104300971 | 8.813200971 | INH | 1 |
| esxS-group_235-group_317 | 0.453 | 0.981 | 0.0168 | 1.87 | 0.837 | 0.538 | 6.488296399 | 4.856146399 | 8.120446399 | INH | 1 |
| Rv1919c-PPE35-group_3292 | 0.314 | 0.395 | 0.011 | 3.8 | 1.82 | -0.338 | 44.70118449 | 41.15218449 | 48.25018449 | RIF | 1 |
| Rv3837c-group_23-Rv38362 | 0.733 | 0.00000587 | 0.0236 | 3.37 | 1.43 | -1.38 | 29.07852706 | 26.29002706 | 31.86702706 | RIF | 1 |
| group_23-Rv3836-Rv38352 | 0.733 | 0.00000587 | 0.0236 | 3.37 | 1.43 | -1.38 | 29.07852706 | 26.29002706 | 31.86702706 | RIF | 1 |
| Rv1927-mpt63-fadD311 | 0.905 | 0.668 | 0.0496 | 3.04 | 1.59 | -1.86 | 20.90524324 | 17.80474324 | 24.00574324 | RIF | 1 |
| rmlB-rmlC-Rv3466~~~Rv0095c~~~MTB00061 | 0.867 | 0.577 | 0.011 | 2.8 | 1.23 | -1.55 | 16.44464677 | 14.04614677 | 18.84314677 | RIF | 1 |
| mhpE-Rv3468c-Rv3466~~~Rv0095c~~~MTB00061 | 0.867 | 0.577 | 0.011 | 2.8 | 1.23 | -1.55 | 16.44464677 | 14.04614677 | 18.84314677 | RIF | 1 |
| group_235-group_317-PE27A2 | 0.343 | 0.99 | 0.00159 | 2.6 | 0.975 | 0.0336 | 13.46373804 | 11.56248804 | 15.36498804 | RIF | 1 |
| Rv2336-cysE-cysK1 | 0.924 | 0.0175 | 0.0237 | 2.18 | 1.02 | -1.03 | 8.846306259 | 6.857306259 | 10.83530626 | RIF | 1 |
| group_303-group_217-group_3122 | 0.667 | 0.0216 | 0.0121 | 2.05 | 0.866 | -0.339 | 7.767901106 | 6.079201106 | 9.456601106 | RIF | 1 |
| Rv2083-group_312-group_2172 | 0.667 | 0.0216 | 0.0121 | 2.05 | 0.866 | -0.339 | 7.767901106 | 6.079201106 | 9.456601106 | RIF | 1 |
| Rv2345-esxN~~~esxO~~~esxL-group_3402 | 0.686 | 0.00806 | 0.0272 | 2.02 | 1.02 | -0.407 | 7.538324934 | 5.549324934 | 9.527324934 | RIF | 1 |
| Rv2348c-group_340-esxN~~~esxO~~~esxL2 | 0.686 | 0.00806 | 0.0272 | 2.02 | 1.02 | -0.407 | 7.538324934 | 5.549324934 | 9.527324934 | RIF | 1 |
| PPE35-group_329-group_712 | 0.2 | 0.761 | 0.0393 | 1.94 | 1 | 0.517 | 6.958750971 | 5.008750971 | 8.908750971 | RIF | 1 |
| esxS-group_235-group_3172 | 0.448 | 0.412 | 0.0124 | 1.92 | 0.837 | 0.125 | 6.820958469 | 5.188808469 | 8.453108469 | RIF | 1 |
| Rv1761c-group_229-group_3192 | 0.486 | 0.00785 | 0.0356 | 1.87 | 0.945 | 0.0267 | 6.488296399 | 4.645546399 | 8.331046399 | RIF | 1 |
| vapC18-lppA~~~vapB18~~~lppB-lppA2 | 0.295 | 0.428 | 0.0316 | 1.85 | 0.895 | 0.312 | 6.359819523 | 4.614569523 | 8.105069523 | RIF | 1 |
| Rv2542-lppA-lppA~~~vapB18~~~lppB2 | 0.295 | 0.428 | 0.0316 | 1.85 | 0.895 | 0.312 | 6.359819523 | 4.614569523 | 8.105069523 | RIF | 1 |
| group_255-group_303-group_2171 | 0.838 | 0.00127 | 0.0203 | 1.81 | 0.807 | -0.51 | 6.110447432 | 4.536797432 | 7.684097432 | RIF | 1 |
| PPE46~~~PPE47-PE27A-group_3172 | 0.305 | 0.853 | 0.0181 | 1.73 | 0.791 | 0.419 | 5.640653908 | 4.098203908 | 7.183103908 | RIF | 1 |
| group_107-group_333-Rv13592 | 0.552 | 0.416 | 0.013 | 1.7 | 0.727 | 0.0279 | 5.473947392 | 4.056297392 | 6.891597392 | RIF | 1 |
| esxS-PPE47~~~PPE48-PE292 | 0.629 | 0.000188 | 0.0444 | 1.62 | 0.816 | -0.0112 | 5.053090317 | 3.461890317 | 6.644290317 | RIF | 1 |
| Rv1357c-group_107-group_3332 | 0.562 | 0.58 | 0.0321 | 1.47 | 0.718 | 0.14 | 4.349235141 | 2.949135141 | 5.749335141 | RIF | 1 |
| pks18-group_291-lprF1 | 0.455 | 0.671 | 0.00185 | 5.5 | 2.43 | -2.28 | 244.6919323 | 239.9534323 | 249.4304323 | AMK | 1 |
| Rv1761c-group_229-group_3191 | 0.403 | 0.233 | 0.00715 | 4.87 | 2.03 | -1.57 | 130.3209169 | 126.3624169 | 134.2794169 | AMK | 1 |
| group_245-mamB-Rv2025c1 | 0.805 | 0.00047 | 0.00664 | 3.99 | 1.79 | -2.88 | 54.05488936 | 50.56438936 | 57.54538936 | AMK | 1 |
| mmpL5-msrP-pimC_2~~~pimC_1~~~pimC1 | 0.377 | 0.0107 | 0.0196 | 3.86 | 1.89 | -0.998 | 47.46535137 | 43.77985137 | 51.15085137 | AMK | 1 |
| msrP-pimC_2~~~pimC_1~~~pimC-plcA~~~plcD1 | 0.377 | 0.0107 | 0.0196 | 3.86 | 1.89 | -0.998 | 47.46535137 | 43.77985137 | 51.15085137 | AMK | 1 |
| mmpL5-cut1-wag221 | 0.182 | 0.411 | 0.00972 | 3.61 | 1.59 | -0.216 | 36.96605281 | 33.86555281 | 40.06655281 | AMK | 1 |
| Rv3160c-group_183-PPE531 | 0.688 | 0.00741 | 0.028 | 3.35 | 1.72 | -1.96 | 28.50273364 | 25.14873364 | 31.85673364 | AMK | 1 |
| Rv2820c-Rv2819c-Rv2818c~~~csm6 | 0.519 | 0.0228 | 0.0355 | 3.28 | 1.73 | -1.29 | 26.5757727 | 23.2022727 | 29.9492727 | AMK | 1 |
| esxP-Rv2348c-plcB~~~plcC1 | 0.234 | 0.892 | 0.0346 | 2.8 | 1.46 | -0.242 | 16.44464677 | 13.59764677 | 19.29164677 | AMK | 1 |
| Rv2348c-plcB~~~plcC-plcB1 | 0.883 | 0.0146 | 0.0087 | 2.77 | 1.24 | -2.03 | 15.95863401 | 13.54063401 | 18.37663401 | AMK | 1 |
| group_291-lprF-Rv1367c1 | 0.442 | 0.539 | 0.0443 | 2.58 | 1.39 | -0.777 | 13.19713816 | 10.48663816 | 15.90763816 | AMK | 1 |
| msrP-mmpL5-cut11 | 0.26 | 0.615 | 0.00845 | 2.56 | 1.07 | -0.26 | 12.93581732 | 10.84931732 | 15.02231732 | AMK | 1 |
| group_242-esxL~~~esxN-group_3321 | 0.247 | 0.467 | 0.0308 | 2.34 | 1.22 | -0.152 | 10.38123656 | 8.002236563 | 12.76023656 | AMK | 1 |
| Rv2014-Rv2015c~~~Rv1765c-Rv20161 | 0.545 | 0.149 | 0.0495 | 2.34 | 1.38 | -0.821 | 10.38123656 | 7.690236563 | 13.07223656 | AMK | 1 |
| Rv3679-Rv3680-whiB4 | 0.844 | 0.914 | 0.0297 | 1.9 | 0.906 | -1.11 | 6.685894442 | 4.919194442 | 8.452594442 | AMK | 1 |
| Rv3680-whiB4-ponA2~~~mtgA | 0.844 | 0.914 | 0.0297 | 1.9 | 0.906 | -1.11 | 6.685894442 | 4.919194442 | 8.452594442 | AMK | 1 |
| fadD19-group_190-PE_PGRS56~~~PE_PGRS551 | 0.403 | 0.00214 | 0.0228 | 1.8 | 0.825 | -0.223 | 6.049647464 | 4.440897464 | 7.658397464 | AMK | 1 |
| group_190-PE_PGRS56~~~PE_PGRS55-Rv3510c1 | 0.403 | 0.00214 | 0.0228 | 1.8 | 0.825 | -0.223 | 6.049647464 | 4.440897464 | 7.658397464 | AMK | 1 |
| group_216-pyrH-Rv28841 | 0.818 | 0.00123 | 0.038 | 1.8 | 0.903 | -1.06 | 6.049647464 | 4.288797464 | 7.810497464 | AMK | 1 |
| group_220-group_245-mamB1 | 0.74 | 0.00162 | 0.047 | 1.75 | 0.898 | -0.874 | 5.754602676 | 4.003502676 | 7.505702676 | AMK | 1 |
| Rv2817c-Cas2-group_1197 | 0.338 | 0.78 | 0.0234 | 3.64 | 1.8 | 0.907 | 38.09183673 | 34.58183673 | 41.60183673 | FQ | 1 |
| ctpA-mtn-Rv00907 | 0.323 | 0.865 | 0.0453 | 2.98 | 1.67 | 1.24 | 19.68781664 | 16.43131664 | 22.94431664 | FQ | 1 |
| mtn-Rv0090-Rv00897 | 0.323 | 0.865 | 0.0453 | 2.98 | 1.67 | 1.24 | 19.68781664 | 16.43131664 | 22.94431664 | FQ | 1 |
| Rv2015c~~~Rv1765c-Rv2016-Rv20172 | 0.58 | 0.00314 | 0.00417 | 4.8 | 2.19 | -2.64 | 121.5104175 | 117.2399175 | 125.7809175 | KAN | 1 |
| fadD1-Rv1751-PPE243 | 0.239 | 0.0363 | 0.013 | 2.82 | 1.36 | 0.269 | 16.77685067 | 14.12485067 | 19.42885067 | KAN | 1 |
| Rv1754c-PPE24-Rv17513 | 0.239 | 0.0363 | 0.013 | 2.82 | 1.36 | 0.269 | 16.77685067 | 14.12485067 | 19.42885067 | KAN | 1 |
| Rv1205-fadD6-group_123 | 0.864 | 0.0571 | 0.0185 | 2.72 | 1.23 | -1.46 | 15.18032224 | 12.78182224 | 17.57882224 | KAN | 1 |
| gpgS-folP2-group_123 | 0.864 | 0.0571 | 0.0185 | 2.72 | 1.23 | -1.46 | 15.18032224 | 12.78182224 | 17.57882224 | KAN | 1 |
| Rv2014-Rv2015c~~~Rv1765c-Rv20163 | 0.58 | 0.0473 | 0.0363 | 2.67 | 1.38 | -1.04 | 14.43996919 | 11.74896919 | 17.13096919 | KAN | 1 |
| esxS-PPE47~~~PPE48-PE293 | 0.67 | 0.0000379 | 0.0244 | 2.26 | 1.01 | -0.569 | 9.583089167 | 7.613589167 | 11.55258917 | KAN | 1 |
| ctpA-mtn-Rv00904 | 0.303 | 0.338 | 0.02 | 3.65 | 1.82 | 1.04 | 38.47466605 | 34.92566605 | 42.02366605 | MOX | 1 |
| mtn-Rv0090-Rv00894 | 0.303 | 0.338 | 0.02 | 3.65 | 1.82 | 1.04 | 38.47466605 | 34.92566605 | 42.02366605 | MOX | 1 |
| Rv2817c-Cas2-group_1194 | 0.333 | 0.64 | 0.046 | 3.26 | 1.75 | 0.83 | 26.04953714 | 22.63703714 | 29.46203714 | MOX | 1 |
| group_242-esxL~~~esxN-group_3324 | 0.273 | 0.458 | 0.0441 | 2.79 | 1.52 | 1.22 | 16.2810198 | 13.3170198 | 19.2450198 | MOX | 1 |
| mmpL7-Rv2943-group_664 | 0.697 | 0.0553 | 0.0261 | 2.02 | 0.953 | 0.722 | 7.538324934 | 5.679974934 | 9.396674934 | MOX | 1 |
| lppX-Rv2944-group_664 | 0.697 | 0.0553 | 0.0261 | 2.02 | 0.953 | 0.722 | 7.538324934 | 5.679974934 | 9.396674934 | MOX | 1 |
| Rv2813-group_273-group_1194 | 0.405 | 0.478 | 0.0102 | 2.83 | 1.33 | -0.186 | 16.94546082 | 14.35196082 | 19.53896082 | OFX | 1 |
| Rv2812-Rv2813-group_2734 | 0.417 | 0.3 | 0.0246 | 2.64 | 1.37 | -0.127 | 14.01320361 | 11.34170361 | 16.68470361 | OFX | 1 |
| Rv2345-esxN~~~esxO~~~esxL-group_3405 | 0.774 | 0.0785 | 0.0495 | 2.64 | 1.52 | -1.17 | 14.01320361 | 11.04920361 | 16.97720361 | OFX | 1 |
| Rv2348c-group_340-esxN~~~esxO~~~esxL5 | 0.774 | 0.0785 | 0.0495 | 2.64 | 1.52 | -1.17 | 14.01320361 | 11.04920361 | 16.97720361 | OFX | 1 |
| plcD~~~plcB-Rv1754c-PPE244 | 0.119 | 0.51 | 0.0471 | 2.53 | 1.39 | 0.546 | 12.55350614 | 9.843006137 | 15.26400614 | OFX | 1 |
| group_303-group_217-group_3125 | 0.643 | 0.0202 | 0.0107 | 2.39 | 0.995 | -0.437 | 10.91349394 | 8.973243943 | 12.85374394 | OFX | 1 |
| Rv2083-group_312-group_2175 | 0.643 | 0.0202 | 0.0107 | 2.39 | 0.995 | -0.437 | 10.91349394 | 8.973243943 | 12.85374394 | OFX | 1 |
| Cas2-group_119-group_2734 | 0.333 | 0.867 | 0.0313 | 2.16 | 1.07 | 0.261 | 8.671137658 | 6.584637658 | 10.75763766 | OFX | 1 |
| group_242-esxL~~~esxN-group_3325 | 0.286 | 0.823 | 0.0264 | 1.96 | 0.944 | 0.261 | 7.099327065 | 5.258527065 | 8.940127065 | OFX | 1 |
| group_255-group_303-group_2173 | 0.857 | 0.00385 | 0.0404 | 1.96 | 0.949 | -0.671 | 7.099327065 | 5.248777065 | 8.949877065 | OFX | 1 |
| Rv1356c-Rv1357c-group_1076 | 0.532 | 0.0267 | 0.0000892 | 4.57 | 1.81 | 0.123 | 96.54410977 | 93.01460977 | 100.0736098 | PZA | 1 |
| Rv1357c-group_107-group_3336 | 0.519 | 0.122 | 0.000292 | 4.55 | 1.85 | 0.138 | 94.63240831 | 91.02490831 | 98.23990831 | PZA | 1 |
| group_107-group_333-Rv13596 | 0.506 | 0.153 | 0.000491 | 4.38 | 1.84 | 0.219 | 79.83803341 | 76.25003341 | 83.42603341 | PZA | 1 |
| PPE3-group_1-PE_PGRS46 | 0.405 | 0.621 | 0.00901 | 4.35 | 2.22 | 0.0768 | 77.47846293 | 73.14946293 | 81.80746293 | PZA | 1 |
| Rv3179-Rv3180c-Rv3181c6 | 0.684 | 0.34 | 0.0182 | 4.14 | 2.65 | -0.757 | 62.80282145 | 57.63532145 | 67.97032145 | PZA | 1 |
| Rv3178-Rv3179-Rv3180c6 | 0.684 | 0.34 | 0.0182 | 4.14 | 2.65 | -0.757 | 62.80282145 | 57.63532145 | 67.97032145 | PZA | 1 |
| thrA-thrC-thrB5 | 0.595 | 0.238 | 0.0184 | 4.13 | 2.1 | -0.534 | 62.17792293 | 58.08292293 | 66.27292293 | PZA | 1 |
| thrC-thrB-rho5 | 0.595 | 0.238 | 0.0184 | 4.13 | 2.1 | -0.534 | 62.17792293 | 58.08292293 | 66.27292293 | PZA | 1 |
| metE-Rv1134-PPE164 | 0.722 | 0.439 | 0.0412 | 3.56 | 2.57 | -0.637 | 35.16319715 | 30.15169715 | 40.17469715 | PZA | 1 |
| embR-Rv1268c-Rv1269c3 | 0.722 | 0.439 | 0.0412 | 3.56 | 2.57 | -0.637 | 35.16319715 | 30.15169715 | 40.17469715 | PZA | 1 |
| Rv3294c-pcd-Rv32925 | 0.722 | 0.439 | 0.0412 | 3.56 | 2.57 | -0.637 | 35.16319715 | 30.15169715 | 40.17469715 | PZA | 1 |
| lhr-Rv3295-Rv3294c5 | 0.722 | 0.439 | 0.0412 | 3.56 | 2.57 | -0.637 | 35.16319715 | 30.15169715 | 40.17469715 | PZA | 1 |
| Rv1271c-lprA-Rv1269c3 | 0.722 | 0.439 | 0.0412 | 3.56 | 2.57 | -0.637 | 35.16319715 | 30.15169715 | 40.17469715 | PZA | 1 |
| lppA~~~vapB18~~~lppB-group_345-lppA5 | 0.329 | 0.353 | 0.0232 | 3.37 | 1.76 | 0.824 | 29.07852706 | 25.64652706 | 32.51052706 | PZA | 1 |
| vapC18-lppA~~~vapB18~~~lppB-group_3455 | 0.722 | 0.166 | 0.0203 | 3.27 | 1.63 | -0.474 | 26.31133934 | 23.13283934 | 29.48983934 | PZA | 1 |
| Rv2014-Rv2015c~~~Rv1765c-Rv20166 | 0.595 | 0.534 | 0.0387 | 2.74 | 1.49 | 0.365 | 15.4869851 | 12.5814851 | 18.3924851 | PZA | 1 |
| PE_PGRS4~~~PE_PGRS3-PE_PGRS4-group_16 | 0.342 | 0.296 | 0.0125 | 2.41 | 1.08 | 1.1 | 11.13396115 | 9.027961145 | 13.23996115 | PZA | 1 |
| enc-Rv0797-Rv0794c6 | 0.468 | 0.0297 | 0.0371 | 2.19 | 1.2 | 0.796 | 8.935213115 | 6.595213115 | 11.27521311 | PZA | 1 |
| Rv0797-Rv0794c-Rv07936 | 0.468 | 0.0297 | 0.0371 | 2.19 | 1.2 | 0.796 | 8.935213115 | 6.595213115 | 11.27521311 | PZA | 1 |
| smc-ftsY-group_2676 | 0.468 | 0.104 | 0.00616 | 2.07 | 0.795 | 0.893 | 7.924823118 | 6.374573118 | 9.475073118 | PZA | 1 |
| glnB-amt-group_2676 | 0.468 | 0.104 | 0.00616 | 2.07 | 0.795 | 0.893 | 7.924823118 | 6.374573118 | 9.475073118 | PZA | 1 |
| Rv2345-esxN~~~esxO~~~esxL-group_3401 | 0.739 | 0.908 | 0.0124 | 4.3 | 2.25 | -4.15 | 73.6997937 | 69.3122937 | 78.0872937 | CAP | 1 |
| Rv2348c-group_340-esxN~~~esxO~~~esxL1 | 0.739 | 0.908 | 0.0124 | 4.3 | 2.25 | -4.15 | 73.6997937 | 69.3122937 | 78.0872937 | CAP | 1 |
| rpsJ-group_343-Rv0698 | 0.33 | 0.019 | 0.00588 | 4.04 | 1.87 | -2.38 | 56.82634281 | 53.17984281 | 60.47284281 | CAP | 1 |
| Rv0696-Rv0697-Rv0698 | 0.33 | 0.019 | 0.00588 | 4.04 | 1.87 | -2.38 | 56.82634281 | 53.17984281 | 60.47284281 | CAP | 1 |
| fadE4-Rv0232-gabD1 | 0.17 | 0.852 | 0.0163 | 3.52 | 1.8 | -1.37 | 33.78442846 | 30.27442846 | 37.29442846 | CAP | 1 |
| Rv0235c-gabD1-Rv0232 | 0.17 | 0.852 | 0.0163 | 3.52 | 1.8 | -1.37 | 33.78442846 | 30.27442846 | 37.29442846 | CAP | 1 |
| Rv3679-Rv3680-whiB41 | 0.864 | 0.247 | 0.00077 | 2.93 | 0.969 | -3.62 | 18.7276305 | 16.8380805 | 20.6171805 | CAP | 1 |
| Rv3680-whiB4-ponA2~~~mtgA1 | 0.864 | 0.247 | 0.00077 | 2.93 | 0.969 | -3.62 | 18.7276305 | 16.8380805 | 20.6171805 | CAP | 1 |
| group_118-MTB0005-PE22 | 0.205 | 0.00104 | 0.0288 | 1.76 | 0.845 | -0.79 | 5.812437394 | 4.164687394 | 7.460187394 | CAP | 1 |
| group_284-Rv3346c-PPE54 | 0.261 | 0.393 | 0.0453 | 1.61 | 0.884 | -1.01 | 5.002811228 | 3.279011228 | 6.726611228 | CAP | 1 |
| Rv3342-PPE54-Rv3346c | 0.261 | 0.393 | 0.0453 | 1.61 | 0.884 | -1.01 | 5.002811228 | 3.279011228 | 6.726611228 | CAP | 1 |
| Rv0890c-group_408-Rv0892 | 0.398 | 0.0102 | 0.00858 | 1.49 | 0.589 | -1.24 | 4.437095519 | 3.288545519 | 5.585645519 | CAP | 1 |
| citA-Rv0890c-group_408 | 0.398 | 0.0102 | 0.00858 | 1.49 | 0.589 | -1.24 | 4.437095519 | 3.288545519 | 5.585645519 | CAP | 1 |

# Table S6. Panaroo output structural variants only found in lineage 1 isolates.

| **Lineage 1 specific Panaroo structural variants** |
| --- |
| Rv2337c-moeW-group_185 |
| group_185-mmpL9-PE_PGRS39 |
| mmpL9-group_185-moeW |
| group_225-Rv3901c-group_376 |
| group_113-Rv2015c-group_319 |
| Rv1268c-embR-group_268 |
| Rv0333-rmlA-MTB0002 |
| group_376-group_176-group_209 |
| group_322-Rv1526c-Rv1524 |
| Rv3899c-group_209-group_176 |
| Rv1526c-Rv1524-Rv1523 |
| Rv2704-Rv2705c-Rv2707 |
| Rv2708c-Rv2707-Rv2705c |
| Rv2015c-group_113-group_293 |
| group_176-group_376-Rv3901c |
| group_229-group_319-Rv2015c |
| embR-group_268-btuD_1~~~Rv1747 |
| aspC-MTB0002-rmlA |

# Table S7. Panaroo output structural variants only found in lineage 2 isolates.

| **group_109-group_207-group_374** |
| --- |
| Rv2285-Rv2286c-group_289 |
| group_238-group_296-group_332 |
| MTB0005-group_116-PPE34 |
| group_115-group_337-group_331 |
| group_48-Rv3427c~~~Rv3428c-group_301 |
| group_122-group_134-esxS |
| Rv2016-group_117-group_320 |
| Rv2666-Rv2665-group_119 |
| mpt64-Rv1979c-group_397 |
| group_294-group_120-group_317 |
| Rv2077c-group_110-group_290 |
| group_202-whiB4-ponA2~~~mtgA |
| group_120-group_294-esxS |
| Rv0793-Rv0794c-group_252 |
| group_120-group_294-PPE39 |
| uspB-uspC-group_353 |
| PPE40-PPE39-group_130 |
| dxs~~~dxs2-lytB1-group_262 |
| group_135-Rv3179-group_282 |
| group_60-idsB-group_137 |
| Rv2079-Rv2078-group_290 |
| dxs~~~dxs2-group_126-group_299 |
| aceAb~~~icl2~~~aceAa-group_71-PPE35 |
| group_320-group_262-group_337 |
| group_110-group_290-lprP |
| group_134-group_122-group_317 |
| group_380-group_110-group_290 |
| group_397-Rv1977-group_326 |
| MTB0005-group_130-group_116 |
| group_126-lytB1-dxs~~~dxs2 |
| gnd1-group_114-group_295 |
| group_115-group_337-group_262 |
| cyp139-Rv1668c~~~Rv1667c~~~btuD_2~~~btuD_1-group_113 |
| group_238-esxL~~~esxN-group_242 |
| group_116-group_320-Rv2018 |
| PPE46~~~PPE47-PE27A-group_289 |
| group_207-group_114-group_235 |
| group_411-Rv0021c-whiB5 |
| Rv0059-dnaB-rplI |
| Rv2663-Rv2664-group_273 |
| Rv2015c~~~Rv1765c-Rv2016-group_295 |
| group_126-group_299-lytB1 |
| group_114-group_235-group_317 |
| Rv1928c-group_320-hcaB~~~Rv1928c |
| group_134-group_122-Rv0921 |
| enc-Rv0797-group_135 |
| group_137-group_262-lytB1 |
| group_262-group_320-group_331 |
| PPE29-group_117-group_295 |
| group_324-glnA3-mdtD_1~~~mdtL~~~Rv1877 |
| sirA-Rv2390c-group_290 |
| group_117-PPE29-PPE30 |
| group_222-moaC3-group_311 |
| Rv2015c~~~Rv1765c-group_318-group_295 |
| group_252-group_207-Rv0842 |
| group_114-group_317-group_235 |
| group_130-group_118-Rv2023A |
| group_257-group_326-Rv1977 |
| group_121-group_235-group_317 |
| group_203-group_136-group_311 |
| group_293-group_113-Rv1668c~~~Rv1667c~~~btuD_2~~~btuD_1 |
| Rv1670-Rv1671-Rv1674c |
| group_239-Rv3180c-Rv3181c |
| Rv1134-group_374-group_290 |
| Rv2181-Rv2180c-group_238 |
| Rv3179-group_207-group_252 |
| group_385-Rv1815-Rv1816 |
| moaC3-group_311-group_298 |
| group_60-idsB-group_299 |
| PPE16-group_110-group_290 |
| PPE46~~~PPE47-PE27A-group_282 |
| group_294-group_120-group_332 |
| group_187-Rv3427c-group_301 |
| fprB-group_93-SpmT |
| Rv1134-group_374-group_207 |
| group_395-group_110-group_290 |
| group_112-group_292-group_42 |
| Rv2285-Rv2286c-group_296 |
| group_121-group_130-group_374 |
| group_110-group_290-Rv2078 |
| group_321-PPE39-glyS |
| group_116-Rv2017-Rv2016 |
| group_294-group_120-Rv3007c~~~rutF |
| group_110-PPE34-PPE35 |
| Rv1498c-Rv1498A-arnC |
| group_118-MTB0005-group_245 |
| ctpD-group_112-group_292 |
| PPE47~~~PPE48-esxS-group_290 |
| group_118-MTB0005-PE22 |
| Rv2230c-cobC-group_238 |
| group_207-group_252-group_407 |
| group_122-group_317-group_235 |
| idsB-lytB1-group_299 |
| group_289-group_121-yjcE |
| gatB-Rv3008-group_294 |
| MTB0005-PE22-p27 |
| group_130-group_121-group_317 |
| group_218-Rv1674c-Rv1671 |
| group_296-group_238-esxL~~~esxN |
| group_239-group_135-Rv3179 |
| Rv2541-Rv2542-group_345 |
| group_183-PPE53-ndhB~~~nuoM |
| embR-Rv1268c-group_360 |
| group_126-group_299-fadD25~~~dltA_1 |
| group_282-Rv3179-group_298 |
| group_121-group_289-Rv2286c |
| group_122-pip-Rv0839 |
| group_305-group_263-PE_PGRS4 |
| group_122-group_134-Rv0920c |
| group_121-group_130-MTB0005 |
| rcsC~~~kdpE-group_103-kdpF |
| group_115-PPE34-PPE35 |
| group_116-Rv1930c-Rv1931c |
| ptpA-ptkA-group_296 |
| group_110-group_290-esxS |
| Rv2807-group_207-group_252 |
| group_121-group_317-group_235 |
| vapC28-group_298-group_203 |
| Rv3180c-group_239-group_135 |
| group_122-group_207-Rv2807 |
| PPE34-group_115-group_337 |
| group_120-group_294-PE27A |
| Rv1928c-Rv1930c-Rv1931c |
| Rv2023c-Rv2023A-group_118 |
| pknF-idi-pknE |
| group_134-Rv2807-group_250 |
| group_114-group_295-erg3 |
| group_135-group_252-Rv0794c |
| sdaA-glyA2-Rv0074~~~hutI |
| group_121-group_130-group_256 |
| group_130-group_121-PE22 |
| Rv1501-Rv1500-group_262 |
| MTB0005-group_118-group_234 |
| group_112-group_292-PE_PGRS29 |
| MTB0005-group_331-group_320 |
| PPE34-group_116-group_320 |
| PE_PGRS4~~~PE_PGRS3-PE_PGRS4-group_263 |
| group_117-group_295-Rv2016 |
| group_290-group_110-Rv2016 |
| lprF-Rv1371-group_111 |
| group_252-group_407-Rv3129 |
| group_120-group_294-Rv2820c |
| Rv1813c-erg3-group_295 |
| group_110-group_290-group_374 |
| Rv3178-group_252-group_282 |
| group_116-PE22-p27 |
| group_125-group_298-Rv3179 |
| group_179-ppx2-Rv1024 |
| group_122-Rv0921-Rv0922 |
| group_120-group_317-group_235 |
| group_119-group_273-Rv2664 |
| Rv1134-group_374-group_130 |
| group_109-group_235-group_317 |
| Rv2019-group_117-group_295 |
| group_235-group_116-group_320 |
| group_130-group_118-group_220 |
| group_291-group_111-Rv1371 |
| group_117-group_320-group_318 |
| group_137-group_262-Rv1500 |
| sigJ-Rv3327~~~Rv0797-group_108 |
| glyA2-Rv0074~~~hutI-Rv0075~~~dapL |
| Rv1135A-PPE16-group_110 |
| group_131-group_125-group_298 |
| group_122-group_134-Rv2807 |
| group_117-group_130-group_318 |
| rraA-ethA-Rv3857c |
| group_125-group_135-Rv3179 |
| Rv1927-hcaB~~~Rv1928c-group_320 |
| Rv2434c-group_256-group_294 |
| cobC-group_238-group_296 |
| group_108-group_136-group_203 |
| esxS-group_134-group_121 |
| group_250-Rv2807-group_282 |
| Rv2813-group_120-group_294 |
| Rv2017-Rv2016-group_117 |
| Rv2665-group_119-group_273 |
| rpsR1-rplI-dnaB |
| Rv1271c-lprA-group_360 |
| PPE40-PPE39-group_320 |
| group_252-group_282-Rv3179 |
| group_121-yjcE-cdh |
| Rv0755A-Rv0756c-group_289 |
| group_116-group_320-PPE39 |
| Rv1930c-group_116-group_320 |
| PPE34-group_110-group_290 |
| group_140-group_136-group_203 |
| group_42-fadD25~~~dltA_1-group_299 |
| Rv2812-Rv2813-group_120 |
| group_207-group_109-group_235 |
| Rv0843-Rv0842-group_134 |
| group_207-esxS-PPE47~~~PPE48 |
| group_121-group_130-PE27A |
| group_395-rpfD-hemN |
| Rv0843-Rv0842-group_207 |
| vapC17-Rv2525c-fas |
| group_407-group_122-group_134 |
| Rv0610c-group_46-group_311 |
| PPE47~~~PPE48-esxS-group_294 |
| Rv1520-group_42-group_292 |
| Rv2812-Rv2813-group_119 |
| Rv0919-Rv0920c-group_134 |
| group_122-group_134-Rv0842 |
| PPE46~~~PPE47-PE27A-group_122 |
| group_130-group_256-Rv2434c |
| Rv3177-Rv3178-group_252 |
| group_134-group_252-group_207 |
| group_110-group_290-Rv2390c |
| group_235-group_317-group_282 |
| arnB~~~Rv3402c-group_309-group_401 |
| group_120-group_294-Rv3008 |
| group_122-group_407-Rv3129 |
| PPE46~~~PPE47-PE27A-group_130 |
| group_130-group_117-Rv2016 |
| Rv3427c-group_301-Rv3427c~~~Rv3428c |
| group_134-group_122-pip |
| group_194-Rv3857c-ethA |
| group_116-group_320-hcaB~~~Rv1928c |
| group_75-plcA-plcB~~~plcC |
| group_121-group_332-group_75 |
| group_252-group_282-Rv2807 |
| Rv3327~~~Rv0797-group_311-group_136 |
| group_134-group_121-group_235 |
| group_116-group_320-esxS |
| aroK-aroF-Rv2542 |
| Rv3327~~~Rv0797-group_311-group_300 |
| group_118-group_220-Rv2023A |
| group_130-group_245-mamB |
| group_122-group_235-group_317 |
| idsB-group_137-group_262 |
| group_134-group_122-PE27A |
| group_130-group_121-PPE16 |
| Rv2015c~~~Rv1765c-group_318-group_320 |
| group_118-group_130-group_245 |
| phoP-group_109-group_289 |
| ilvX-PE_PGRS57-PE_PGRS54 |
| guaB1-group_195-group_295 |
| Rv2019-Rv2018-group_320 |
| aroG-Rv2179c-group_296 |
| Rv2821c-Rv2820c-group_273 |
| esxS-group_207-group_114 |
| group_116-MTB0005-group_331 |
| group_118-MTB0005-group_130 |
| PE_PGRS57-PE_PGRS54-PE_PGRS53 |
| Rv2813-group_119-group_273 |
| group_295-group_117-Rv2016 |
| group_117-group_295-group_318 |
| group_136-group_203-group_300 |
| eno-Rv1024-ppx2 |
| trxA-ctpD-group_112 |
| vapB37-group_234-group_118 |
| fadD25~~~dltA_1-group_112-group_292 |
| group_120-Rv3007c~~~rutF-lppZ |
| group_103-rcsC~~~kdpE-group_179 |
| group_117-Rv2019-Rv2020c |
| group_110-Rv2077c-Rv2076c |
| Rv1930c-Rv1928c-group_320 |
| group_407-group_252-group_282 |
| group_110-group_290-group_318 |
| group_262-group_137-Rv1359 |
| group_116-group_320-group_332 |
| group_134-group_122-group_235 |
| Rv2016-group_116-group_320 |
| group_296-group_238-yjcE |
| rbsK-group_120-group_294 |
| cyp140-group_324-glnA3 |
| group_116-PPE34-PPE35 |
| group_113-group_293-Rv1670 |
| group_207-Rv3179-group_282 |
| arnC-group_137-group_262 |
| vapB28-vapC28-group_298 |
| Rv2015c~~~Rv1765c-group_318-group_290 |
| aroF-Rv2542-group_345 |
| Rv2180c-group_238-group_296 |
| Rv3180c-group_125-group_135 |
| Rv3327~~~Rv0797-group_311-group_46 |
| PE27A-group_282-group_317 |
| PPE46~~~PPE47-PE27A-group_120 |
| group_116-group_320-group_318 |
| Rv2017-group_116-group_320 |
| group_320-group_331-group_337 |
| group_126-group_299-idsB |
| group_289-group_114-group_317 |
| group_122-group_134-PE27A |
| Rv1919c-PPE35-group_338 |
| group_130-group_121-group_332 |
| rpfD-group_395-group_110 |
| PPE40-PPE39-group_116 |
| group_114-group_289-PE27A |
| group_121-PE22-p27 |
| group_131-group_135-group_239 |
| group_93-fprB-Rv0885 |
| group_112-fadD25~~~dltA_1-mmpL12 |
| phoR-phoP-group_109 |
| lytB1-group_239-idsB |
| PPE46~~~PPE47-PE27A-group_134 |
| group_136-group_108-Rv3327~~~Rv0797 |
| group_238-group_296-ptkA |
| enc-Rv0797-group_207 |
| Rv2015c~~~Rv1765c-group_318-group_130 |
| PPE47~~~PPE48-esxS-group_320 |
| group_75-group_332-group_296 |
| group_60-idsB-group_239 |
| Rv2348c-plcB~~~plcC-plcA |
| group_130-group_116-PE22 |
| Rv2017-Rv2016-group_116 |
| Rv3378c-dxs~~~dxs2-group_126 |
| group_385-group_114-group_295 |
| fipA-fhaA-group_411 |
| Rv3679-Rv3680-group_202 |
| group_413-Rv2319c-rocE |
| blaI-blaR-gnd1 |
| group_116-group_235-group_317 |
| Rv0961-lprP-group_290 |
| group_121-group_130-PPE39 |
| group_119-group_273-Rv2820c |
| group_134-esxS-PPE47~~~PPE48 |
| group_238-yjcE-cdh |
| group_296-PPE39-PPE40 |
| tgs1-Rv3129-group_407 |
| metE-Rv1134-group_374 |
| group_407-group_122-group_207 |
| group_140-group_298-group_311 |
| Rv2286c-group_296-group_238 |
| group_380-group_316-group_325 |
| moaA1~~~moaA-group_300-group_311 |
| Rv1671-Rv1670-group_293 |
| group_120-rbsK-Rv2437 |
| group_75-group_332-group_120 |
| group_207-group_252-Rv3190c |
| group_71-group_338-PPE35 |
| Rv2821c-Rv2820c-group_294 |
| group_294-group_120-PE27A |
| group_1-PE_PGRS4~~~PE_PGRS3-group_263 |
| nuoL-ndhB~~~nuoM-PPE53 |
| group_114-group_295-group_195 |
| PPE46~~~PPE47-PE27A-group_294 |
| Rv2179c-group_296-group_238 |
| group_239-lytB1-dxs~~~dxs2 |
| Rv3178-Rv3179-group_135 |
| Rv1742-pknE-idi |
| fadE15-PE_PGRS29-group_292 |
| Rv0797-group_135-group_252 |
| Rv1919c-PPE35-group_71 |
| lppA~~~vapB18~~~lppB-group_345-Rv2542 |
| group_324-cyp140-lppE |
| group_122-group_134-group_252 |
| lytB1-group_126-group_299 |
| Rv1135A-PPE16-group_121 |
| group_120-group_294-group_256 |
| Rv1523-mmpL12-group_126 |
| Rv3400-Rv3401-group_401 |
| Rv2525c-vapC17-mrr |
|  |

# Table S8. Panaroo output structural variants only found in lineage 3 isolates.

| **Lineage 3 specific Panaroo structural variants** |
| --- |
| fadD19-caiD_1~~~echA19-cyp142 |
| group_263-PE_PGRS4~~~PE_PGRS3-PE_PGRS4 |
| Rv3519-cyp142-caiD_1~~~echA19 |
| Rv3845-group_172-group_126 |
| group_250-group_207-group_252 |
| Rv1722-group_193-group_113 |
| group_172-MTB0010-group_287 |
| group_120-group_294-Rv2813 |
| group_251-group_119-group_273 |
| group_280-group_108-Rv0395 |
| group_120-group_294-Rv2809 |
| Rv1502-Rv1503c~~~Rv1504c~~~wecE-group_292 |
| Rv3845-group_172-MTB0010 |
| PE29-PPE47~~~PPE48-group_130 |
| group_294-group_120-Rv2808 |
| group_6-Rv1505c-Rv1506c |
| group_108-group_280-Rv0396 |
| hpt-lpqG-group_427 |
| Rv2286c-group_238-group_296 |
| group_121-group_130-PPE47~~~PPE48 |
| group_33-dprE1-dprE2 |
| Rv0394c-Rv0395-group_108 |
| Rv2812-Rv2813-group_294 |
| Rv0799c-enc-group_122 |
| group_6-group_112-group_292 |
| Rv3736-Rv3737-Rv3740c |
| group_296-yjcE-cdh |
| ilvX-PE_PGRS57-PE_PGRS53 |
| group_114-group_290-Rv1804c |
| group_252-Rv2807-Rv3129 |
| group_136-group_108-group_280 |
| mpt64-Rv1979c-group_326 |
| recA-recX-Rv2735c |
| group_238-Rv2286c-Rv2285 |
| group_289-group_109-PPE12 |
| group_246-lppT-group_290 |
| Rv0394c-Rv0395-group_136 |
| Rv3741c-Rv3740c-Rv3737 |
| group_172-group_126-group_299 |
| group_238-group_296-yjcE |
| vapB37-group_234-MTB0005 |
| PPE34-group_337-group_338 |
| sodA-group_221-group_299 |
| Rv2811-Rv2809-group_294 |
| group_207-group_252-Rv2807 |
| Rv2821c-Rv2820c-group_370 |
| Rv3770A-Rv3771c-hisC2 |
| Rv3770c-Rv3770A-Rv3771c |
| Rv3788-Rv3789-group_33 |
| group_118-MTB0005-group_234 |
| Rv2067c-cobI~~~cysG-cobG |
| Rv2734-group_251-group_119 |
| PE29-PPE47~~~PPE48-group_207 |
| Rv0756c-Rv0755A-group_289 |
| group_119-group_273-Rv2735c |
| group_340-esxO-Rv2345 |
| MTB0010-group_287-group_221 |
| Rv3074-Rv3073c-group_358 |
| group_112-group_292-Rv1503c~~~Rv1504c~~~wecE |
| group_108-group_136-Rv0395 |
| group_427-PPE65-esxW~~~esxJ |
| Rv2348c-group_340-esxO |
| group_109-group_289-Rv0755A |
| Rv3127-group_250-group_207 |
| group_126-group_299-group_221 |
| enc-group_122-group_134 |
| sodA-group_221-group_111 |
| group_120-Rv2808-group_285 |
| group_257-group_326-Rv1979c |
| group_193-group_113-group_293 |
| group_130-group_121-PPE46~~~PPE47 |
| sodA-group_221-group_287 |
| Rv3069-Rv3070-group_358 |
| PE_PGRS11-PPE12-group_109 |
| cobI~~~cysG-cobG-mazE7 |

# Table S9. Panaroo output structural variants only found in lineage 4 isolates, not including H37Rv.

| **Lineage 4 (no H37Rv) specific Panaroo structural variants** |
| --- |
| virS-Rv3085-adhD |
| group_108-mmpS1-fadD30 |
| Rv0837c-Rv0836c-group_109 |
| group_113-Rv1670-Rv1671 |
| group_203-group_126-group_299 |
| Rv1778c-cyp144-group_114 |
| group_120-group_119-group_273 |
| group_137-group_262-group_265 |
| Rv0007-cwsA-ppiA |
| mazE6-ctpG-ctpF |
| lpqA-esxQ-PPE47~~~PPE48 |
| cyp143-Rv1786-PPE27 |
| group_294-PPE39-group_296 |
| Rv1680-moeX-group_113 |
| group_416-moaD1-moaC1 |
| tgs1-Rv3129-group_207 |
| Cas2-group_119-group_297 |
| Rv2017-Rv2018-group_113 |
| group_379-group_116-group_320 |
| Rv2817c-Cas2-group_294 |
| esxQ-PPE46~~~PPE47-group_294 |
| Rv3122-group_207-cyp141 |
| vapB37-group_234-group_295 |
| group_118-Rv2146c-SepF~~~sepF |
| nuoN-PPE53-Rv3160c |
| Rv2083-group_312-group_255 |
| group_222-group_298-group_311 |
| group_113-group_293-Rv2019 |
| PPE35-group_329-group_115 |
| sigJ-Rv3327~~~Rv0797-group_299 |
| group_273-group_119-group_294 |
| group_320-Rv1754c-PPE24 |
| pimC_2~~~pimC_1~~~pimC-plcA~~~plcD-group_295 |
| group_416-group_109-group_320 |
| group_110-group_294-Rv2282c |
| group_134-group_122-Rv0807 |
| papA4-pks5-group_322 |
| PPE46-group_294-PPE46~~~PPE47 |
| tcrX-Rv3766-Rv3770c |
| group_290-PPE39-group_321 |
| pitB-Rv2280-lppN |
| group_207-Rv3180c-Rv3181c |
| group_187-Rv3427c-Rv3427c~~~Rv3428c |
| PPE40-PPE39-group_330 |
| MTB0005-group_118-Rv2077c |
| Rv1767-Rv1766-group_290 |
| Rv2083-group_303-group_255 |
| eccE5-eccA5-group_114 |
| group_117-Rv1765c~~~Rv2015c-group_319 |
| otsB2-echA18~~~echA181~~~menB-group_205 |
| cysE-group_238-group_296 |
| mmpL9-group_323-moeW |
| PE_PGRS32-Rv1804c-group_289 |
| group_113-cut1-wag22 |
| Rv1975-Rv1976c-Rv1977 |
| group_107-Rv1357c-Rv1353c |
| group_1-group_305-vapC25~~~vapC |
| Rv2283-group_110-group_294 |
| group_236-group_103-kdpF |
| Rv1573-Rv3468c-mhpE |
| PPE46~~~PPE47-PPE46-PE27A |
| group_126-group_203-group_300 |
| rmlB-rmlC-MTB0006 |
| mmpL1-mmpS1-group_339 |
| Rv2023c-mamB-Rv2025c |
| Rv1794-esxO-esxJ~~~esxW |
| Rv1670-group_113-group_293 |
| MTB0005-Rv2078-Rv2079 |
| Rv0048c-mtgA~~~Rv0049-Rv0051 |
| group_292-Rv2775-Rv2776c |
| fic-Rv3640c-group_126 |
| group_332-esxL~~~esxN-group_321 |
| ephE-Rv3672c-Rv3673c |
| adhC-Rv3046c-nrdF2 |
| PE19-esxJ~~~esxW-esxO |
| group_132-group_114-group_289 |
| group_290-group_238-esxL~~~esxN |
| group_112-group_127-dapB |
| Rv3723-cut5a~~~cut5b-group_313 |
| PPE38-group_75-plcA |
| Rv2958c-group_314-Rv2957 |
| group_238-group_296-Rv2282c |
| PPE53-Rv3160c-Rv3161c |
| fadD30-group_339-mmpS1 |
| Rv2084-Rv2083-group_303 |
| Rv2348c-esxP-esxO |
| group_117-group_295-group_17 |
| Rv0797-group_109-group_289 |
| Rv2337c-Rv2336-group_320 |
| pitB-Rv2282c-group_294 |
| rcsC~~~kdpE-kdpD-group_236 |
| Rv3114-Rv3113-group_320 |
| Rv1319c~~~Rv1318c-group_137-group_262 |
| pimC_2~~~pimC_1~~~pimC-plcA~~~plcD-group_293 |
| group_116-Rv2015c~~~Rv1765c-Rv2014 |
| group_118-MTB0005-Rv2078 |
| group_235-esxS-PPE47 |
| Rv2491-Rv2492-group_296 |
| group_292-Rv1505c-Rv1503c~~~Rv1504c~~~wecE |
| bkdC-vapC38-group_296 |
| group_117-group_295-plcA~~~plcD |
| vapC25~~~vapC-PE_PGRS4~~~PE_PGRS3-PE_PGRS4 |
| group_238-group_290-group_332 |
| Rv3637-Rv3638-group_299 |
| glyS-PPE40-group_296 |
| Rv0281-PPE3-PE_PGRS4 |
| group_174-group_117-group_295 |
| Rv3786c-Rv3785~~~fcl-rfbD |
| Rv3324A~~~moaB1-moaA1~~~moaA-group_298 |
| Rv3770A-Rv3770c-Rv3766 |
| pks18-group_291-Rv1371 |
| group_278-MTB0007-glpE~~~MTB0004 |
| group_295-Rv1765c~~~Rv2015c-Rv1766 |
| group_127-group_112-group_292 |
| group_109-group_320-Rv3113 |
| Rv0380c~~~trmH-secE2-Rv0375c |
| cyp139-Rv1668c~~~Rv1667c~~~btuD_2~~~btuD_1-group_293 |
| group_113-group_293-group_61 |
| group_122-group_134-group_390 |
| group_112-group_292-Rv1505c |
| group_112-group_292-Rv2775 |
| group_429-Rv3122-group_207 |
| Rv2308-group_238-group_296 |
| group_134-group_390-lpqQ |
| PE29-PPE47~~~PPE48-esxQ |
| gca-gmhA~~~hisB~~~gmhB-hddA |
| Rv2811-Rv2809-group_119 |
| group_296-group_321-PPE39 |
| Rv1761c-group_229-group_117 |
| pitB-Rv2282c-group_296 |
| group_229-group_117-group_295 |
| group_416-group_122-group_134 |
| group_295-group_117-Rv1765c~~~Rv2015c |
| msrP-mmpL5-group_113 |
| mmpL5-group_113-group_293 |
| group_116-group_320-Rv1754c |
| group_119-group_273-Rv2808 |
| group_113-Rv1754c-PPE24 |
| Rv3468c-Rv1573-Rv1575 |
| sigJ-group_311-group_298 |
| group_130-group_109-Rv0836c |
| Rv1930c-hcaB~~~Rv1928c-mpt63 |
| group_118-MTB0005-trpD |
| group_113-group_293-Rv1668c~~~Rv1667c~~~btuD_2~~~btuD_1 |
| Rv3706c-Rv3707c-asd |
| cut1-group_113-group_293 |
| vapC37-helZ-Rv2100 |
| Rv2956-Rv2957-group_314 |
| PPE40-group_296-PPE39 |
| group_115-group_337-group_329 |
| group_120-group_294-group_332 |
| moaA1~~~moaA-group_298-group_311 |
| group_245-group_117-group_295 |
| Rv0111-gca-gmhA~~~hisB~~~gmhB |
| Rv2074-Rv2075c-Rv2077c |
| pepA-PE_PGRS2-group_346 |
| lppB~~~lppA-group_345-lppA |
| Rv1893-nmo1-group_382 |
| Rv2337c-Rv2336-group_296 |
| MTB0005-group_118-Rv2025c |
| group_207-group_252-group_183 |
| mmpL5-group_61-group_293 |
| group_299-group_126-Rv3640c |
| group_109-group_130-Rv3113 |
| Rv2018-group_113-group_293 |
| Rv1683-Rv1682-group_293 |
| group_300-moaA1~~~moaA-group_311 |
| Rv1352-Rv1353c-Rv1357c |
| group_136-group_108-mmpS1 |
| group_122-Rv0807-purF |
| fgd1-Rv0406c-pks6 |
| group_245-mamB-MTB0005 |
| fadD31-mpt63-hcaB~~~Rv1928c |
| group_288-mmpS1-fadD30 |
| gap-Rv1435c-Rv1434 |
| group_116-group_379-wag22 |
| group_190-group_334-group_361 |
| wag22-cut1-group_293 |
| group_122-group_416-moaD1 |
| group_110-group_290-Rv1766 |
| Rv1318c-group_265-group_262 |
| PPE35-group_329-group_337 |
| group_293-group_113-Rv1754c |
| moaA1~~~moaA-group_311-group_298 |
| vapC38-group_296-Rv2492 |
| PPE35-group_329-aceAb~~~icl2~~~aceAa |
| fprB-Rv0887c-SpmT |
| group_113-group_293-plcA~~~plcD |
| PE19-PPE27-Rv1786 |
| group_329-group_115-group_337 |
| PPE3-group_1-group_305 |
| MTB0007-glpE~~~MTB0004-Rv3114 |
| group_416-group_109-group_289 |
| msrP-mmpL5-group_61 |
| lipM-Rv2283-group_110 |
| murC-ftsZ-yfiH |
| group_130-Rv3113-Rv3114 |
| Rv2959c-Rv2958c-group_314 |
| group_332-group_320-esxL~~~esxN |
| Rv3049c-nrdF2-Rv3046c |
| group_289-Rv3113-Rv3114 |
| group_109-group_289-Rv0794c |
| folD-Rv3355c-PE_PGRS50~~~PE_PGRS49 |
| Rv2020c-Rv2019-group_293 |
| Rv3705c-Rv3706c-Rv3707c |
| PPE53-group_207-group_252 |
| Rv2337c-moeW-group_323 |
| Rv3733c-lig~~~ligC-polD1 |
| group_75-group_332-group_290 |
| group_136-group_108-group_288 |
| Rv3371-otsB2-echA18~~~echA181~~~menB |
| Rv1914c-aceAb~~~icl2~~~aceAa-group_329 |
| PPE35-group_329-group_71 |
| group_294-PPE46-PE27A |
| lprF-Rv1371-group_291 |
| mmsA-fadE9-Rv0740~~~Rv0750 |
| Rv3355c-PE_PGRS50~~~PE_PGRS49-PPE54 |
| enc-Rv0797-group_109 |
| group_17-group_220-Rv2023A |
| group_238-esxL~~~esxN-group_321 |
| PPE40-group_294-PPE39 |
| Rv3327~~~Rv0797-group_299-group_311 |
| Rv2083-group_312-group_240 |
| Rv2022c-Rv2023c-mamB |
| vapB37-vapC37-helZ |
| group_48-Rv3427c~~~Rv3428c-Rv3427c |
| group_108-group_136-mmpL1 |
| group_109-group_289-Rv3113 |
| Rv0401-mmpL1-group_108 |
| group_238-group_296-plcB |
| Rv3669-ephE-Rv3672c |
| lppJ-group_255-group_312 |
| Rv0749A-Rv0740~~~Rv0750-fadE9 |
| murG-murC-ftsZ |
| group_76-group_112-group_292 |
| Rv3322c-group_222-group_298 |
| Rv1508c-Rv1507c-group_76 |
| group_118-MTB0005-group_174 |
| Rv2336-group_296-group_238 |
| group_234-PE22-group_315 |
| group_122-group_134-cpsY |
| PE_PGRS4~~~PE_PGRS3-PE_PGRS4-PPE3 |
| alr-group_286-PPE58 |
| Rv1433-Rv1434-Rv1435c |
| nuoN-PPE53-group_207 |
| MTB0005-trpD-Rv2191 |
| Rv0837c-Rv0836c-group_122 |
| lipM-Rv2283-group_238 |
| Rv3081-virS-Rv3085 |
| PPE39-group_321-esxL~~~esxN |
| xthA-Rv0426c-kdpB~~~Rv0424c |
| group_119-group_120-group_294 |
| Rv2283-group_238-group_296 |
| group_214-fadD30-group_339 |
| group_313-Rv3725-group_102 |
| group_117-group_245-mamB |
| group_317-PE27A-PPE46 |
| group_109-group_416-moaD1 |
| rmlC-MTB0006-Rv1586c |
| MTB0005-group_118-ctaE |
| glfT1-rfbD-Rv3785~~~fcl |
| Rv0374c-Rv0375c-secE2 |
| esxP-esxO-Rv2345 |
| group_290-Rv1765c~~~Rv2015c-group_319 |
| Rv1896c-Rv1895-group_382 |
| group_126-group_299-Rv3638 |
| Rv1978-Rv1977-Rv1976c |
| group_114-group_289-Rv1804c |
| Rv3329-sigJ-group_311 |
| group_286-PPE58-group_187 |
| pks5-group_322-Rv1526c |
| Rv0401-mmpL1-group_136 |
| citA-SpmT-Rv0887c |
| Rv0805-cpsY-group_134 |
| Rv3120-cyp141-group_207 |
| thiC-kdpB~~~Rv0424c-Rv0426c |
| Rv2015c~~~Rv1765c-group_116-group_320 |
| lig~~~ligC-Rv3733c-tgs2 |
| Rv2078-Rv2077c-Rv2075c |
| group_110-group_290-PPE39 |
| group_217-group_240-group_312 |
| cyp128-lppN-Rv2280 |
| Rv1767-Rv1766-group_320 |
| Rv1998c-ctpF-ctpG |
| group_118-Rv2077c-Rv2076c |
| Rv0200-omamA-zmp1~~~Rv0197~~~napA |
| group_120-group_294-Cas2 |
| Rv1919c-PPE35-group_329 |
| Rv2807-group_207-Rv3129 |
| PPE31-PE20-group_132 |
| group_113-group_293-cut1 |
| cysE-Rv2336-mmpL9 |
| moaC3-Rv3324A~~~moaB1-group_298 |
| group_134-group_122-Rv0836c |
| qcrC-ctaE-group_118 |
| group_320-esxL~~~esxN-group_321 |
| PE_PGRS39-mmpL9-Rv2336 |
| eccA5-group_114-group_320 |
| Rv1319c-Rv1319c~~~Rv1318c-group_137 |
| vapC18-lppA~~~vapB18~~~lppB-lppB~~~lppA |
| moeX-group_113-group_293 |
| group_75-group_332-group_294 |
| Rv2026c-Rv2025c-group_118 |
| Rv3324A~~~moaB1-group_298-group_311 |
| gyrA-Rv0007-cwsA |
| group_238-cysE-cysK1 |
| mmpL5-cut1-group_110 |
| group_119-group_294-group_120 |
| Rv0196-zmp1~~~Rv0197~~~napA-omamA |
| Rv2809-group_119-group_273 |
| group_110-group_290-Rv1765c~~~Rv2015c |
| Rv3160c-group_183-group_252 |
| Rv0276-vapC25~~~vapC-PE_PGRS4~~~PE_PGRS3 |
| cut1-group_110-group_290 |
| Rv2348c-plcB~~~plcC-group_110 |
| eccD5-Rv1794-esxO |
| mmpS1-group_108-mmpL1 |
| group_118-MTB0005-mamB |
| group_346-Rv0123-Rv0122 |
| group_117-group_295-group_234 |

# Table S10. Panaroo output structural variants only found in lineage 4 isolates, including H37Rv.

| **Lineage 4 specific Panaroo structural variants added when H37Rv is included** |
| --- |
| group_116-PPE39-group_332 |
| Rv0841-Rv0842-Rv0843 |
| Rv2646-Rv2645-arsC |
| crgA-Rv0010c-ppiA |
| cwsA-ppiA-Rv0010c |
| Rv0813c-MTB0011-group_138 |
| Rv0064-Rv0063-celA1 |
| echA21~~~caiD-lipE-Rv3776 |
| Rv1732c-Rv1733c-Rv1734c |
| Rv0812-Rv0813c-MTB0011 |
| vapB1-Rv0064-Rv0063 |
| Rv3778c-Rv3777-Rv3776 |
| whiB1-Rv3218-Rv3217c |
| Rv2642-arsC-Rv2645 |
| Rv0839-pip-Rv0841 |

# Table S11. Table of global network characteristics as determined by Cytoscape network analyzer (N = 109 isolates) on Panaroo output gene adjacency graph

| **Global Network Characteristics** | | | | | |
| --- | --- | --- | --- | --- | --- |
| **Network Characteristic** | **Average** | | **Std. Deviation** | **Minimum** | **Maximum** |
| Size | 102.3 | 25.7 | | 1.0 | 110.0 |
| Degree | 2.4 | 1.5 | | 1.0 | 27.0 |
| Average Shortest Path Length | 46.6 | 23.7 | | 24.6 | 165.1 |
| Clustering Coefficient | 0.1 | 0.2 | | 0.0 | 1.0 |
| Betweenness Centrality | 0.0 | 0.01 | | 0.0 | 0.3 |
| Neighborhood Connectivity | 2.7 | 1.9 | | 2.0 | 23.0 |
| Radiality | -0.7 | 0.9 | | -5.1 | 0.1 |
| Closeness Centrality | 0.0 | 0.0 | | 0.0 | 0.0 |
| Eccentricity | 172.8 | 17.3 | | 153.0 | 230.0 |
| Topological Coefficient | 0.5 | 0.1 | | 0.0 | 1.0 |

# Table S12. Gene list for high structural variant clusters along pangenome network

| **Gene Membership of High Structural Variance Regions in Pangenome Network** | |
| --- | --- |
| **Gene** | **Cluster** |
| echA9 | A |
| echA8 | A |
| Rv1069c | A |
| Rv1087A | A |
| Rv1066 | A |
| Rv1073 | A |
| Rv1075c | A |
| Rv1063c | A |
| PE_PGRS19 | A |
| Rv1061 | A |
| PE_PGRS20~~~PE_PGRS19 | A |
| Rv1060 | A |
| pra | A |
| LipU~~~aes_1 | A |
| PE_PGRS21 | A |
| PE10~~~PE9 | A |
| lpqV | A |
| Rv1062 | A |
| group_399 | A |
| mca | A |
| celA2b | A |
| Rv1086 | A |
| group_188 | A |
| Rv1085c | A |
| Rv1084 | A |
| Rv1083 | A |
| Rv1081c | A |
| Rv1065 | A |
| greA | A |
| metB | A |
| cbs | A |
| Rv1072 | A |
| fadA3 | A |
| Rv0025 | B |
| fhaA | B |
| MTB0010 | B |
| gyrB | B |
| Rv0068 | B |
| pknD_1~~~pknA | B |
| group_350 | B |
| Rv0042c | B |
| group_287 | B |
| whiB5 | B |
| Rv0021c | B |
| Rv0064 | B |
| yidC | B |
| mtgA~~~Rv0049 | B |
| Rv0039c | B |
| sdaA | B |
| ppiA | B |
| group_182 | B |
| Rv0067c | B |
| icd2 | B |
| Rv0051 | B |
| ponA1 | B |
| vapC1 | B |
| group_89 | B |
| group_349 | B |
| Rv0060 | B |
| Rv0059 | B |
| Rv0037c | B |
| Rv0038 | B |
| dnaB | B |
| rplI | B |
| rpsR1 | B |
| rpsF | B |
| celA1 | B |
| Rv0007 | B |
| Rv0048c | B |
| Rv0047c | B |
| group_411 | B |
| ino1 | B |
| ssb | B |
| Rv0004 | B |
| cwsA | B |
| Rv0044c | B |
| Rv0043c | B |
| leuS | B |
| mtc28 | B |
| Rv0073 | B |
| Rv0031 | B |
| Rv0071 | B |
| Rv0034 | B |
| acpA | B |
| group_224 | B |
| Rv0030 | B |
| group_104 | B |
| fadD34 | B |
| Rv0045c~~~menH_1 | B |
| Rv0029 | B |
| Rv0027 | B |
| Rv0063 | B |
| group_283 | B |
| Rv0024 | B |
| Rv0023 | B |
| Rv0061c | B |
| fipA | B |
| pstP | B |
| rodA | B |
| glyA2 | B |
| pbpA | B |
| Rv0057 | B |
| pknB | B |
| Rv0010c | B |
| Rv0028 | B |
| trpG | B |
| dnaA | B |
| crgA | B |
| Rv0026 | B |
| Rv0012 | B |
| Rv0052 | B |
| Rv0074~~~hutI | B |
| gyrA | B |
| bioF2 | B |
| Rv0036c | B |
| Rv0072 | B |
| recF | B |
| dnaN | B |
| vapB1 | B |
| group_225 | C |
| group_176 | C |
| group_175 | C |
| esxE | C |
| group_209 | C |
| Rv3900c | C |
| group_304 | C |
| cpnT | C |
| Rv3899c | C |
| group_376 | C |
| Rv3901c | C |
| ift | C |
| fadD19 | D |
| ilvX | D |
| group_190 | D |
| group_169 | D |
| group_334 | D |
| group_67 | D |
| PE_PGRS57 | D |
| PE_PGRS53 | D |
| PE_PGRS54 | D |
| Rv3510c | D |
| group_266 | D |
| group_361 | D |
| PE_PGRS56~~~PE_PGRS55 | D |
| group_244 | D |
| group_348 | D |
| PE25 | E |
| PPE59~~~PPE57 | E |
| PPE58 | E |
| alr | E |
| group_187 | E |
| group_393 | E |
| PPE59 | E |
| group_48 | E |
| group_301 | E |
| Rv3427c | E |
| Rv3427c~~~Rv3428c | E |
| group_286 | E |
| group_165 | E |
| tsaE | E |
| sigJ | F |
| moaX | F |
| group_108 | F |
| PPE55 | F |
| group_83 | F |
| Rv3327~~~Rv0797 | F |
| moaA | F |
| group_140 | F |
| group_311 | F |
| group_136 | F |
| group_203 | F |
| group_300 | F |
| group_298 | F |
| PPE46 | G |
| PPE46~~~PPE47 | G |
| group_122 | G |
| PE29 | G |
| esxS | G |
| PPE47~~~PPE48 | G |
| esxR | G |
| esxQ | G |
| group_317 | G |
| group_235 | G |
| group_134 | G |
| PPE47 | G |
| PE27A | G |
| pptT | H |
| Rv2808 | H |
| group_297 | H |
| Rv2817c | H |
| truB | H |
| Rv2799 | H |
| Rv2792c | H |
| Cas2 | H |
| Rv2767c | H |
| Rv2735c | H |
| recX | H |
| fadE21 | H |
| ftsK | H |
| Rv2787 | H |
| hsdS1 | H |
| rpsO | H |
| lppU | H |
| pepR | H |
| Rv2781c | H |
| Rv2778c | H |
| group_127 | H |
| sirR | H |
| Rv2772c | H |
| thyA | H |
| Rv2766c | H |
| group_163 | H |
| Rv2771c | H |
| PE27 | H |
| Rv2765 | H |
| Rv2819c | H |
| ltp1 | H |
| dfrA | H |
| pgsA3 | H |
| hsdS | H |
| vapB42 | H |
| vapC42 | H |
| thyX | H |
| hsdM | H |
| group_366 | H |
| dapA | H |
| Rv2752c | H |
| Rv2805 | H |
| Rv2751 | H |
| group_285 | H |
| Rv2750 | H |
| Rv2803 | H |
| Rv2762c | H |
| gpsI~~~pnp | H |
| mazE9 | H |
| Rv2749 | H |
| vapB21 | H |
| Rv2779c | H |
| Rv2797c | H |
| clgR | H |
| pspA | H |
| Rv2777c | H |
| Rv2743c | H |
| dapB | H |
| vapC21 | H |
| group_391 | H |
| ephG | H |
| Rv2739c | H |
| Rv2738c | H |
| ribF | H |
| recA | H |
| PE_PGRS47 | H |
| group_15 | H |
| group_273 | H |
| Rv2798c | H |
| Rv2804c | H |
| group_251 | H |
| Rv2802c | H |
| PPE44 | H |
| group_344 | H |
| Rv2776c | H |
| ald | H |
| Rv2775 | H |
| group_119 | H |
| Rv2821c | H |
| Rv2813 | H |
| Rv2820c | H |
| Rv2818c~~~csm6 | H |
| Rv2812 | H |
| Rv2811 | H |
| Rv2791c | H |
| Rv2809 | H |
| Rv2806 | H |
| argA | H |
| mazF9 | H |
| PPE43 | H |
| Rv2800 | H |
| lppV | H |
| Rv2795c | H |
| Rv2651c | I |
| Rv2646 | I |
| Rv2652c | I |
| Rv2658c | I |
| Rv2653c | I |
| Rv2647 | I |
| Rv2657c | I |
| arsC | I |
| Rv2663 | I |
| Rv2661c | I |
| group_88 | I |
| group_192 | I |
| Rv2659c | I |
| Rv2660c | I |
| Rv2656c | I |
| Rv2654c | I |
| Rv2650c | I |
| group_44 | I |
| Rv2655c | I |
| Rv2645 | I |
| group_110 | J |
| group_243 | J |
| group_321 | J |
| glyS | J |
| PPE40 | J |
| group_380 | J |
| group_336 | J |
| group_241 | J |
| group_335 | J |
| group_330 | J |
| group_162 | J |
| Rv2435c | J |
| group_161 | J |
| PPE39 | J |
| Rv0963c | J |
| PPE38 | J |
| group_386 | J |
| group_332 | J |
| group_290 | J |
| plcC | J |
| group_339 | J |
| group_395 | J |
| group_373 | J |
| group_257 | K |
| Rv1978 | K |
| Rv1976c | K |
| Rv1974 | K |
| group_397 | K |
| yrbE3A | K |
| Rv1973 | K |
| Rv1975 | K |
| mce3F | K |
| mpt64 | K |
| group_326 | K |
| group_73 | K |
| group_428 | K |
| lprM | K |
| group_423 | K |
| Rv1972 | K |
| mce3D | K |
| mce3B | K |
| mce3A | K |
| group_4 | K |
| mce3R | K |
| Rv1977 | K |
| Rv1979c | K |
| group_233 | K |
| group_378 | K |
| mce3C | K |
| nrdF1 | K |
| yrbE3B | K |

# Table S13. Table of Counts Of Isolates By Drug Resistance Phenotype (resistant:R, susceptible:S, Not Determined) across Antibiotics

| **Table of Isolate Counts with Drug Resistance Phenotypes** | | | |
| --- | --- | --- | --- |
| **Antibiotic** | **Resistant** | **Susceptible** | **Not Determined** |
| Pyrazinamide | 63 | 18 | 29 |
| Fluoroquinolone | 56 | 10 | 44 |
| Oflaxacin | 59 | 27 | 24 |
| Mofloxacin | 56 | 12 | 42 |
| Kanomycin | 65 | 25 | 20 |
| Capreomycin | 44 | 46 | 20 |
| Amikacin | 47 | 32 | 31 |
| Rifampicin | 68 | 39 | 3 |
| Isoniazid | 73 | 33 | 4 |

Figure S1. Gene ontology functional enrichment analysis of core genome from our pangenome analysis. -log10(*p*-value) reported and tests split by biological process, cellular component, and molecular function.


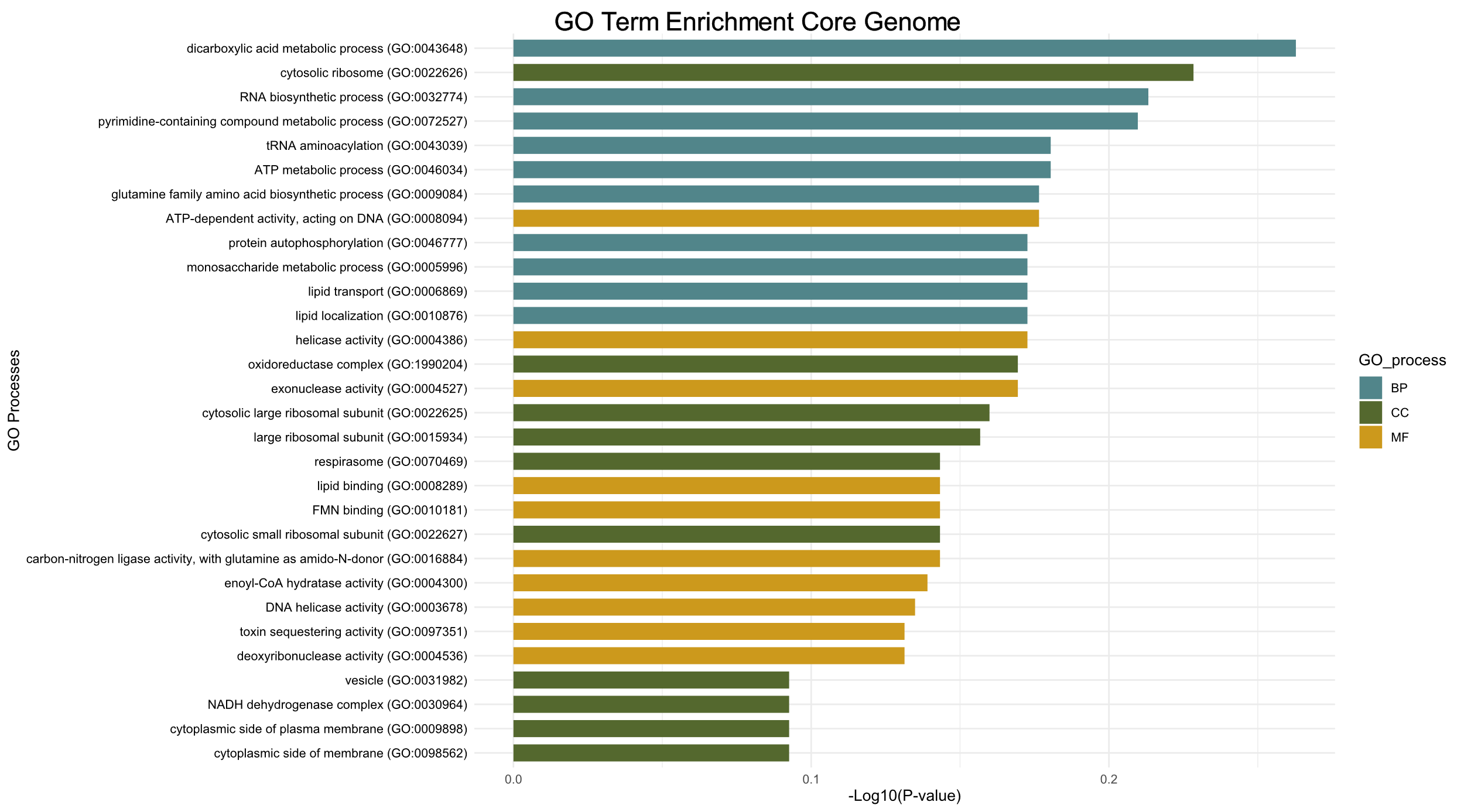


Figure S2. Correlation plot and loadings plot from the principal component analysis of the pangenome**.** **A**. Genes identified as significant and strongly correlated with the principal components were confirmed by Pearson correlation r^2^ values. Asterisks indicate significance with levels including 0.0001****, 0.001***, 0.01** and 0.05* and color gradient correlates to strength of correlation. **B.** Loadings plot illustrates genes with the highest loadings by the first five principal components. Color gradient shows genes with very positive (light red) to very negative (light green) correlations with the principal component.


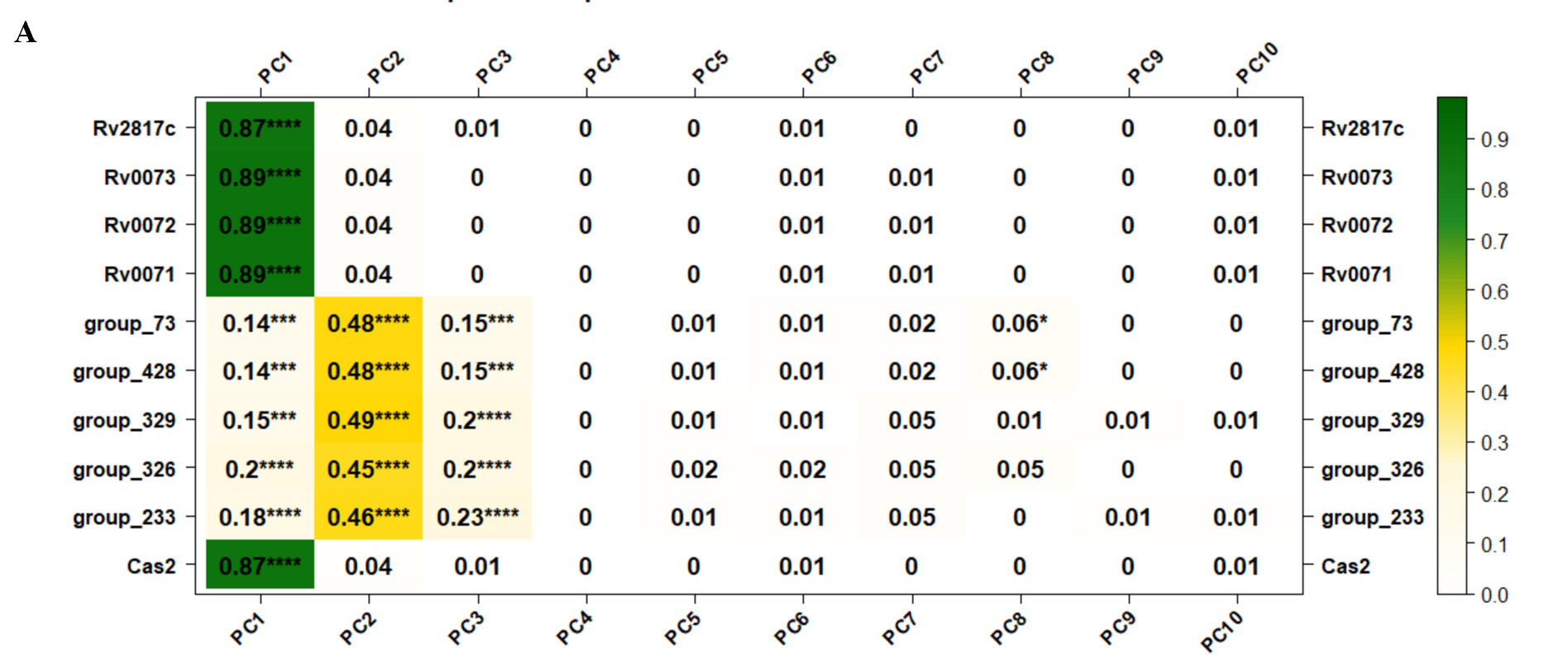


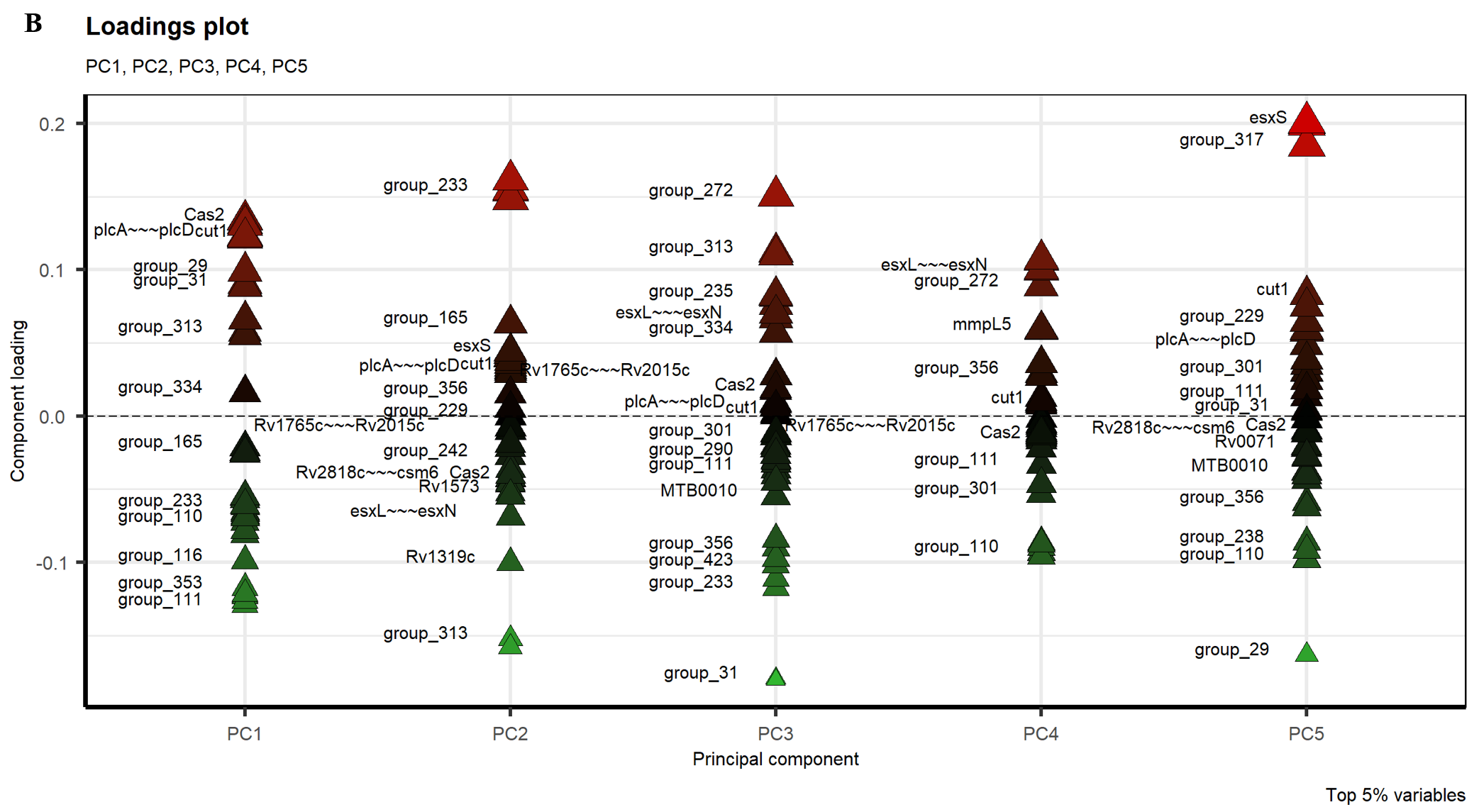


Figure S3. Randomized permutation test with replacement to assess likelihood of enrichment of unannotated genes in the top 5% of the PCA loadings (N=10,000)

**A**


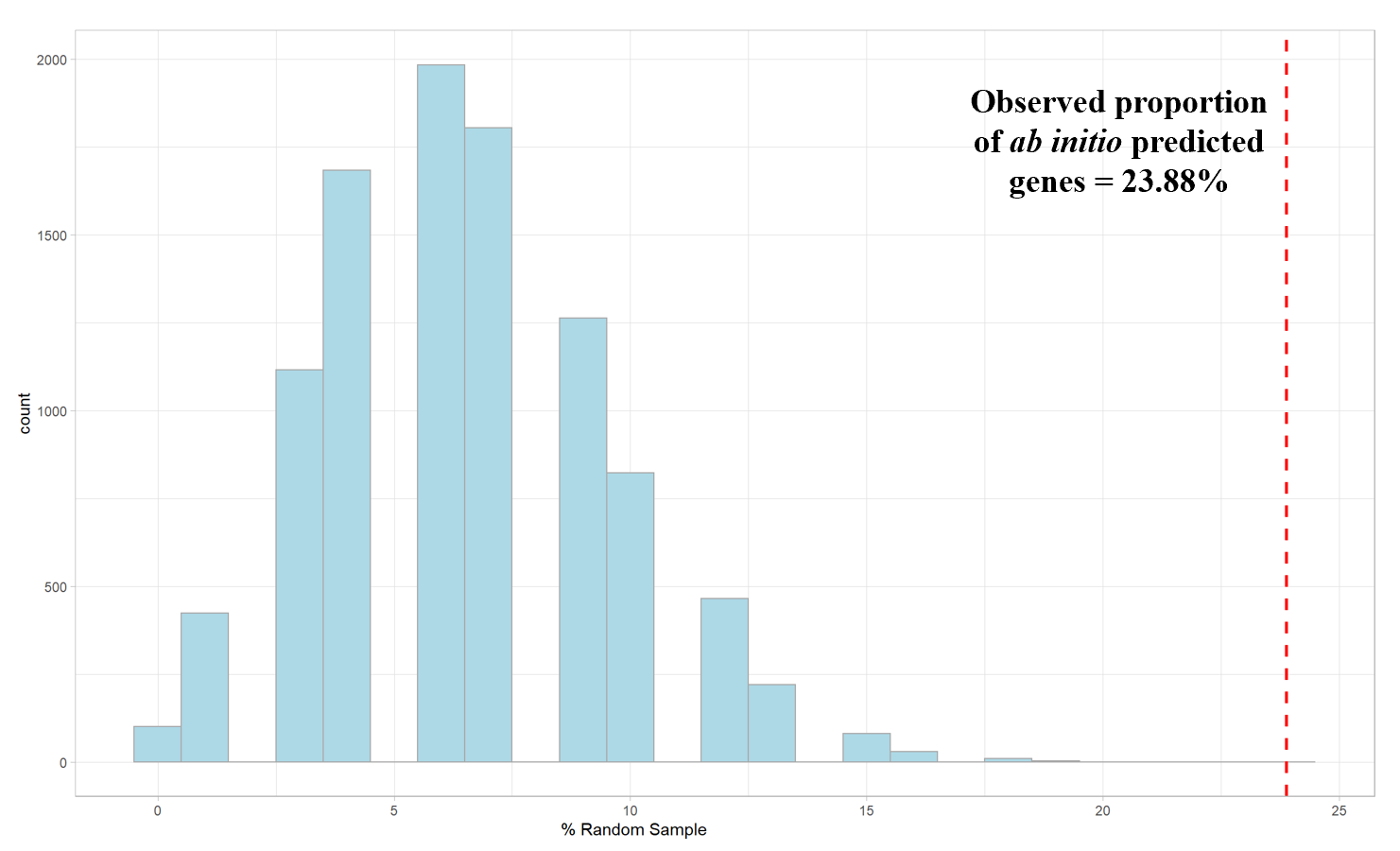


**B**


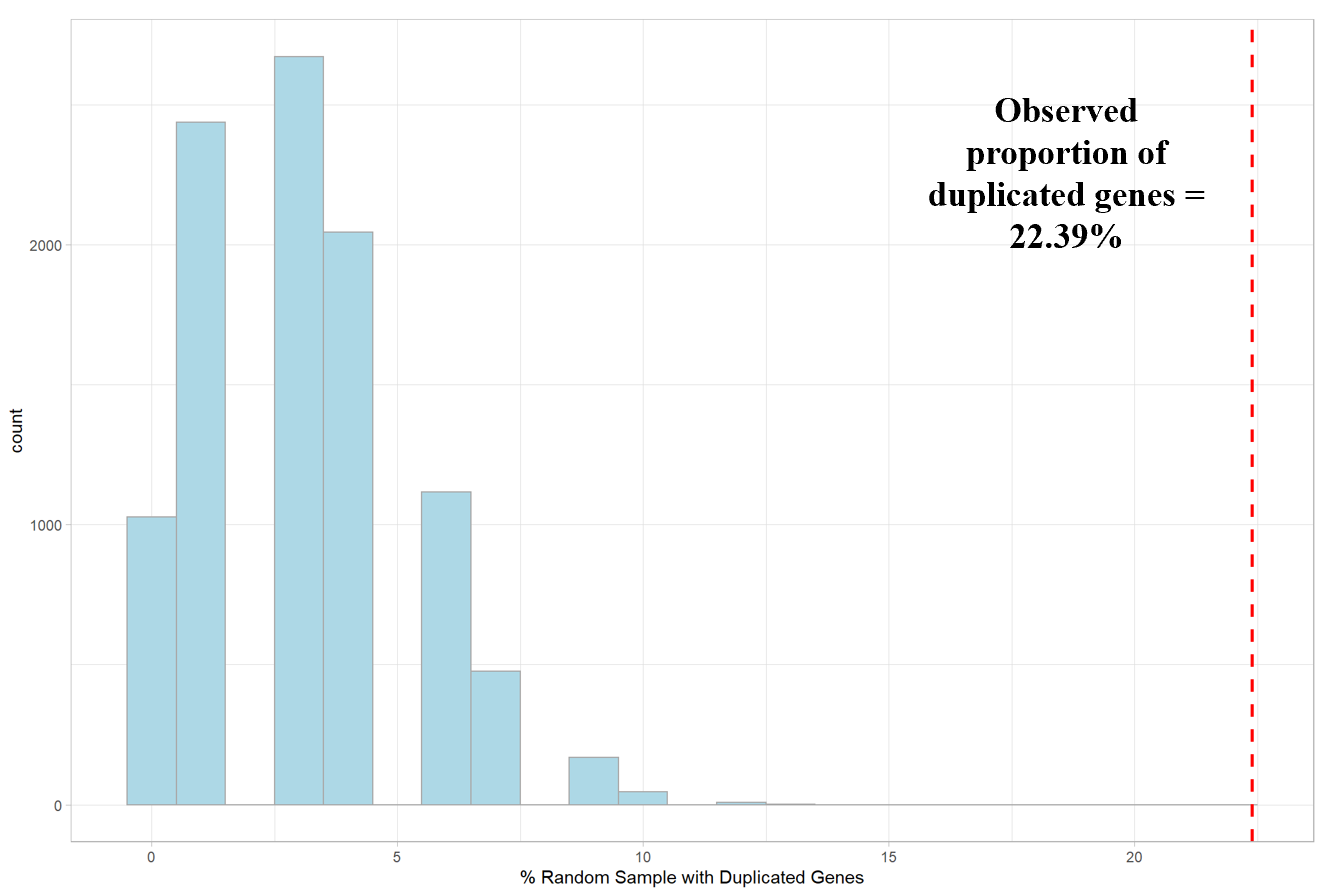


Figure S4. Lineage-specific pangenome Sankey diagrams (Lineages 1-4)


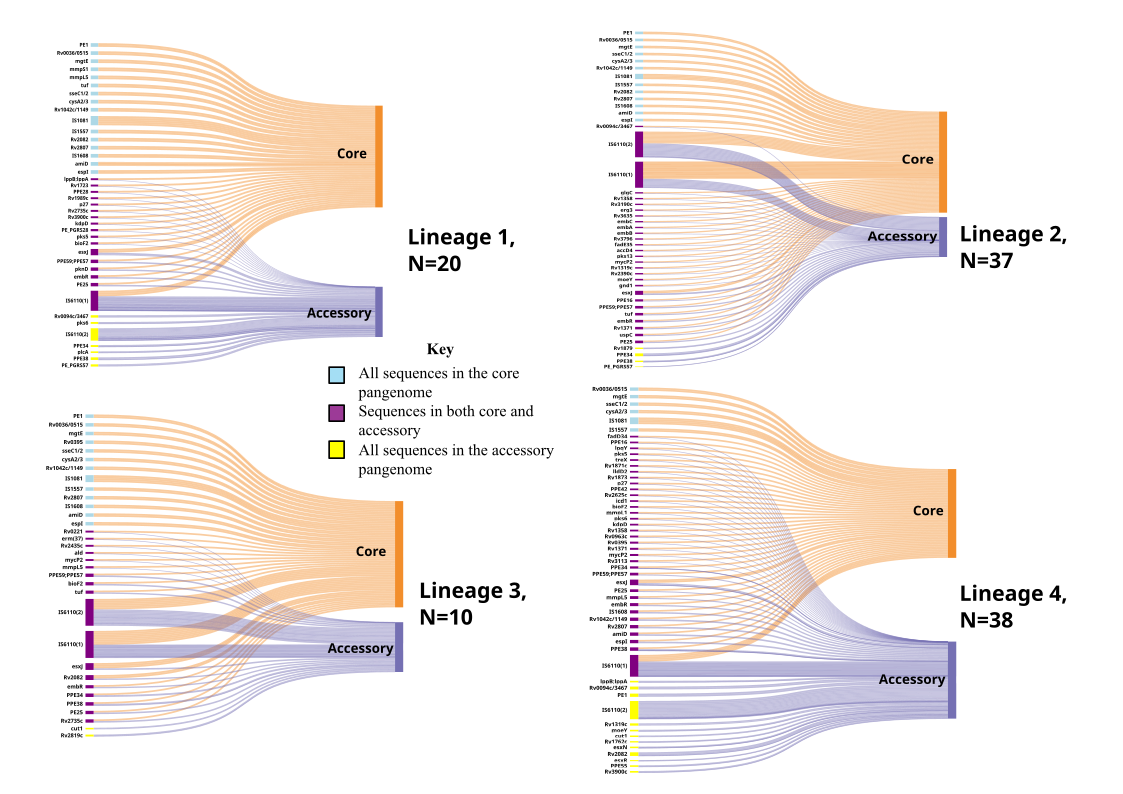


Figure S5. Average copy number of genes with duplicates in the lineage-specific pangenomes. Color gradient indicates number of gene copies. Copy numbers reflect average number of gene copies across isolates in the lineage. Genes are commonly duplicated across lineages, but copy numbers are variable, especially among IS elements.


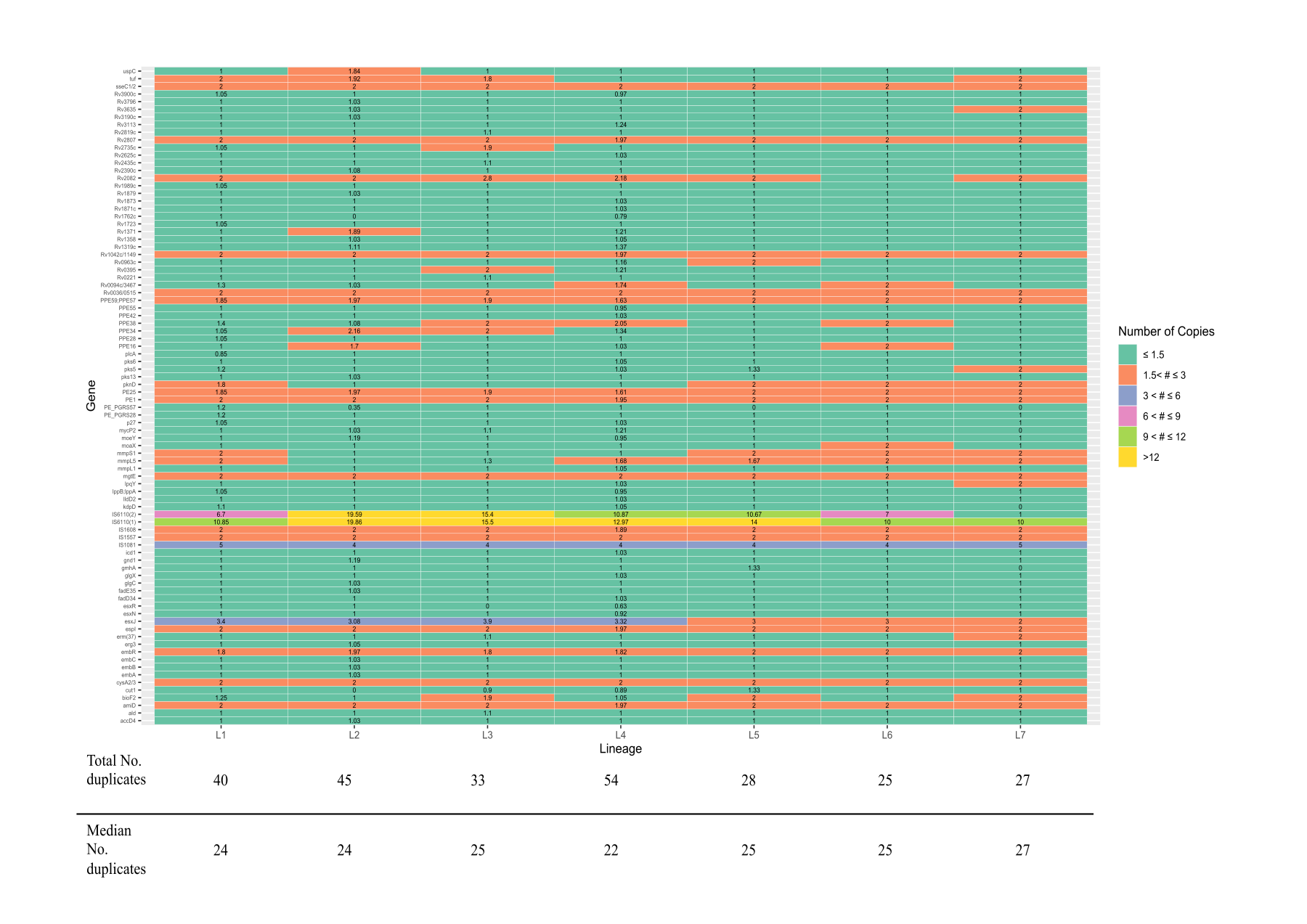


Figure S6. Distribution of the number of isolates with a given fragmented gene, N = 565 genes. Genes with multiple different genes present at a given locus (i.e., merged annotations) were not included in this analysis. Most genes were only fragmented in one or a few isolates.


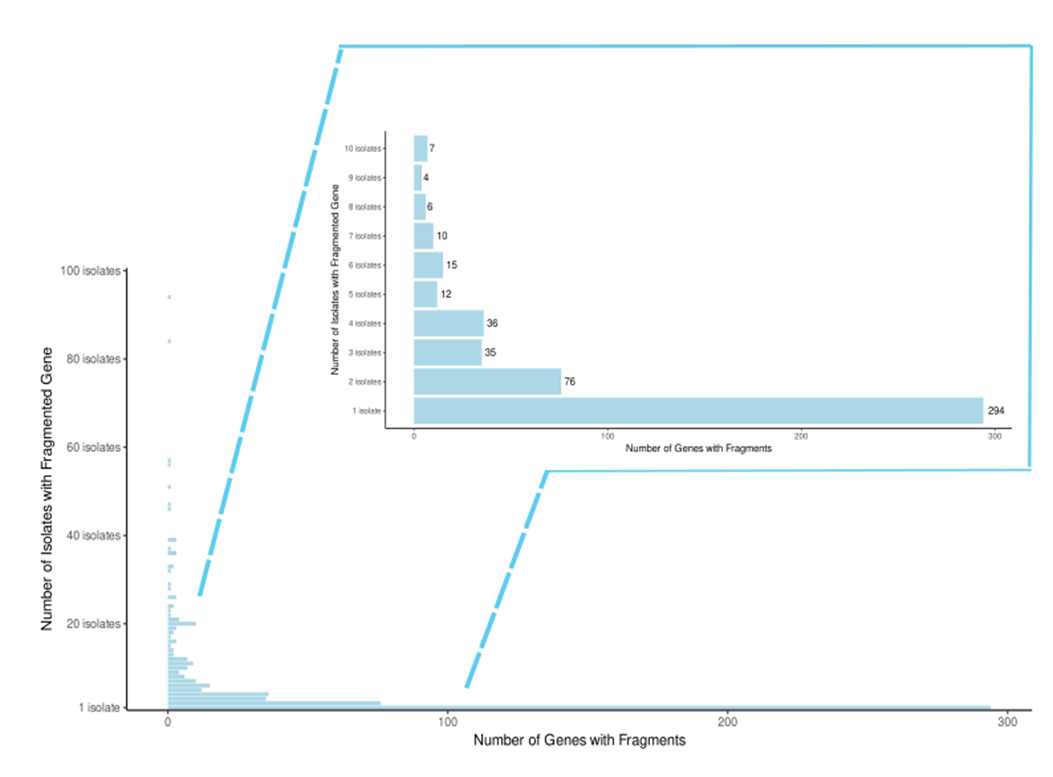


# Figure S7. Distribution of fragmented genes per genome by lineage


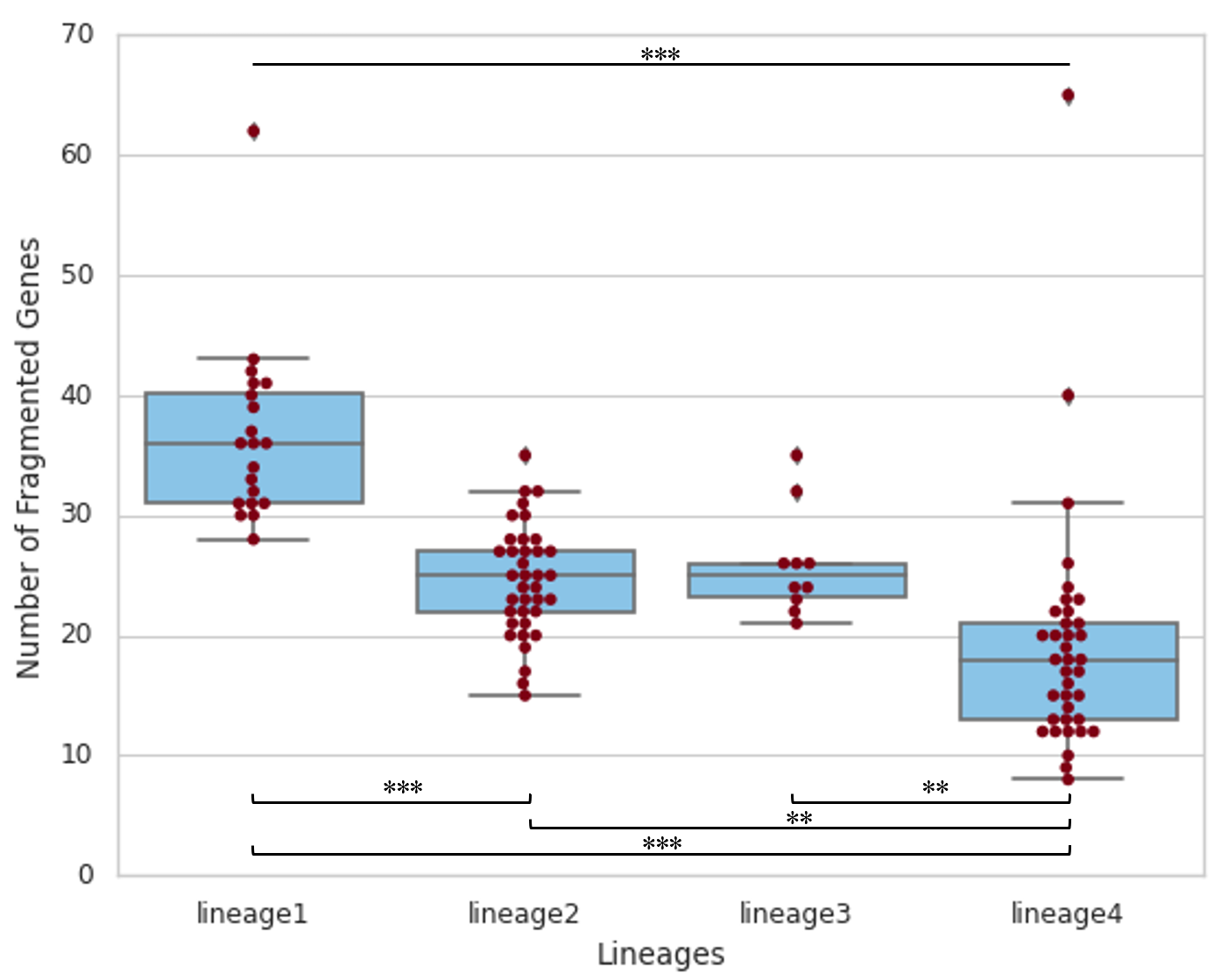


****p*-value < 0.001

***p*-value < 0.05

Figure S8. Panaroo merged annotations among two genes. Many merged annotations (i.e., “geneA~~~geneB”) present as being a single gene interchangeable at a given locus in the pangenome (A), although there are also interchangeable genes with duplications (B), merged annotations of different genes (C), and genes annotated as “*ab initio* predictions” by Prokka and corrected by Panaroo (D). Numbers of genes with these gene arrangements are not mutually exclusive and can have a mixture of these different gene arrangements in individual isolates at a given locus in the pangenome.


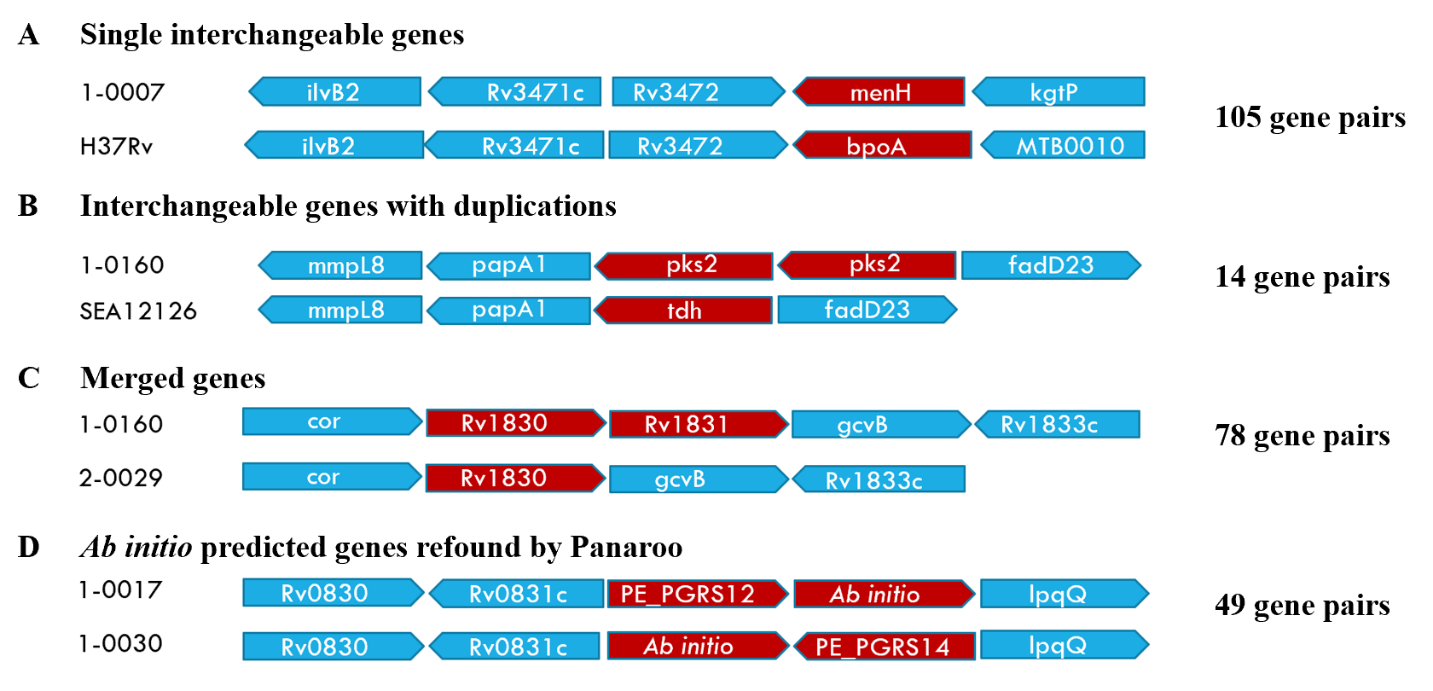


# Figure S9. Hierarchical clustering of high structural variant region genes parsed by high structural variant cluster and lineage.


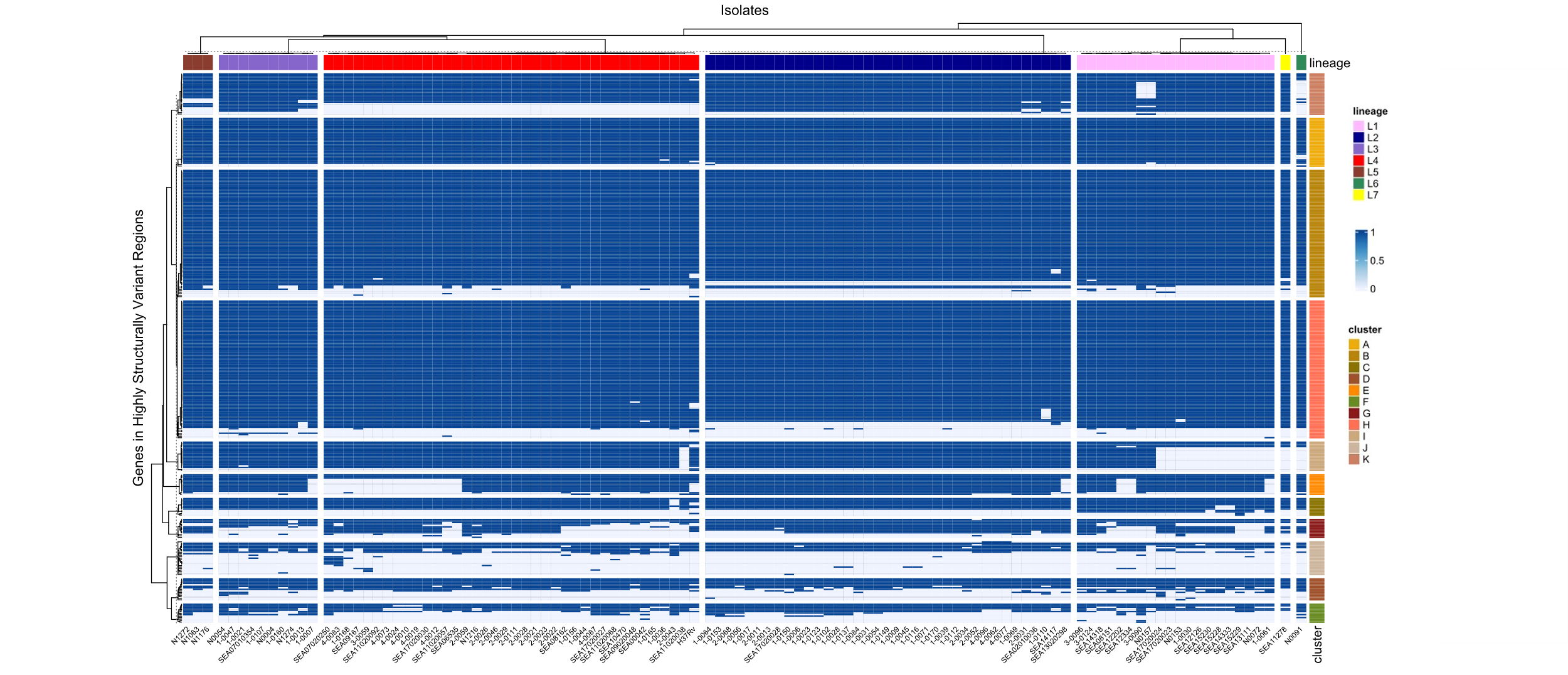


# Figure S10. Overviews of lineage-specific networks composed of gene contributors to structural variants found by Panaroo.


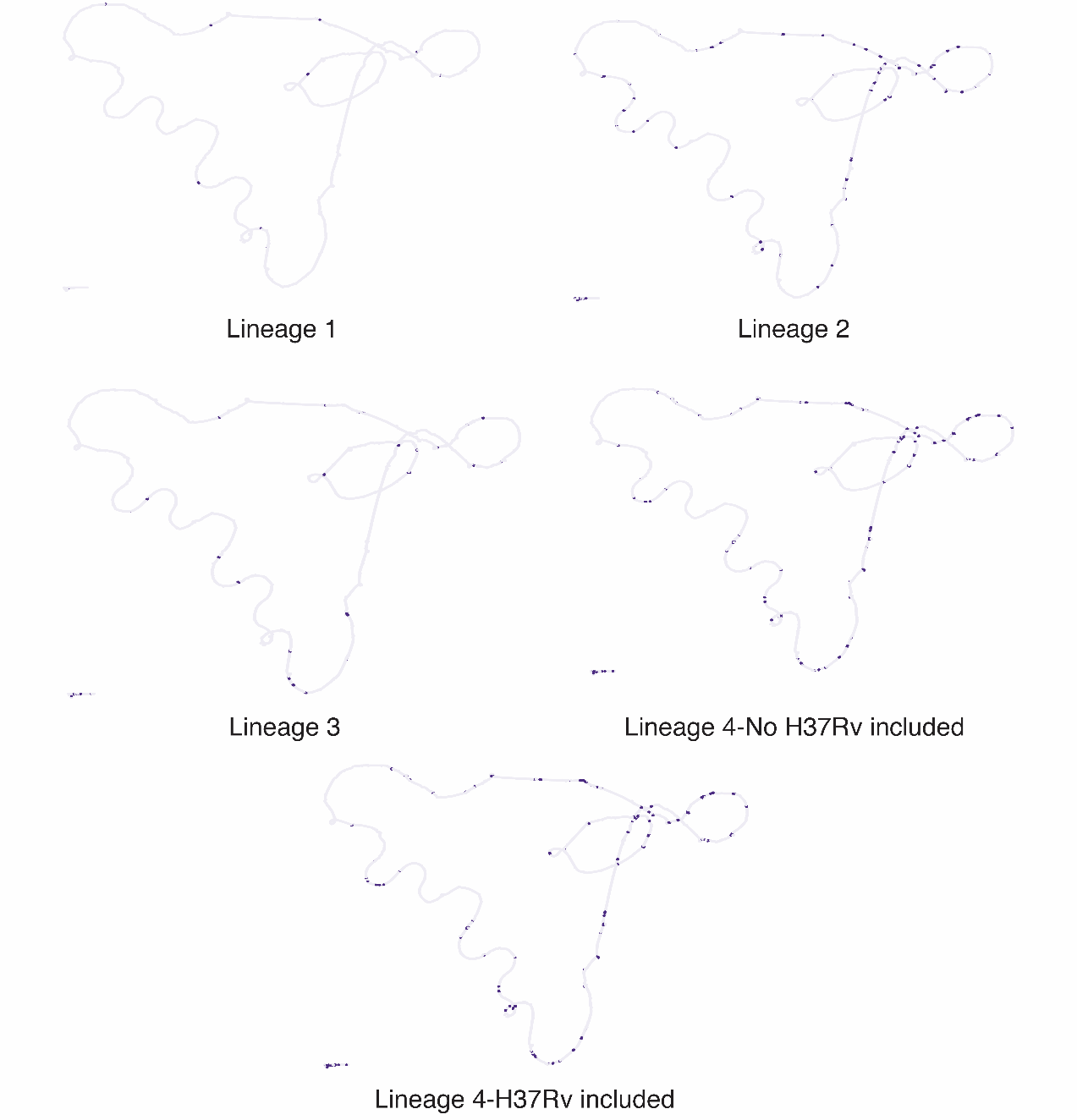


Figure S11. Scatterplot of sample size and core genome size reported by other studies and this study, demonstrating the tradeoff between sample size and reported core genome.


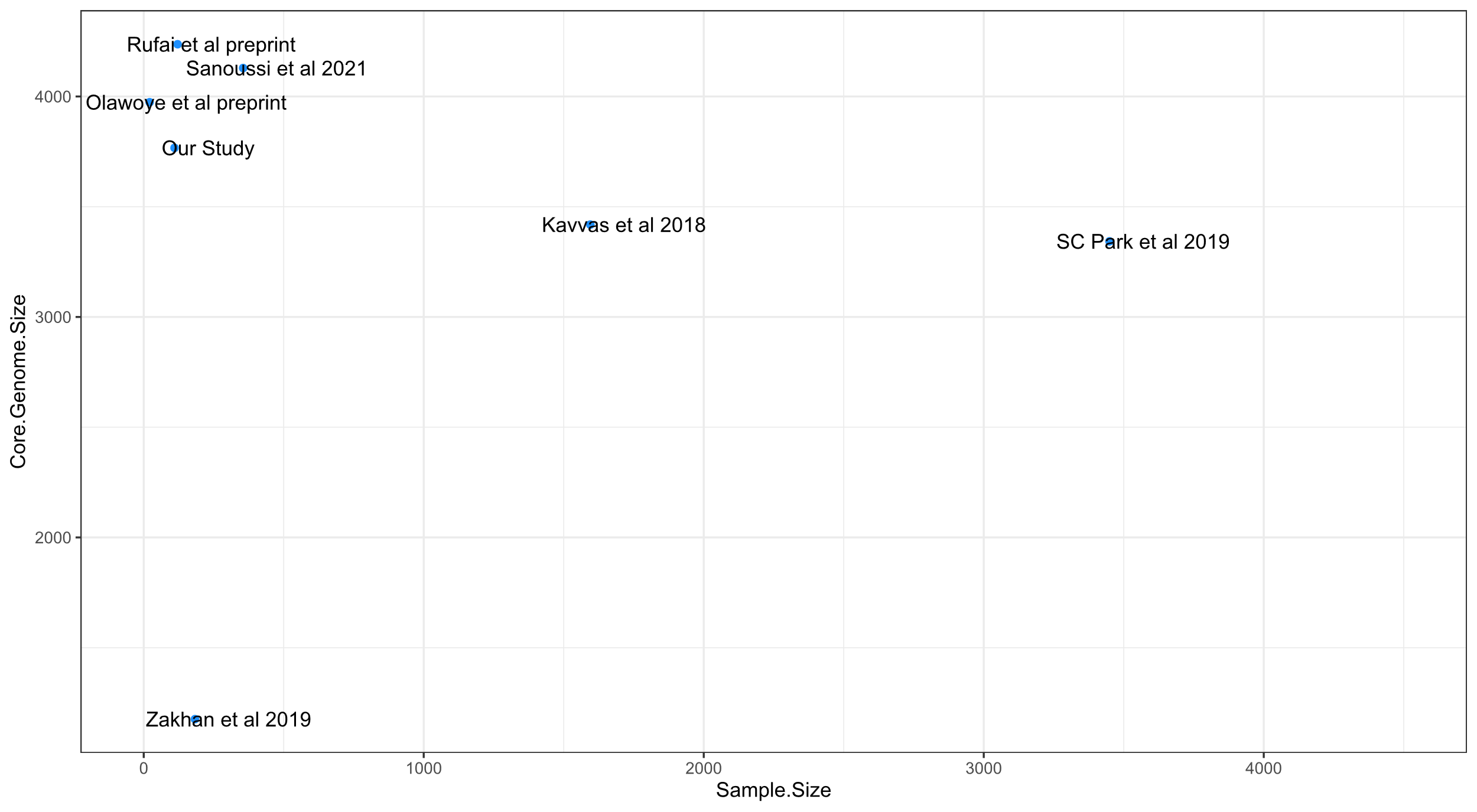


Figure S12. Quality of 109 M. tuberculosis clinical isolates de novo assemblies as measured by numbers of SNPs and single-base insertions and deletions with respect to H37Rv**.** Isolates that diverge more from the reference are expected to have more variants, but SNPs and indels should be proportionally increasing. The upper graph shows all 109 isolates, while the lower graph excludes the four labeled outliers (2-0111, 4-0083, 1-0165, and 2-0026) and demonstrates a linear correlation among the remaining genomes.


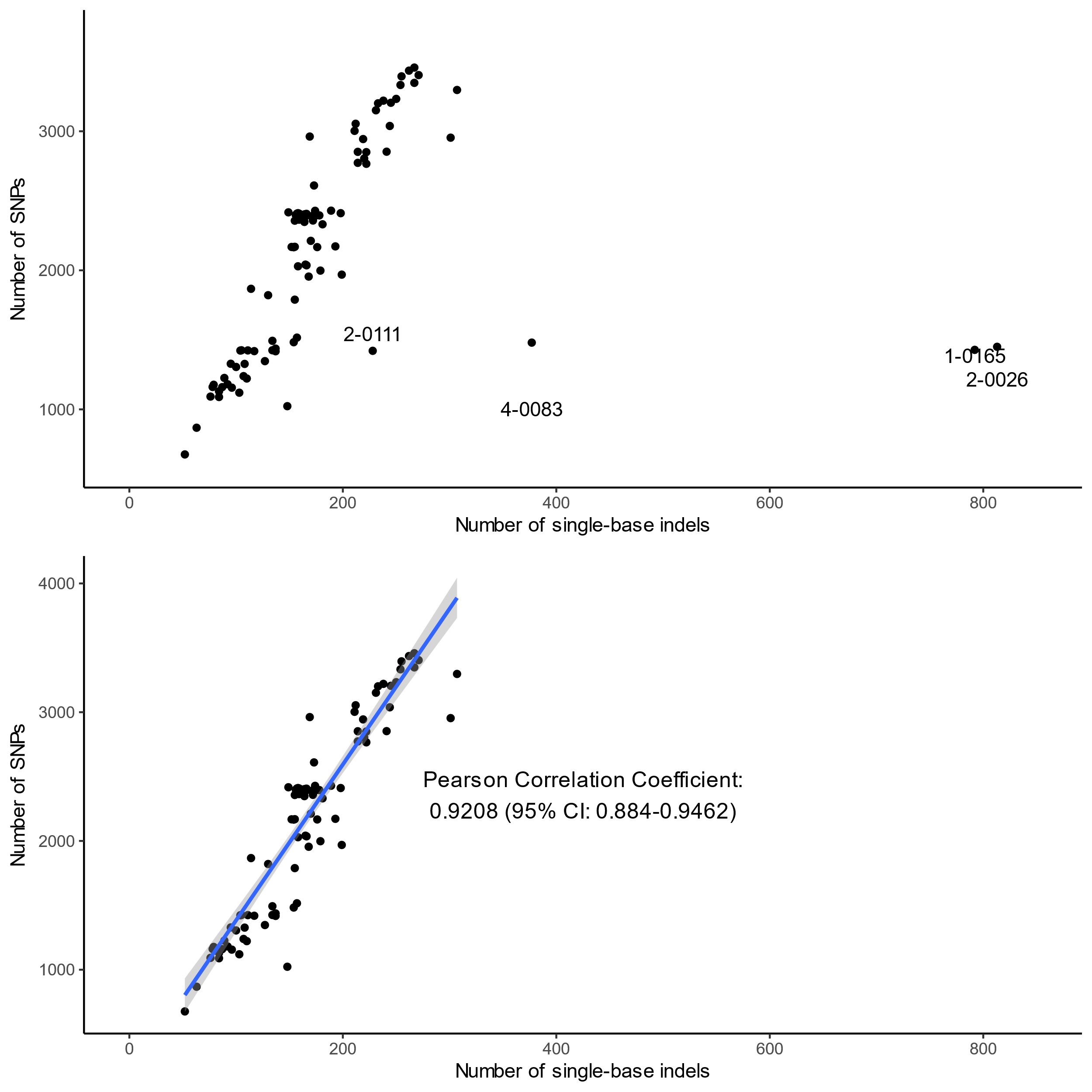


# Supplemental Methods

## Genome Assembly with Quality Control

Details of each step of our genome assembly pipeline are as follows:

1. *Assembly:* For isolates that were sequenced on multiple SMRT cells (e.g. multiple SRA entries sharing the same isolate name), all SMRT cell sequencing runs were combined. Hierarchical Genome Assembly Process (1) (HGAP) version 2 (RS_HGAP_Assembly.2 protocol) was used to assemble raw reads from HDF5 formatted files using default parameters. If HGAP2 was unable to assemble the genome (e.g. multiple contigs or no contig could be circularized [see #2]) or a misassembly was detected (see #4), the assembler Canu (2) (v1.6) was used with default parameters with the exception of the -pacbio-raw flag. Canu was confirmed to be more accurate in assembly than several assemblers and was, therefore, the assembler of choice for isolates sequenced in 2017 (the publication year of Canu). Any genome that failed assembly using Canu was excluded from this study.
2. *Circularization:* For isolates sequenced prior to 2017, a pipeline using custom Perl scripts was in place to prepare contigs for minimus2 (from the AMOS package, <http://amos.sourceforge.net>) to circularize each genome, and then to reset the genome start so that *dnaA* is the first gene, as described previously (3). However, minimus2 could not successfully circularize all genomes due to complexity in the contig edges preventing a clean overlap. For these cases, circlator(4) was used to circularize using the ‘all’ command and the --genes_fa flag set to the H37Rv *dnaA* sequence to reset the genome order, default parameters were used otherwise. All isolates sequenced in 2017 or later were directly circularized with circlator. Any genome that failed to circularize after this step was excluded from this study.
3. *Consensus polishing:* Consensus polishing is an important step in producing a final consensus sequence of high-accuracy, particularly since HGAP2 and Canu are not sensitive enough to call consensus in repetitive regions at the nucleotide level ([Why iterate the mapping, quiver/arrow process](https://github.com/PacificBiosciences/GenomicConsensus/blob/develop/doc/FAQ.rst#why-would-i-want-to-iterate-the-mappingquiverarrow-process)). Polishing was performed as previously described(3). Briefly, the circularized sequence was polished using BLASR+Quiver (RS_Resequencing protocol) in SMRTAnalysis (v2.3) to achieve a complete consensus sequence; three rounds were determined to be sufficient in most cases to attain a consensus sequence ([Iterating the mapping quiver/arrow process is not convergent](https://web.archive.org/web/20170813103132/https:/github.com/PacificBiosciences/GenomicConsensus/blob/develop/doc/FAQ.rst#is-iterating-the-mappingquiverarrow-process-a-convergent-procedure)). The maximum coverage parameter in Quiver was set to 1000, default parameters were used otherwise. If a consensus sequence could not be achieved after three rounds of polishing, it was excluded from this study.
4. *Assembly Quality Control:* In order to validate the assembly, each genome was analyzed for potential breaks using PBHoney (5) and errors in consensus calling (see #3 above). We considered regions as “breaks” in PBHoney when the variant was supported by at least 10% of the total coverage at the location in the genome. Assemblies that failed this step (had breaks with at least 10% support) were excluded from further analysis in this study. Verifying the final consensus sequence was included in QC since iterating over polishing rounds is not a convergent process for repetitive genomes ([Iterating the mapping quiver/arrow process is not convergent](https://web.archive.org/web/20170813103132/https:/github.com/PacificBiosciences/GenomicConsensus/blob/develop/doc/FAQ.rst#is-iterating-the-mappingquiverarrow-process-a-convergent-procedure)).

In the final sequence, an overrepresentation of single-base indels suggests a particularly noisy sequencing run, potentially due to either overloading or underloading the SMRT Cell. In comparing the final assemblies to the H37Rv reference genome (NC_000962.3), we expect that the number of SNPs and single-base indels should be roughly proportional across strains, with single-base indels accumulating at a frequency roughly commensurate with SNPs. This was only observed in four of the 109 genomes: 2-0111, 4-0083, 1-0165, and 2-0026. The remaining 105 show a strong linear correlation (*R* = 0.921) between the two variant types (Figure S12). All 109 isolates were kept in our analyses due to their performance on other quality control metrics and variabilities in these 4 did not greatly impact findings in study.

# References

1. Chin C-S, Alexander DH, Marks P, Klammer A a, Drake J, Heiner C, et al. Nonhybrid, finished microbial genome assemblies from long-read SMRT sequencing data. Nat Methods. 2013 Jun;10(6):563–9.

2. Koren S, Walenz BP, Berlin K, Miller JR, Bergman NH PA. Canu: scalable and accurate long-read assembly via adaptive k-mer weighting and repeat separation. Genome Res. 2017;27:722–36.

3. Elghraoui A, Modlin SJ, Valafar F. SMRT genome assembly corrects reference errors, resolving the genetic basis of virulence in Mycobacterium tuberculosis. BMC Genomics. 2017;18(1):1–14.

4. Hunt M, Silva N De, Otto TD, Parkhill J, Keane JA, Harris SR. Circlator: automated circularization of genome assemblies using long sequencing reads. Genome Biol. 2015 Dec;16:294.

5. English AC, Salerno WJ, Reid JG. PBHoney: identifying genomic variants via long-read discordance and interrupted mapping. BMC Bioinformatics. 2014 Jun;15:180.
